# Genome Mining and Heterologous Reconstitution of a PKS-NRPS Gene Cluster from *Aspergillus flavipes* LY1-5 Affords Structurally Novel Tetronates

**DOI:** 10.3390/jof12010028

**Published:** 2025-12-29

**Authors:** Quan Dai, Yiqiao Li, Shuzhe Lv, Shuang Zhao, Liyuan Han, Jiaxin Xu, Hui Shuai, Youming Zhang, Fu Yan

**Affiliations:** 1 State Key Laboratory of Microbial Technology, Shandong University, Qingdao 266237, China; 2 Hunan Provincial Key Laboratory of Microbial Molecular Biology, College of Life Science, Hunan Normal University, Changsha 410081, China; 3 Shenzhen Key Laboratory of Genome Manipulation and Biosynthesis, Key Laboratory of Quantitative Synthetic Biology, Shenzhen Institute of Synthetic Biology, Shenzhen Institutes of Advanced Technology, Chinese Academy of Sciences, Shenzhen 518055, China; 4 State Key Laboratory of Integration and Innovation of Classic Formula and Modern Chinese Medicine, Lunan Pharmaceutical Group Co., Ltd., Linyi 276005, China; 5 Rizhao Marine Biotechnology Center, Rizhao Polytechnic, Rizhao 276826, China

**Keywords:** tetronate, *Aspergillus flavipes* LY1-5, heterologous expression, talactones A–J, antibacterial activity

## Abstract

Heterologous expression of silent biosynthetic gene clusters represents a key strategy for the discovery of structurally novel natural products. In this study, we obtained ten new tetronate natural products, designated as talactones A–J (**1**–**10**), through heterologous expression of a polyketide synthase–nonribosomal peptide synthetase (PKS-NRPS) gene cluster (*tho*) from the fungus *Aspergillus flavipes* LY1-5 in *A. nidulans* A1145. Their structures were elucidated by comprehensive HR-ESI-MS and NMR analyses. Notably, talactone A (**1**) contains a rare 1,4-thiazepane scaffold, whereas talactones B (**2**) and C (**3**) feature a novel 2,3-dihydrofuro [3,4-*b*]pyridine-4,5(1*H*,7*H*)-dione skeleton. Biosynthetic investigations indicate that the 1,4-thiazepane ring in **1** arises from a non-enzymatic reaction between a tetronate acid and cysteine, while **2** and **3** are derived from **5** and **6**, respectively, via spontaneous intramolecular cyclization under acidic conditions. Antibacterial activity assays revealed that compounds **1**–**3**, **9**, and **10** exhibit moderate antibacterial effects.

## 1. Introduction

Fungi represent a rich reservoir of natural products, with approximately 47% of documented microbial natural products being derived from fungi, underscoring their considerable biosynthetic potential [1]. Among these, tetronates constitute a notable class of compounds distinguished by their unique structural features and biosynthetic pathways. Tetronates are a structurally diverse family of bacterial polyketide-derived natural products characterized by a tetronic acid (4-hydroxy-[5*H*]furan-2-one) core [2,3,4,5,6,7,8,9]. Their biosynthesis typically initiates with the assembly of a linear polyketide chain by modular polyketide synthases (PKSs), followed by incorporation of a glycerol-derived three-carbon unit to form the characteristic tetronate ring [5]. This linear precursor often undergoes extensive enzymatic tailoring, giving rise to two major subclasses: spirotetronates, which contain a distinctive spirocyclic junction between the tetronate ring and a cyclohexene or decalin system, and other structurally complex tetronates [2]. The formation of the intricate spirotetronate scaffold frequently involves key enzymatic cyclizations, such as the endo-selective intramolecular Diels–Alder (IMDA) reaction catalyzed by Diels–Alderases like LucM, as illustrated in the biosynthesis of lucensimycin A [4]. Alternatively, some pathways employ atypical mechanisms, as exemplified by LucK, which catalyzes a stereoselective nucleophilic spirocyclization rather than a conventional Diels–Alder reaction [4].

These complex structural frameworks are associated with a broad range of potent biological activities. Tetronates exhibit significant antibacterial and antitumor properties [6]. For instance, the glycosylated spirotetronate tetrocarcin A has emerged as a promising candidate for future evaluation in cancer therapy due to its ability to downregulate the expression of the tight junction protein Junctional Adhesion Molecule-A (JAM-A), attenuate tumorigenic signaling, and induce apoptosis [10]. Ongoing genome-mining efforts continue to uncover novel tetronate variants with unique structural modifications [11,12], providing a valuable foundation for drug discovery and development.

In this study, we performed genome mining on the fungus *Aspergillus flavipes* LY1-5 and identified a biosynthetic gene cluster (BGC) predicted to be involved in tetronate production. Heterologous expression of this BGC in the engineered host *A. nidulans* A1145 led to the identification of ten novel tetronate derivatives. These include compound **1**, which contains a rare 1,4-thiazepane skeleton, and compounds **2** and **3**, which feature a novel 2,3-dihydrofuro [3,4-*b*]pyridine-4,5(1*H*,7*H*)-dione scaffold.

## 2. Results and Discussion

**Figure 1:**
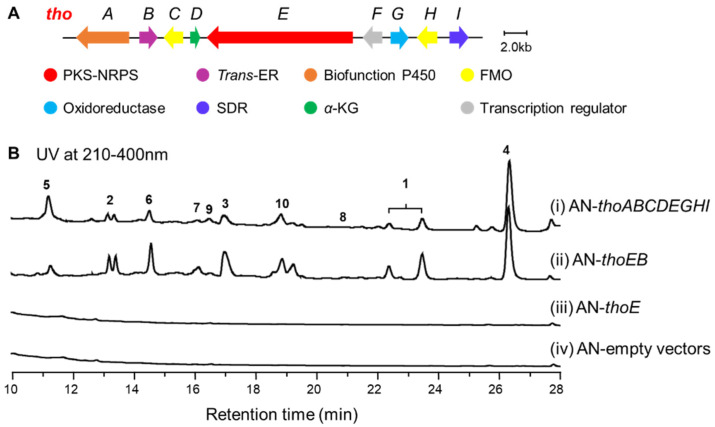
(**A**) The *tho* cluster from *A. flavipes* LY1-5. (**B**) HPLC traces of the EtOAc extracts derived from the *A. nidulans* A1145 mutant strains (i: AN-*thoABCDEGHI*, ii: AN-*thoEB*, iii: AN-*thoE*, and iv: AN-empty vectors).

*A. flavipes* LY1-5 was previously isolated from a soil sample. A comprehensive analysis of the *A. flavipes* LY1-5 genome using antiSMASH [13] and 2ndFind (https://biosyn.nih.go.jp/2ndfind, accessed on 5 October 2023) led to the identification of a putative BGC, designated as the *tho* gene cluster (Figure 1A). The core gene of this gene cluster is *thoE*, which encodes a hybrid polyketide synthase–nonribosomal peptide synthetase (PKS-NRPS). Domain analysis of ThoE revealed a modular architecture consisting of ketosynthase (KS), acyltransferase (AT), dehydratase (DH), C-methyltransferase (CMT), nonfunctional enoylreductase (ER^0^), ketoreductase (KR), acyl carrier protein (ACP), condensation (C), adenylation (A), peptidyl carrier protein (T), and a Dieckmann cyclase (D) domain. ThoE exhibits high sequence similarity (57.90% identity) to ThnA from the triharone BGC [14], suggesting a potential role in the biosynthesis of structurally related natural products. Flanking *thoE* are genes encoding auxiliary enzymes, including a *trans*-enoyl reductase (*trans*-ER, ThoB), a bifunctional cytochrome P450/NADPH-cytochrome P450 reductase (ThoA), two FAD-dependent monooxygenases (ThoC and ThoH), a transcriptional regulator (ThoF), an α-ketoglutarate-dependent oxygenase (ThoD), an oxidoreductase (ThoG), and a short-chain dehydrogenase/reductase (ThoI). These enzymes are hypothesized to participate in the tailoring and regulation of the biosynthetic pathway.

To characterize the metabolites produced by the *tho* gene cluster, we cloned and expressed the core gene *thoE* in the heterologous host *A. nidulans* A1145 but obtained no product. We then expressed *thoE* and *thoB* in *A. nidulans* A1145, which yielded a series of new products. Meanwhile, the auxiliary genes *thoACDGHI* were co-expressed with *thoE* and *thoB*, but no additional compounds were produced. Therefore, we performed compound isolation from the fermentation culture of *A. nidulans* A1145::*thoEB*, which led to the purification of ten compounds (Figure 1B). The planar structures of the compounds were determined through extensive HR-ESI-MS and 1D/2D NMR analyses (Figure 2 and Figures S3–S77). The elucidated structures of the compounds revealed that they are tetronate derivatives. They were designated as talactones A–J (**1**–**10**).

Talactone A (**1**) was isolated as a yellow powder. Its molecular formula was established as C_21_H_31_NO_8_S based on HR-ESI-MS data (*m/z* 440.1737 [M−H]^−^, calcd 440.1748), indicating seven degrees of unsaturation. The ^1^H NMR and HSQC data (Table 1) revealed characteristic signals for three methyls, an oxygenated methine, a thio-methine, and a thio-methylene. The DEPTQ and ^13^C NMR spectra (Table 1) confirmed 21 carbon resonances, categorized as three methyls, seven methylenes (including one sulfur-attached), five methines (one sulfur-, one oxygen-, and one nitrogen-bearing), and six nonprotonated carbons, comprising three olefinic carbons (one alkenyl and two carboxyls), one ester carbonyl, and one conjugated carbonyl. All proton–carbon correlations were unequivocally assigned via HSQC. The remaining unsaturations indicated that **1** contains two ring systems. Key HMBC correlations from H_2_-5 to C-6, C-4, and C-1; from H-4 to C-3; from H-2′ to C-2, C-1′, and C-3′; and from H_2_-7′ to C-5′, C-9′, C-11′, and C-12′, along with ^1^H–^1^H COSY correlations between H_2_-5/H-4, H-3′/H-2′/H-4′, H-5′/H-4′/H-6′, and H-7′/H-6′/H-8′ (Figure 3), confirmed the presence of a linear tetronate acid moiety. Three additional carbons—a carboxyl (*δ*_C_ 170.3, C-1″), a methine (*δ*_C_ 60.0, C-2″), and a methylene (*δ*_C_ 28.0, C-3″)—were identified in DMSO-*d*_6_. The key HMBC correlations from H-3″ to C-3′, C-2″, and C-1″; from H-2″ to C-1″ and C-3″; and from the NH proton (*δ*_H_ 11.54, d, *J* = 5.4 Hz) to C-2″, C-2, C-2′, and C-1′ (Figure 3), combined with the molecular formula, indicated that a cysteine residue is connected to C-3′ via a sulfur atom and to C-1′ via a nitrogen atom, forming a 1,4-thiazepane ring. This evidence conclusively established the planar structure of **1**.

**Figure 2:**
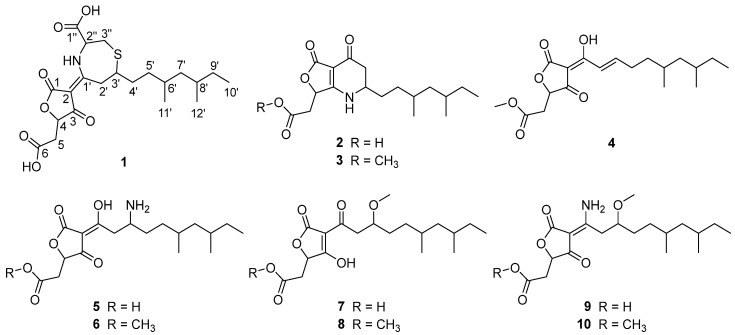
Structures of compounds **1**–**10**.

Talactone B (**2**) and talactone C (**3**) were obtained as yellow powders. HR-ESI-MS analysis established molecular formulae as C_18_H_27_NO_5_ for **2** (*m/z* 336.1807 [M–H]^−^) and C_19_H_29_NO_5_ for **3** (*m/z* 350.1973 [M–H]^−^), with each corresponding to six degrees of unsaturation. Their 1D NMR data (Table 2) resembled those of **1**, but signals corresponding to the cysteine moiety were absent. Key HMBC correlations from H-3′ to C-3, C-2′, and C-1′ suggested connectivity between C-3′ and C-3 via a nitrogen atom, forming a γ-piperidine ring. An HMBC correlation from Me-7 to C-6 in **3** confirmed the presence of a methoxy group at C-6, thereby determining the planar structures of **2** and **3** (Figure 2).

**Figure 3:**
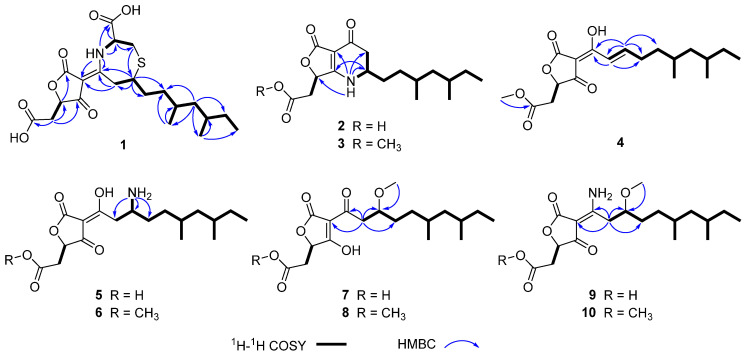
Key HMBC and ^1^H–^1^H COSY correlations of **1**–**10**.

Talactone D (**4**) was isolated as a yellow powder. Its molecular formula, C_19_H_28_O_6_, was determined by HR-ESIMS data (*m/z* 351.1798 [M–H]^−^, calcd 351.1813), indicating six degrees of unsaturation. The 1D NMR data of **4** (Table 2) were similar to those of the linear tetronate acid moiety of **1**, with the main differences being the absence of the cysteine unit and the presence of a double bond in the side chain. This was confirmed by key HMBC correlations from H-2′ to C-1′, C-3′, and C-4′ and the ^1^H–^1^H COSY correlations (Figure 3). The HMBC correlation from Me-7 to C-6 confirmed methoxylation at C-6. The *E*-configuration of the ∆^2′,3′^ double bond was assigned based on the large coupling constant (*J*_H-2′,H-3′_ =1 5.4 Hz).

Talactone E (**5**) and talactone F (**6**) were also obtained as yellow powders. Their molecular formulae were determined to be C_18_H_29_NO_6_ and C_19_H_31_NO_6_, respectively, by HR-ESI-MS. Analysis of 1D and 2D NMR data (Table 3) indicated that **5** and **6** are structurally similar to **4**. The primary difference is the presence of an amino group at the side chain in **5**, while **6** features both an amino group and an exocyclic methoxy group. Key HMBC correlations from NH_2_ to C-2′, C-3′, and C-4′ confirmed the location of the amino group at C-3′ in both compounds. An HMBC correlation from Me-7 to C-6 in **6** confirmed methoxylation at C-6.

Talactone G (**7**), talactone H (**8**), talactone I (**9**), and talactone J (**10**) were also obtained as yellow powders. Their molecular formulae, determined by HR-ESI-MS, were C_19_H_30_O_7_, C_20_H_32_O_7_, C_19_H_31_NO_6_, and C_20_H_33_NO_6_, respectively. Comparison of their NMR spectra with those of **4** indicated that **7**–**10** share the same linear tetronate scaffold (Table 3 and Table 4). The key difference is the presence of a methoxy group at C-3′ in all four compounds, as confirmed by HMBC correlations from Me-13′ to C-3′. Additionally, HMBC correlations from Me-7 to C-6 confirmed a methoxy group at C-6 in **8** and **10**. Compounds **9** and **10** exhibited an enamine group at C-1′, replacing the original carbonyl, as supported by the HR-ESI-MS data and the characteristically high-field shift of C-1′ in the ^13^C-NMR spectrum.

**Figure 4:**
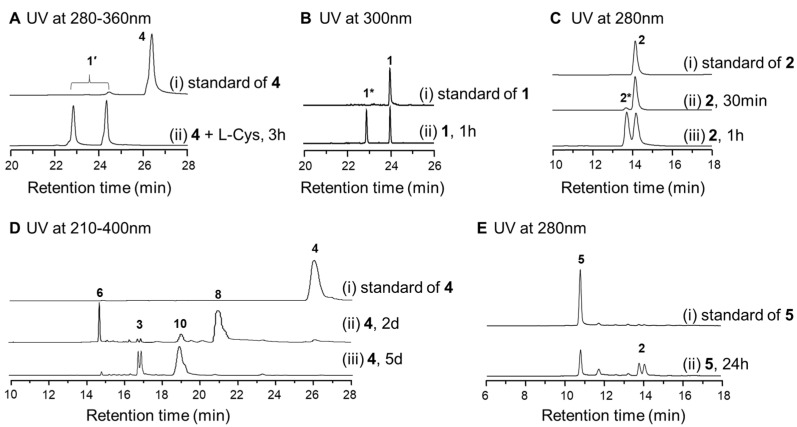
Non-enzymatic formation and interconversion of talactones. (**A**) LC-MS analysis of the reaction between compound **4** and l-cysteine in methanol after incubation at 28 °C for 24 h. (**B**,**C**) LC-MS analysis of compounds **1** and **2** in methanol, respectively, after incubation at room temperature. (**D**,**E**) LC-MS analysis of **4** and **5**, respectively, after treatment with ammonia–methanol solution (pH < 7) at room temperature.

**Scheme 1:**
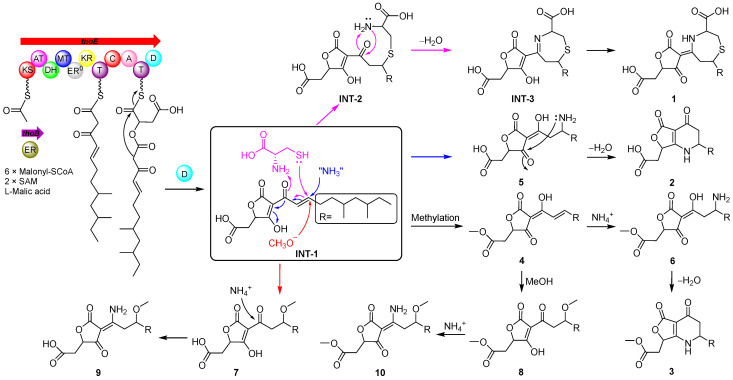
Proposed biosynthetic pathway of **1**–**10**. **INT-1/2/3**, intermediate compounds.

During separation, spontaneous transformations were observed. To assess whether **4** and l-cysteine can undergo non-enzymatic conversion to **1′** (the C-6 methyl esterification derivative of **1**), an in vitro incubation was performed. A product with a molecular weight of 455 was detected from the reaction mixture (Figure 4A). LC-MS/MS analysis showed that **1′** exhibited a deprotonated ion at *m/z* 454.1895 [M–H]^−^, with key MS/MS fragments at *m/z* 410.1999, 238.0711, and 194.0810 (Figure S78). In contrast, **1** showed a deprotonated ion at *m/z* 440.1742 [M–H]^−^ and fragments at *m/z* 396.1846, 224.0561, and 180.0661 (Figure S78). The uniform 14 Da mass shift in all fragments of **1′** confirms the addition of a methyl group without altering the core fragmentation pathway. These results suggest that the α,β-unsaturated ketone moiety of linear tetronate acid intermediate **INT-1** (the product of ThoE and ThoB) undergoes nucleophilic addition with the thiol and amino groups of L-cysteine, forming a 1,4-thiazepane ring (Scheme 1) [15]. In addition, compounds **1**/1* and **2**/2* were identified as two pairs of rapidly interconverting geometric isomers in solution (Figure 4B,C). Furthermore, compounds **4** and **5** were unstable under weakly acidic conditions. When dissolved in an ammonia–methanol solution (pH < 7), **4** converted into intermediates **6** and **8**, which were subsequently transformed into **3** and **10**, respectively (Figure 4D). Similarly, **5** was converted into **2** (Figure 4E). These acid-induced transformations are likely driven by the reactivity of the α,β-unsaturated ketone moiety in **4**. Under weakly acidic conditions, methanol and ammonia act as nucleophiles in 1,4-conjugate additions. The HOMO orbitals of methoxy and amino anions attack the LUMO orbitals at the β-carbon of the enone, leading to methoxy or amino addition at C-3′. Concurrently, increased electrophilicity of the ketone carbonyl facilitates nucleophilic addition with ammonia, forming imine or enamine derivatives (Scheme 1) [16,17]. The biosynthesis of related compounds **7** and **9** is also attributed to such non-enzymatic reactions, highlighting how environmental factors like pH and nucleophile availability contribute to structural diversification.

The antibacterial activities of all isolated compounds were evaluated against *Staphylococcus aureus* ATCC 29213, *Bacillus subtilis* ATCC 6633, *Escherichia coli* CGMCC 25922, and *Acinetobacter baumannii* ATCC 19606. The results show that some compounds exhibited significant inhibitory activity and gave a 7–18 mm clear zone of inhibition at 1 mM/disk. Compounds **1**, **2**, **3**, **9**, and **10** exhibited activity against *B. subtilis*, while **1**, **2**, and **3** were also active against *S. aureus*. Additionally, **1**, **2**, **8**, and **10** showed weak activity against *A. baumannii* (Figure S2). None of the compounds were active against *E. coli*. Analysis of the structure–activity relationships suggests that specific structural features enhance antibacterial efficacy. Compound **1**, containing a 1,4-thiazepane ring, demonstrated activity against both Gram-positive and Gram-negative strains. In contrast, compounds **2** and **3**, featuring a 2,3-dihydrofuro[3,4-*b*]pyridine-4,5(1*H*,7*H*)-dione ring, showed selective activity primarily against Gram-positive bacteria.

## 3. Materials and Methods

### 3.1. General Experimental Procedures

^1^H, ^13^C NMR, and 2D NMR spectra were recorded on a Bruker AV-600 MHz NMR spectrometer (Bruker, Karlsruhe, Germany) using tetramethylsilane (TMS) as a reference. ESI-MS and HR-ESI-MS spectroscopic data were obtained using an AmaZon SL ion trap mass spectrometer (Bruker, Karlsruhe, Germany) and a MaXis quadrupole-time-of-flight mass spectrometer (Bruker, Karlsruhe, Germany), respectively. Semi-preparative reversed-phase (SP-RP) HPLC was performed on an LC-20A preparative liquid chromatography system (Shimadzu, Tokyo, Japan) with a YMC-Pack ODS column (250 × 20 mm, 5 µm, 12 nm). Reversed-phase-medium pressure liquid chromatography (RP-MPLC) was carried out using the CHEETAH MP200 system (Bonna-Agela Technologies, Wilmington, DC, USA) and ClaricepFlash columns packed with ODS (40–63 µm, YMC).

### 3.2. Materials and Culture Conditions

The strains used in this study are listed in Table S1. *A. flavipes* LY1-5 was cultured on potato dextrose agar (PDA, BD) at 28 °C for 5–7 days. Mycelia were then transferred into potato dextrose broth (PDB, BD) for genomic DNA extraction. *Escherichia coli* XL1-Blue was used for DNA manipulation. Heterologous expression was carried out using *A. nidulans* A1145 as the host strain. *Saccharomyces cerevisiae* BJ5464-NpgA was used for plasmid construction.

### 3.3. Whole-Genome Sequencing and Bioinformatic Analysis

The whole-genome sequencing of *A. flavipes* LY1-5 was performed by Majorbio Bio-pharm Technology Co., Ltd. (Shanghai, China). The function of the coding sequence was predicted by AUGUSTUS (http://bioinf.uni-greifswald.de/webaugustus/, accessed on 5 October 2023). The natural product biosynthetic gene clusters were analyzed by antiSMASH fungal version (https://fungismash.secondarymetabolites.org, accessed on 5 October 2023). Proteins encoded by the *tho* gene cluster were further analyzed by 2ndFind (http://biosyn.nih.go.jp/2ndfind/, accessed on 5 October 2023), BlastP (https://blast.ncbi.nlm.nih.gov/Blast.cgi, accessed on 10 October 2023), and InterPro (https://www.ebi.ac.uk/interpro/, accessed on 15 October 2023).

### 3.4. Gene Cloning, Plasmid Construction, and Genetic Manipulation

The plasmids and primers used in this work are listed in Tables S2 and S3, respectively. Mycelia collected from PDB cultures were ground in liquid nitrogen with a mortar. Genomic DNA was extracted using cetyltrimethylammonium bromide (CTAB) buffer followed by phenol–chloroform purification. All *tho* genes and their native terminators (300–500 bp downstream from the stop codon) were amplified by PCR using genomic DNA as the template. Yeast fungal artificial chromosomes (YFACs) were derived from pYFAC-CH2, pYFAC-CH3, or pYFAC-CH4 (Addgene IDs #168978, #168979, #168980). YFAC constructs were assembled in *S. cerevisiae* via homologous recombination. Transform *S. cerevisiae* with the PCR fragments and *Pac* I/*Asc* I/*Asis* I/*Not* I-linearized pYFAC vectors were co-transformed into yeast using the Frozen-EZ Yeast Transformation II Kit (ZymoResearch). Transformants were selected on uracil-deficient medium or relevant auxotrophic dropout medium after 2 days of growth. Plasmids were extracted using the Zymoprep™ Yeast Plasmid Miniprep I Kit and propagated in *E. coli* XL-1 via electroporation. Constructs verified by restriction enzyme digestion and Sanger sequencing were used for *A. nidulans* transformation.

### 3.5. Transformation of A. nidulans

*A. nidulans* A1145 was used as the heterologous host. Fungal protoplast preparation and transformation were performed according to the method described by Yee and Tang [18]. *A. nidulans* transformants (Table S1) were generated via PEG-mediated protoplast transformation. Strains transformed with empty pYFAC-CH2, pYFAC-CH3, and pYFAC-CH4 vectors served as the controls. All transformants were verified by PCR.

### 3.6. Fermentation and LC/LC−MS Analysis

*A. nidulans* transformants were cultivated in liquid LMM medium (20 g/L starch, 10 g/L casein hydrolysate (acid), 50 mL/L nitrate salts, 1 mL/L trace elements, 20 g/L agar) at 37 °C with shaking at 200 rpm for 2 days. Cyclopentanone was then added to induce expression, and the temperature was reduced to 25 °C until the end of fermentation. Cultures were extracted three times with ethyl acetate (EtOAc). The combined organic phases were evaporated to dryness and re-dissolved in 200 μL of MeOH/H_2_O (90:10, *v*/*v*). The extracts were analyzed by high-performance liquid chromatography–photodiode array detection (HPLC-DAD) on a Thermo C18 column (2.1 mm × 100 mm, 0.5 μm, 0.3 mL/min) at a flow rate of 0.3 mL/min, using a linear gradient of 5–100% acetonitrile (CH_3_CN) in water (0.1% trifluoroacetic acid) over 20 min, followed by 5 min of isocratic 100% CH_3_CN.

### 3.7. Extraction, Isolation, and Purification

Compounds **1**–**10** were isolated from the transformant strain *A. nidulans*::*thoBE*. A large-scale liquid fermentation culture (10 L LMM medium) was extracted four times with EtOAc. The crude extract (3 g) was subjected to a C18 column using a stepwise gradient of MeOH/H_2_O, yielding eight subfractions (Fr.1–Fr.8, 20% to 100%). Fr.2 was purified by RP-HPLC (YMC-Pack ODS-A column, 250 × 10 mm, 5 µm) with 40% CH_3_CN-H_2_O at 2.5 mL/min to afford compounds **5** (2.1 mg, *t*_R_ = 20.1 min) and **7** (3.0 mg, *t*_R_ = 29.8 min). Fr.4 was purified with 50% CH_3_CN-H_2_O to yield **2** (3.3 mg, *t*_R_ = 23.0 min), **8** (3.5 mg, *t*_R_ = 28.4 min), and **9** (2.6 mg, *t*_R_ = 35.7 min). Fr.6 was purified using a gradient of 50–70% CH_3_CN-H_2_O to give **6** (1.7 mg, *t*_R_ = 25.1 min), **3** (1.3 mg, *t*_R_ = 30.2 min), and **10** (2.0 mg, *t*_R_ = 39.4 min). Fr.7 was eluted with a gradient of 60–85% CH_3_CN-H_2_O to afford **1** (3.7 mg, *t*_R_ = 18.5 min) and **4** (5.7 mg, *t*_R_ = 24.9 min).

Talactone A (**1**): Yellow powder; UV (CH_3_OH) *λ*_max_ (log *ε*) 228 (2.84) and 300 (3.75) nm; ^1^H and ^13^C NMR data, see Table 1 and Figures S4–S14; HR-ESI-MS *m*/*z* 440.1737 [M−H]^−^ (calcd. C_21_H_30_NO_8_S^−^, 440.1748).

Talactone B (**2**): Yellow powder; UV (CH_3_OH) *λ*_max_ (log *ε*) 228 (2.50) and 284 (3.84) nm; ^1^H and ^13^C NMR data, see Table 2 and Figures S16–S21; HR-ESI-MS *m*/*z* 336.1807 [M−H]^−^ (calcd. C_18_H_26_NO_5_^−^, 336.1816).

Talactone C (**3**): Yellow powder; UV (CH_3_OH) *λ*_max_ (log *ε*) 225 (2.48) and 281 (3.81) nm; ^1^H and ^13^C NMR data, see Table 2 and Figures S23–S28; HR-ESI-MS *m*/*z* 350.1973 [M−H]^−^ (calcd. C_19_H_28_NO_5_^−^, 350.1973).

Talactone D (**4**): Yellow powder; UV (CH_3_OH) *λ*_max_ (log *ε*) 218 (1.75), 248 (1.36), and 316 (3.82) nm; ^1^H and ^13^C NMR data, see Table 3 and Figures S30–S35; HR-ESI-MS *m*/*z* 351.1798 [M−H]^−^ (calcd. C_19_H_27_O_6_^−^, 351.1813).

Talactone E (**5**): Yellow powder; UV (CH_3_OH) *λ*_max_ (log *ε*) 232 (2.33) and 268 (2.45) nm; ^1^H and ^13^C NMR data, see Table 3 and Figures S37–S42; HR-ESI-MS *m*/*z* 354.1904 [M−H]^−^ (calcd. C_18_H_28_NO_6_^−^, 354.1922).

Talactone F (**6**): Yellow powder; UV (CH_3_OH) *λ*_max_ (log *ε*) 230 (2.36) and 266 (2.51) nm; ^1^H and ^13^C NMR data, see Table 3 and Figures S44–S49; HR-ESI-MS *m*/*z* 368.2068 [M−H]^−^ (calcd. C_19_H_30_NO_6_^−^, 368.2078).

Talactone G (**7**): Yellow powder; UV (CH_3_OH) *λ*_max_ (log *ε*) 208 (1.74), 232 (1.85), and 268 (2.75) nm; ^1^H and ^13^C NMR data, see Table 4 and Figures S51–S56; HR-ESI-MS *m*/*z* 369.1906 [M−H]^−^ (calcd. C_19_H_29_O_7_^−^, 369.1919).

Talactone H (**8**): Yellow powder; UV (CH_3_OH) *λ*_max_ (log *ε*) 205 (1.57), 230 (1.64), and 266 (2.84) nm; ^1^H and ^13^C NMR data, see Table 4 and Figures S58–S63; HR-ESI-MS *m*/*z* 368.2068 [M−H]^−^ (calcd. C_20_H_31_O_7_^−^, 368.2078).

Talactone I (**9**): Yellow powder; UV (CH_3_OH) *λ*_max_ (log *ε*) 224 (3.29) and 288 (3.75) nm; ^1^H and ^13^C NMR data, see Table 4 and Figures S65–S70; HR-ESI-MS *m*/*z* 368.2062 [M−H]^−^ (calcd. C_19_H_30_NO_6_^−^, 368.2079).

Talactone J (**10**): Yellow powder; UV (CH_3_OH) *λ*_max_ (log *ε*) 227 (3.10) and 292 (3.46) nm; ^1^H and ^13^C NMR data, see Table 4 and Figures S72–S77; HR-ESI-MS *m*/*z* 382.2237 [M−H]^−^ (calcd. C_20_H_32_NO_6_^−^, 382.2235).

### 3.8. Antibacterial Activity Assay

Antibacterial activities of isolated compounds were evaluated using the Bauer–Kirby disk diffusion method. Inhibition zones were measured against two Gram-positive bacteria (*S. aureus* ATCC 29213 and *B. subtilis* ATCC 6633) and two Gram-negative bacteria (*E. coli* CGMCC 25922 and *A. baumannii* ATCC 19606). Bacteria culture (10 μL) was mixed with 30 mL of melted LB agar (~40 °C) and solidified in Petri dishes. Sterile filter paper discs (5 mm diameter) were loaded with 10 μL of each compound (1 mM). Discs containing DMSO served as the blank control. After drying for 10 min, the discs were placed on the agar surface and incubated at 37 °C for 16 h. The results are identified by a clear zone of inhibition, and all experiments were performed in triplicate.

## 4. Conclusions and Future Prospects

In conclusion, heterologous expression of the *tho* gene cluster from *A. flavipes* in *A. nidulans* enabled the discovery of ten new tetronate natural products, talactones A–J (**1**–**10**). Structural elucidation revealed remarkable chemical diversity, most notably the rare 1,4-thiazepane scaffold in talactone A (**1**) and the novel 2,3-dihydrofuro[3,4-*b*]pyridine-4,5(1*H*,7*H*)-dione skeleton in talactones B (**2**) and C (**3**). Biosynthetic investigations provided key mechanistic insights, demonstrating that the formation of these complex scaffolds involves essential non-enzymatic steps. Specifically, the 1,4-thiazepane ring in **1** results from a spontaneous reaction between a tetronate acid and l-cysteine, whereas the 2,3-dihydrofuro[3,4-*b*]pyridine-4,5(1*H*,7*H*)-dione cores of **2** and **3** are formed via spontaneous intramolecular cyclization of respective precursors (**5** and **6**) under acidic conditions. These findings underscore the important role of non-enzymatic processes, including intramolecular cyclizations and multicomponent reactions, in expanding structural diversity following enzymatic assembly. Furthermore, the antibacterial activities exhibited by compounds **1**–**3**, **9**, and **10** highlight the utility of heterologous expression strategy for discovering novel bioactive molecules with potential therapeutic relevance.

Notably, the current heterologous expression system suffers from low yields of talactones, which limits further structural modification and in-depth biological activity evaluation. To address this issue, future efforts should focus on the optimization of expression and fermentation conditions. On the one hand, host strain engineering could be performed: for example, knocking out genes involved in the catabolism of tetronate precursors in *A. nidulans*, overexpressing key regulatory factors that promote the transcription of the *tho* gene cluster, or introducing auxiliary genes to enhance the supply of endogenous substrates (e.g., acetyl-CoA, malonyl-CoA) required for tetronate biosynthesis. On the other hand, fermentation process optimization is equally critical, including screening of optimal carbon sources (e.g., glucose, sucrose, maltose) and nitrogen sources (e.g., peptone, yeast extract, ammonium salts); adjusting culture parameters such as pH, temperature, aeration rate, and induction time; as well as adding exogenous precursors (e.g., l-cysteine) or signal molecules to stimulate the production of talactones.

Beyond the demonstrated antibacterial activity, tetronate natural products represented by talactones hold considerable potential for developing novel therapeutics targeting other diseases, which merits in-depth exploration. For example, anti-tumor activity is a promising direction: Many natural products with cyclic scaffolds similar to tetronates have been reported to inhibit tumor cell proliferation, induce apoptosis, or suppress angiogenesis by targeting key signaling pathways (e.g., PI3K/Akt, MAPK) or enzymes (e.g., topoisomerase, histone deacetylase) [19]. The unique 1,4-thiazepane and 2,3-dihydrofuro[3,4-b]pyridine-4,5(1*H*,7*H*)-dione scaffolds in talactones A–C (**1**–**3**) may endow them with specific binding affinity to tumor-related targets, making them potential lead compounds for anti-tumor drug development.

## Figures and Tables

**Table 1:** ^1^H and ^13^C NMR spectroscopic data of compound **1**.

No.	1 *^a^*		1 *^b^*	
	*δ*_C_, Type	*δ*_H_ (*J* in Hz)	*δ*_C_, Type	*δ*_H_ (*J* in Hz)
1	173.7, C		170.8, C	
2	94.4, C		91.8, C	
3	198.8, C		197.0, C	
4	80.4, CH	4.82, m	78.4, CH	4.76, m
5	36.7, CH_2_	2.94, td, (16.7, 3.9) 2.68, dd, (16.7, 7.3)	36.1, CH_2_	2.80, m 2.56, dd, (17.6, 7.6)
6	172.7, C		171.0, C	
1′	171.0, C		170.8, C	
2′	20.2, CH_2_	4.31, dd, (15.6, 7.3) 3.58, dd, (20.5, 15.6)	34.9, CH_2_	4.14, dd, (14.3, 7.3) 3.37, m
3′	54.9 CH	3.20, m	38.0, CH	3.03, m
4′	25.2, CH_2_	1.40, m	31.0, CH_2_	1.37, m
5′	35.2, CH_2_	1.47, m 1.31, m	28.7, CH_2_	1.30, m 1.02, m
6′	31.0, CH	1.52, m	31.1, CH	1.35, m
7′	45.3, CH_2_	1.21, m 0.92, m	43.9, CH_2_	1.16, m 0.86, m
8′	32.8, CH	1.41, m	29.4, CH	1.61, m
9′	30.3, CH_2_	1.32, m 1.09, m	34.3, CH_2_	1.47, m 1.06, m
10′	11.5, CH_3_	0.86, t, (overlapped)	11.0, CH_3_	0.82, t, (overlapped)
11′	20.2, CH_3_	0.83, d, (overlapped)	20.2, CH_3_	0.81, d, (overlapped)
12′	20.0, CH_3_	0.83, d, (overlapped)	19.6, CH_3_	0.81, d, (overlapped)
1″	171.4, C		170.3, C	
2″	48.0, CH	5.35, dd, (15.3, 10.0)	60.0, CH	5.08, m
3″	44.8, CH_2_	3.48, dd, (15.3, 5.2) 3.12, m	28.0, CH_2_	3.03, m
NH				11.54, d, (5.4)

*^a^* Recorded in CD_3_OD (150 and 600 MHz). *^b^* Recorded in DMSO-*d*_6_ (150 and 600 MHz).

**Table 2:** ^1^H and ^13^C NMR spectroscopic data of compounds **2**–**4**.

No.	2 *^a^*		3 *^a^*		4 *^b^*	
	*δ*_c_, Type	*δ*_H_ (*J* in Hz)	*δ*_C_, Type	*δ*_H_ (*J* in Hz)	*δ*_C_, type	*δ*_H_ (*J* in Hz)
1	166.3, C		166.2, C		170.6, C	
2	93.4, C		93.4, C		97.4, C	
3	176.3, C		175.5, C		183.2, C	
4	72.1, CH	5.21, m	71.6, CH	5.27, m	79.3, CH	4.92, m
5	37.9, CH_2_	3.00, d, (16.0) 2.63, dd, (16.0, 7.9)	36.9, CH_2_	3.14, m 2.78, m	35.9, CH_2_	2.98, d, (4.2) 2.86, d, (6.4)
6	170.7, C		169.5, C		170.6, C	
7			51.9, CH_3_	3.61, s	52.7, CH_3_	3.65, s
1′	185.4, C		185.3, C		178.0, C	
2′	41.6, CH_2_	2.45, m 2.23, m	41.2, CH_2_	2.42, m 2.20, m	121.0, CH	7.11, d, (15.4)
3′	54.2, CH	3.83, m	54.2, CH	3.80, m	156.2, CH	7.41, m
4′	30.5f, CH_2_	1.58, m	30.5, CH_2_	1.56, m	31.7, CH_2_	2.43, m
5′	32.2, CH_2_	1.33, m 1.11, m	31.8, CH_2_	1.36, m 1.07, m	35.5, CH_2_	1.55, m 1.29, m
6′	29.7, CH	1.48, m	31.1, CH	1.39, m	30.6, CH	1.56, m
7′	44.0, CH_2_	1.18, m 0.89, m	44.0, CH_2_	1.19, m 0.90, m	45.0, CH_2_	1.27, m 0.97, m
8′	31.1, CH	1.42, m	29.7, CH	1.45, m	32.3, CH_2_	1.34, m
9′	28.7, CH_2_	1.23, m 1.05, m	28.7, CH_2_	1.29, m 1.02, m	29.8, CH_2_	1.37, m
10′	11.4, CH_3_	0.81, (overlapped)	11.0, CH_3_	0.82, (overlapped)	11.0, CH_3_	0.82, (overlapped)
11′	20.1, CH_3_	0.78, (overlapped)	20.1, CH_3_	0.81, (overlapped)	20.2, CH_3_	0.81, (overlapped)
12′	19.7, CH_3_	0.78, (overlapped)	19.7, CH_3_	0.81, (overlapped)	20.0, CH_3_	0.81, (overlapped)
NH				9.58, d, (16.5)		

*^a^* Recorded in DMSO-*d*_6_ (150 and 600 MHz). *^b^* Recorded in CD_3_CN (150 and 600 MHz).

**Table 3:** ^1^H and ^13^C NMR spectroscopic data of compounds **5**–**7**.

No.	5 *^a^*		6 *^a^*		7 *^a^*	
	*δ*_C_, Type	*δ*_H_ (*J* in Hz)	*δ*_C_, Type	*δ*_H_ (*J* in Hz)	*δ*_C_, type	*δ*_H_ (*J* in Hz)
1	173.8, C		173.6, C		177.8, C	
2	94.9, C		94.6, C		97.5, C	
3	194.5, C		193.8, C		197.8, C	
4	76.7, CH	4.44, m	75.9, CH	4.41, m	79.4, CH	4.64, m
5	38.1, CH_2_	2.65, d, (15.5) 2.18, m	37.1, CH_2_	2.76, dt, (16.0, 3.1) 2.32, dd, (16.0, 7.4)	40.1, CH_2_	2.79, m 2.28, dd, (16.0, 9.5)
6	172.4, C		171.0, C		176.3, C	
7			51.7, CH_3_	3.61, s		
1′	190.3, C		190.0, C		195.7, C	
2′	40.9, CH_2_	3.12, m 2.81, m	41.2, CH_2_	3.09, dt (17.3, 4.3) 2.80, dd (17.3, 8.2)	45.8, CH_2_	3.07, m 2.79, m
3′	48.3, CH	3.37, m	48.3, CH	3.37, m	79.6, CH	3.69, m
4′	29.8, CH_2_	1.52, m	29.8, CH_2_	1.50, m	32.6, CH_2_	1.38, m
5′	31.6, CH_2_	1.34, m 1.03, m	28.6, CH_2_	1.29, m 1.03, m	31.5, CH_2_	1.45, m
6′	31.0, CH	1.40, m	29.5, CH	1.42, m	30.3, CH	1.43, m
7′	43.9, CH_2_	1.17, m 0.87, m	43.8, CH_2_	1.18, m 0.89, m	45.9, CH_2_	1.20, m 0.86, m
8′	29.6, CH	1.39, m	30.9, CH	1.40, m	32.9, CH	1.51, m
9′	28.7, CH_2_	1.27, m 1.03, m	31.6, CH_2_	1.30, m 1.07, m	30.6, CH_2_	1.30, m 1.05, m
10′	11.1, CH_3_	0.83, (overlapped)	11.0, CH_3_	0.82, (overlapped)	11.6, CH_3_	0.83, (overlapped)
11′	20.6, CH_3_	0.81, (overlapped)	20.6, CH_3_	0.80, (overlapped)	20.7, CH_3_	0.81, (overlapped)
12′	20.1, CH_3_	0.81, (overlapped)	20.1, CH_3_	0.80, (overlapped)	20.2, CH_3_	0.0.81, (overlapped)
13′					56.9, CH_3_	3.28, s
NH_2_				7.67, s		

*^a^* Recorded in DMSO-*d*_6_ (150 and 600 MHz).

**Table 4:** ^1^H and ^13^C NMR spectroscopic data of compounds **8**–**10**.

No.	8 *^a^*		9 *^b^*		10 *^b^*	
	*δ*_C_, Type	*δ*_H_ (*J* in Hz)	*δ*_C_, Type	*δ*_H_ (*J* in Hz)	*δ*_C_, type	*δ*_H_ (*J* in Hz)
1	176.8, C		171.4, C		171.0, C	
2	96.9, C		90.5, C		90.3, C	
3	196.0, C		197.5, C		197.0, C	
4	77.4, CH	4.56, m	77.1, CH	4.66, m	76.6, CH	4.71, m
5	37.6, CH_2_	2.83, dd, (16.4, 3.6) 2.49, dd, (16.4, 8.8)	36.5, CH_2_	2.77, td, (16.7, 3.9) 2.50, dd, (16.7, 7.3)	36.0, CH_2_	2.86, m 2.63, m
6	172.3, C		171.4, C		170.2, C	
7	52.6, CH_3_	3.63, s			51.8, CH_3_	3.58, s
1′	195.9, C		172.7, C		172.7, C	
2′	45.4, CH_2_	2.86, m	36.8, CH_2_	2.89, m	37.0, CH_2_	2.93, m
3′	79.7, CH	3.62, m	78.9, CH	3.51, m	79.1, CH	3.50, m
4′	31.9, CH_2_	1.51, m	30.8, CH_2_	1.45, m	30.8, CH_2_	1.43, m
5′	32.7, CH_2_	1.33, m 1.07, m	29.1, CH_2_	1.20, m	31.7, CH_2_	1.28, m 1.03, m
6′	31.0, CH	1.48, m	29.8, CH	1.47, m	29.8, CH	1.43, m
7′	45.4, CH_2_	1.22, m 0.91, m	44.4, CH_2_	1.15, m 0.86, m	44.4, CH_2_	1.17, m 0.87, m
8′	32.4, CH	1.42, m	31.4, CH	1.35, m	31.3, CH	1.36, m
9′	30.0, CH_2_	1.37, m	31.7, CH_2_	1.26, m 1.07, m	28.9, CH_2_	1.28, m 1.03, m
10′	11.5, CH_3_	0.83, (overlapped)	11.3, CH_3_	0.81, (overlapped)	11.2, CH_3_	0.78, (overlapped)
11′	20.6, CH_3_	0.81, (overlapped)	20.4, CH_3_	0.78, (overlapped)	20.4, CH_3_	0.78, (overlapped)
12′	20.1, CH_3_	0.81, (overlapped)	19.9, CH_3_	0.78, (overlapped)	19.8, CH_3_	0.78, (overlapped)
13′	56.5, CH_3_	3.21, s	56.6, CH_3_	3.20, s	56.6, CH_3_	3.22, s

*^a^* Recorded in CD_3_CN (150 and 600 MHz). *^b^* Recorded in DMSO-*d*_6_ (150 and 600 MHz).

## Data Availability

The data presented in this study are available in this article and the Supplementary Materials.

## Supplementary Materials

The following supporting information can be downloaded at: https://www.mdpi.com/article/10.3390/jof12010028/s1: Table S1. Strains used in the study; Table S2. Plasmids used in the study; Table S3. Primers used in the study; Figure S1. Schematic diagram of plasmids constructed for heterologous expression; Figure S2. Antimicrobial activity evaluation of isolated compounds; Figures S3–S14. The HR-ESI-MS and NMR spectra of **1**; Figures S15–S21. The HR-ESI-MS and NMR spectra of **2**; Figures S22–S28. The HR-ESI-MS and NMR spectra of **3**; Figures S29–S35. The HR-ESI-MS and NMR spectra of **4**; Figures S36–S42. The HR-ESI-MS and NMR spectra of **5**; Figures S43–S49. The HR-ESI-MS and NMR spectra of **6**; Figures S50–S56. The HR-ESI-MS and NMR spectra of **7**; Figures S57–S63. The HR-ESI-MS and NMR spectra of **8**; Figures S64–S70. The HR-ESI-MS and NMR spectra of **9**; Figures S71–S77. The HR-ESI-MS and NMR spectra of **10**; Figure S78. The LC-MS/MS spectrum of compounds **1** and **1′**.
