# Supplementary material for: Genome Mining and Heterologous Reconstitution of a PKS-NRPS Gene Cluster from Aspergillus flavipes LY1-5 Affords Structurally Novel Tetronates
Source: J Fungi (Basel). 2025 Dec 29;12(1):28. doi: 10.3390/jof12010028 (PMC12843353; doi:10.3390/jof12010028)
Supplement: Supplementary file 1 [file jof-12-00028-s001.zip › jof-4044451-supplementary.pdf]

Supporting Information

Genome mining and heterologous reconstitution of a PKS-NRPS gene cluster from *Aspergillus flavipes* LY1-5 affords structurally novel tetronates

Quan Dai [1], Yiqiao Li [1], Shuzhe Lv [1], Shuang Zhao [1], Liyuan Han [1], Jiaxin Xu [1], Hui Shuai [4,5], Youming Zhang [1,3*] and Fu Yan [1, 2*]

[1] State Key Laboratory of Microbial Technology, Shandong University, Qingdao, Shandong 266237, China

[2] Hunan Provincial Key Laboratory of Microbial Molecular Biology, College of Life Science, Hunan Normal University, Changsha, Hunan 410081, China

[3] Shenzhen Key Laboratory of Genome Manipulation and Biosynthesis, Key Laboratory of Quantitative Synthetic Biology, Shenzhen Institute of Synthetic Biology, Shenzhen Institutes of Advanced Technology, Chinese Academy of Sciences, Shenzhen, Guangdong 518055, China.

[4] State Key Laboratory of Integration and Innovation of Classic Formula and Modern Chinese Medicine, Lunan Pharmaceutical Group Co. Ltd., Linyi, Shandong 276005, China

[5] Rizhao Marine Biotechnology Center, Rizhao Polytechnic, Rizhao, Shandong 276826, China

* Correspondence: zhangyouming@sdu.edu.cn (Y.Z.) and fuyan@sdu.edu.cn (F.Y.)

Table of contents

Table S1. Strains used in the study. 4
Table S2. Plasmids used in the study. 4
Table S3. Primers used in the study 5
Figure S1. Schematic diagram of plasmids constructed for heterologous expression. 6
Figure S2. Antibacterial activity evaluation of isolated compounds. 6
Figure S3. The HRESIMS spectrum of **1**. 7
Figure S4. ^{1}H NMR of **1** in CD_3OD (600 MHz). 7
Figure S5. ^{13}C NMR of **1** in CD_3OD (150 MHz). 8
Figure S6. HSQC of **1** in CD_3OD (600 and 150 MHz). 8
Figure S7. HMBC of **1** in CD_3OD (600 and 150 MHz). 9
Figure S8. ^{1}H-^{1}H COSY of **1** in CD_3OD (600 MHz). 9
Figure S9. NOESY of **1** in CD_3OD (600 MHz). 10
Figure S10. ^{1}H NMR of **1** in DMSO-d_6 (600 MHz). 10
Figure S11. ^{13}C NMR of **1** in DMSO-d_6 (150 MHz). 11
Figure S12. HSQC of **1** in DMSO-d_6 (600 and 150 MHz). 11
Figure S13. HMBC of **1** in DMSO-d_6 (600 and 150 MHz). 12
Figure S14. ^{1}H-^{1}H COSY of **1** in DMSO-d_6 (600 MHz). 12
Figure S15. The HRESIMS spectrum of **2** 13
Figure S16. ^{1}H NMR of **2** in DMSO-d_6 (600 MHz). 13
Figure S17. ^{13}C NMR of **2** in DMSO-d_6 (150 MHz). 14
Figure S18. HSQC of **2** in DMSO-d_6 (600 and 150 MHz). 14
Figure S19. HMBC of **2** in DMSO-d_6 (600 and 150 MHz). 15
Figure S20. ^{1}H-^{1}H COSY of **2** in DMSO-d_6 (600 MHz). 15
Figure S21. NOESY of **2** in DMSO-d_6 (600 MHz). 16
Figure S22. The HRESIMS spectrum of **3** 16
Figure S23. ^{1}H NMR of **3** in DMSO-d_6 (600 MHz). 17
Figure S24. ^{13}C NMR of **3** in DMSO-d_6 (150 MHz). 17
Figure S25. HSQC of **3** in DMSO-d_6 (600 and 150 MHz). 18
Figure S26. HMBC of **3** in DMSO-d_6 (600 and 150 MHz). 18
Figure S27. ^{1}H-^{1}H COSY of **3** in DMSO-d_6 (600 MHz). 19
Figure S28. NOESY of **3** in DMSO-d_6 (600 MHz). 19
Figure S29. The HRESIMS spectrum of **4** 20
Figure S30. ^{1}H NMR of **4** in CD_3CN (600 MHz). 20
Figure S31. ^{13}C NMR of **4** in CD_3CN (150 MHz). 21
Figure S32. HSQC of **4** in CD_3CN (600 and 150 MHz). 21
Figure S33. HMBC of **4** in CD_3CN (600 and 150 MHz). 22
Figure S34. ^{1}H-^{1}H COSY of **4** in CD_3CN (600 MHz). 22
Figure S35. NOESY of **4** in CD_3CN (600 MHz). 23
Figure S36. The HRESIMS spectrum of **5**. 23
Figure S37. ^{1}H NMR of **5** in DMSO-d_6 (600 MHz). 24
Figure S38. ^{13}C NMR of **5** in DMSO-d_6 (150 MHz). 24
Figure S39. HSQC of **5** in DMSO-d_6 (600 and 150 MHz). 25

Figure S40. HMBC of **5** in DMSO-d_6 (600 and 150 MHz). 25
Figure S41. ^{1}H-^{1}H COSY of **5** in DMSO-d_6 (600 MHz). 26
Figure S42. NOESY of **5** in DMSO-d_6 (600 MHz). 26
Figure S43. The HRESIMS spectrum of **6** 27
Figure S44. ^{1}H NMR of **6** in DMSO-d_6 (600 MHz). 27
Figure S45. ^{13}C NMR of **6** in DMSO-d_6 (150 MHz). 28
Figure S46. HSQC of **6** in DMSO-d_6 (600 and 150 MHz). 28
Figure S47. HMBC of **6** in DMSO-d_6 (600 and 150 MHz). 29
Figure S48. ^{1}H-^{1}H COSY of **6** in DMSO-d_6 (600 MHz). 29
Figure S49. NOESY of **6** in DMSO-d_6 (600 MHz). 30
Figure S50. The HRESIMS spectrum of **7** 30
Figure S51. ^{1}H NMR of **7** in DMSO-d_6 (600 MHz). 31
Figure S52. ^{13}C NMR of **7** in DMSO-d_6 (150 MHz). 31
Figure S53. HSQC of **7** in DMSO-d_6 (600 and 150 MHz). 32
Figure S54. HMBC of **7** in DMSO-d_6 (600 and 150 MHz). 32
Figure S55. ^{1}H-^{1}H COSY of **7** in DMSO-d_6 (600 MHz). 33
Figure S56. NOESY of **7** in DMSO-d_6 (600 MHz). 33
Figure S57. The HRESIMS spectrum of **8** 34
Figure S58. ^{1}H NMR of **8** in CD_3CN (600 MHz). 34
Figure S59. ^{13}C NMR of **8** in CD_3CN (150 MHz). 35
Figure S60. HSQC of **8** in CD_3CN (600 MHz). 35
Figure S61. HMBC of **8** in CD_3CN (600 and 150 MHz). 36
Figure S62. ^{1}H-^{1}H COSY of **8** in CD_3CN (600 MHz). 36
Figure S63. NOESY of **8** in CD_3CN (600 MHz). 37
Figure S64. The HRESIMS spectrum of **9** 37
Figure S65. ^{1}H NMR of **9** in DMSO-d_6 (600 MHz). 38
Figure S66. ^{13}C NMR of **9** in DMSO-d_6 (150 MHz). 38
Figure S67. HSQC of **9** in DMSO-d_6 (600 and 150 MHz). 39
Figure S68. HMBC of **9** in DMSO-d_6 (600 and 150 MHz). 39
Figure S69. ^{1}H-^{1}H COSY of **9** in DMSO-d_6 (600 MHz). 40
Figure S70. NOESY of **9** in DMSO-d_6 (600 MHz). 40
Figure S71. The HRESIMS spectrum of **10** 41
Figure S72. ^{1}H NMR of **10** in DMSO-d_6 (600 MHz). 41
Figure S73. ^{13}C NMR of **10** in DMSO-d_6 (150 MHz). 42
Figure S74. HSQC of **10** in DMSO-d_6 (600 and 150 MHz). 42
Figure S75. HMBC of **10** in DMSO-d_6 (600 and 150 MHz). 43
Figure S76. ^{1}H-^{1}H COSY of **10** in DMSO-d_6 (600 MHz). 43
Figure S77. NOESY of **10** in DMSO-d_6 (600 MHz). 44
Figure S78. LC-MS/MS spectrum of compounds **1** (A) and **1′** (B). 44

Table S1. Strains used in the study.

Strains	Description
A. flavipes LY1-5	Wild type strain isolated from a soil sample collected from Cretaceous National Geological Park in Laiyang, Shandong Province, China (120.649464E, 36.881212N)
E. coli XL1-blue	*recA1 endA1 gyrA96 thi-1 hsdR17supE44 relA1 lac [F' pro AB lac IqZΔM15 Tn10 (Tetr)*]
A. nidulans A1145	*pyrG89; pyroA4; nkuA::argB; riboB2;* purchased from Fungal Genetics Stock Center
A. nidulans A1145::empty vectors	*Aspergillus nidulans* A1145 harboring pYFAC-CH2, pYFAC-CH3, and pYFAC-CH4 (purchased from Addgene)
S. cerevisiae BJ5464-NpgA	Host for plasmid assembly via homologous recombination
A. nidulans::*thoE*	*A. nidulans* harboring plasmid pYFAC-AF1.
A. nidulans::*thoEB*	*A. nidulans* harboring plasmids pYFAC-AF1, pYFAC-AF2.
A.nidulans::*thoABCDEGHI*	*A. nidulans* harboring plasmids pYFAC-AF1, pYFAC-AF3, pYFAC-AF4.

Table S2. Plasmids used in the study.

Plasmids	Description
pYFAC-AF1	pYFAC-CH2 carrying *thoE* under *PalcA* promoter.
pYFAC-AF2	pYFAC-CH3 carrying *thoB* under *PalcS* promoter.
pYFAC-AF3	pYFAC-CH3 carrying *thoA, thoB, thoD,* and *thoH* driven by *PalcA, PalcS, PalcM,* and *PaldA* promoter, respectively.
pYFAC-AF4	pYFAC-CH4 carrying *thoC, thoG,* and *thoI* driven by *PalcA, PalcM,* and *PaldA* promoter, respectively.

Table S3. Primers used in the study

Name	Sequence (5′→3′)
thoE-PalcA-F	gttaattagaactcttccaatcctatcacctcgccttaatatgtcctctccgagcctcg
thoE-R	gactcgtgaggcatacgcag
thoE-F	ggactgtcaggtcaaatcggc
thoE-T1-R	tcgcgctccacggggactcgcttcaatttgttccgcttactaccgccgtgattcatgtc
thoA-PalcA-F	aattagaactcttccaatcctatcacctcgccttaatatgatgacgacagatgatcccg
thoA-T1-R	tcgcgctccacggggactcgcttcaatttgttccgcttacgacccaattgctgacttgc
thoB-PalcS/M-F	aaaggtctacaatcaattcaggccgtattcagggcggccgctacacgtgttcgttccac
thoB-T1-R	cctttgagataccaaagcattgagcccagaaacagcagaagcatgccatctgcctgccc
thoD-PalcS/M-F	ccttttccttattaaaccatatatctccagcagagcgatatgatggacgacgaccatcc
thoD-T2-R	taaagccaacttcgatctcaactaaattatctcctcccgcgctagtgggcagcggcaag
thoH-PaldA-F	gagcggcggatgcctacttacttctgggaacgaggttaatctctaacaaggcccagttc
thoH-T2-R	tctcctctcccaagatacccatatttcccgctcacggcgcgatgcttctgcgtcctctc
thoC-PalcA-F	ttagaactcttccaatcctatcacctcgccttaatatgcatgttcttattgttggaggg
thoC-T1-R	cgcgctccacggggactcgcttcaatttgttccgcttatcattgtggaacgaacacgtg
thoG-PalcS/M-F	ttcccttttccttattaaaccatatatctccagcagagcgatatggcatccctcgcctc
thoG-T2-R	aagccaacttcgatctcaactaaattatctcctcccgcgttaagcctggaacgagctgg
thoI-PaldA-F	gggagcggcggatgcctacttacttctgggaacgaggggcctctatgtcttcacacagg
thoI-T2-R	tctcctctcccaagatacccatatttcccgctcacggcgcgatgtcgtacaccctgcgc

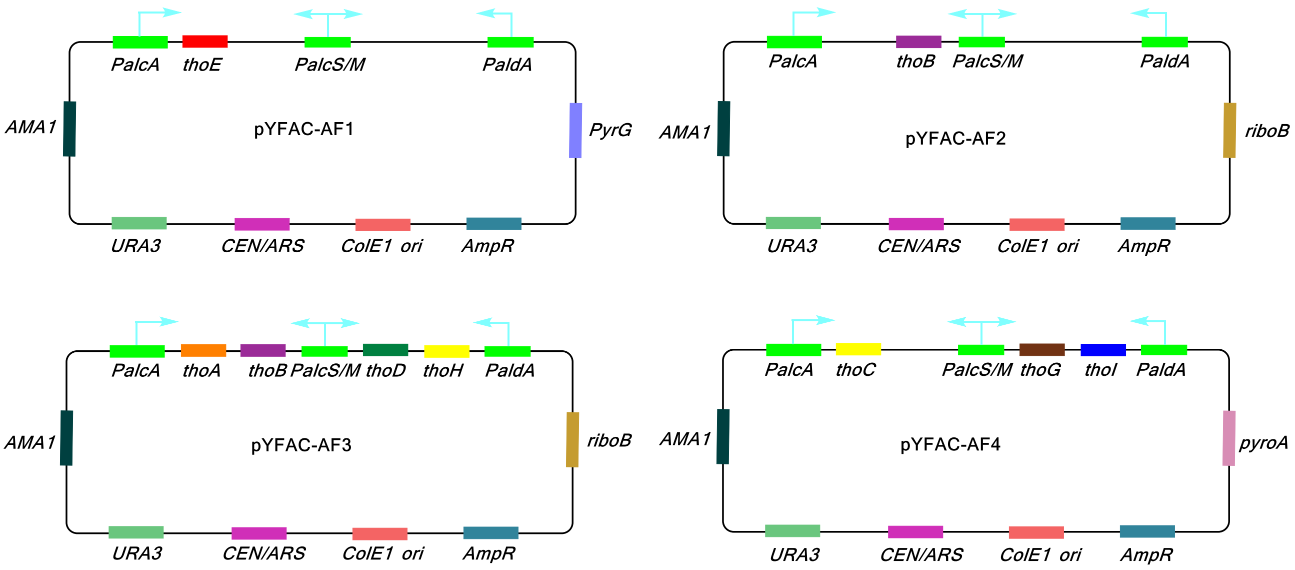

Figure S1. Schematic diagram of plasmids constructed for heterologous expression.

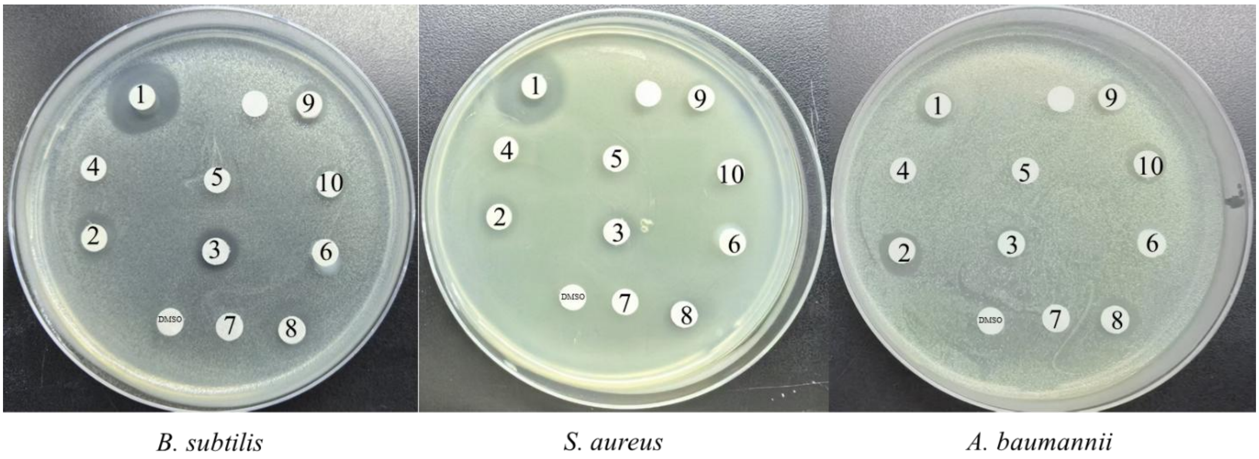

Figure S2. Antibacterial activity evaluation of isolated compounds.

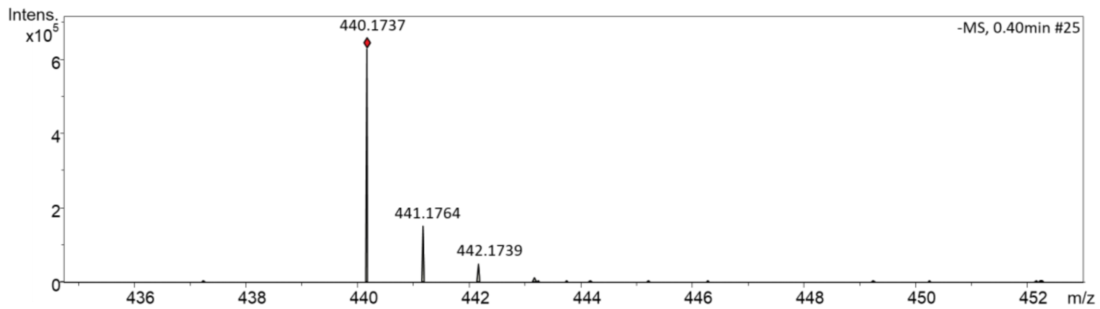

Figure S3. The HRESIMS spectrum of **1**.

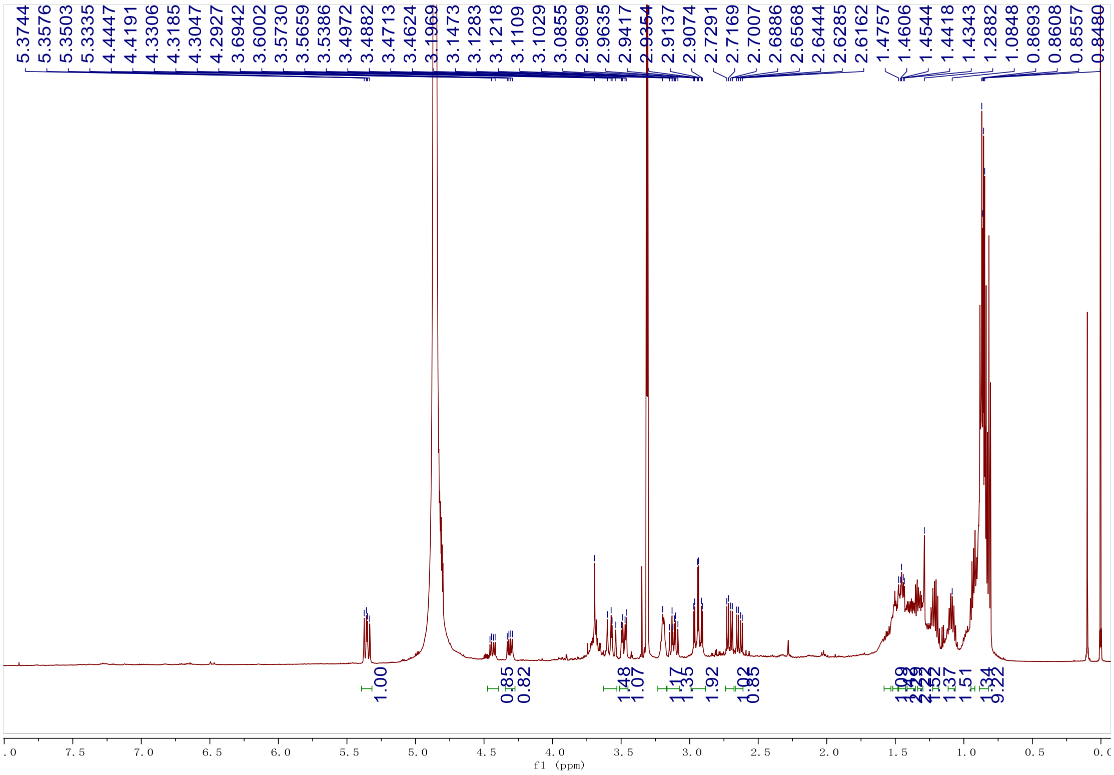

Figure S4. ^{1}H NMR of **1** in CD_3OD (600 MHz).

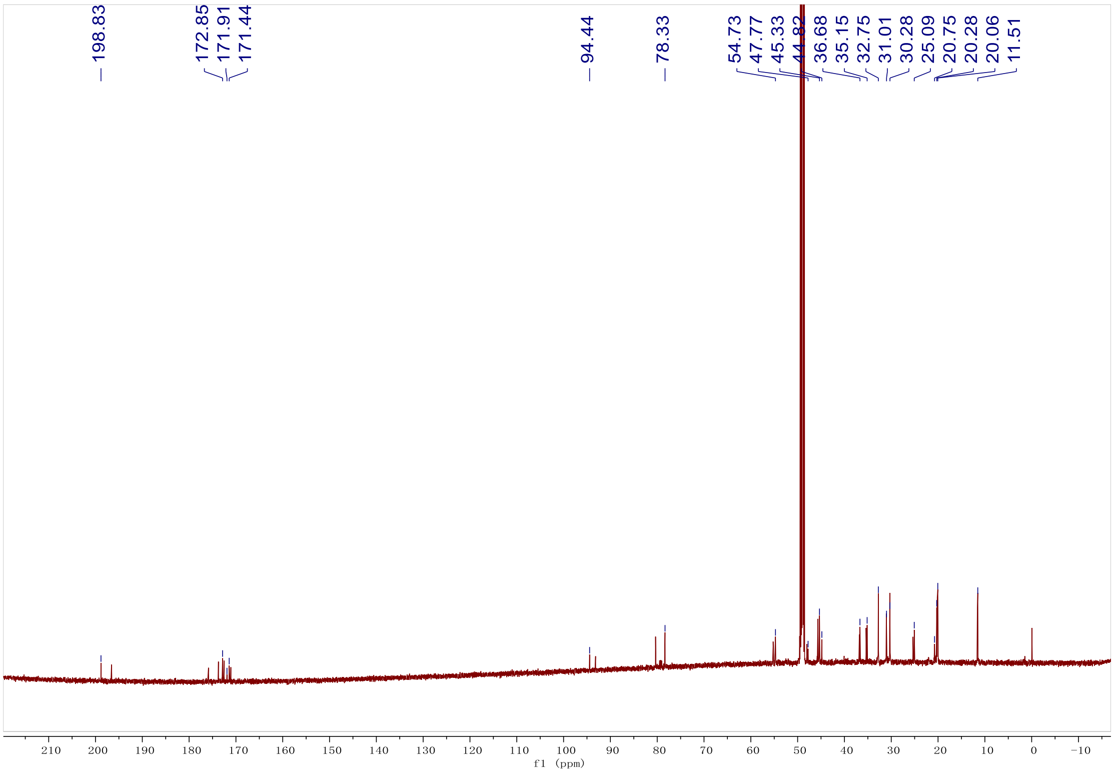

Figure S5. ^{13}C NMR of **1** in CD_3OD (150 MHz).

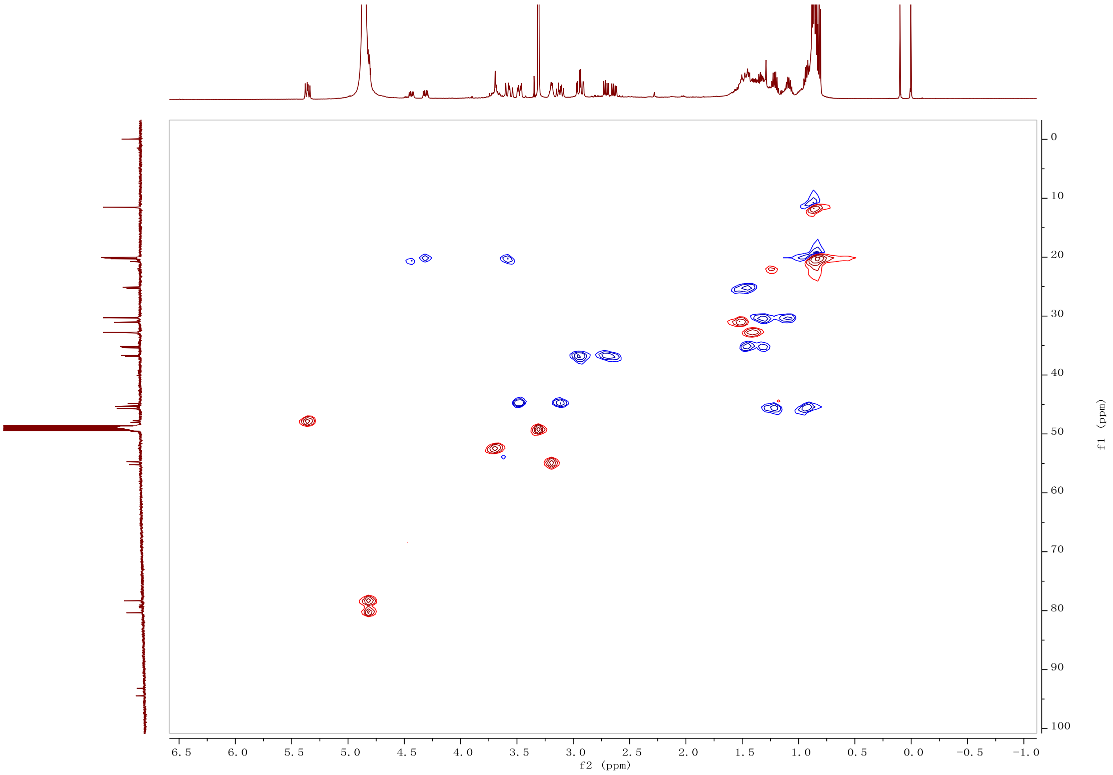

Figure S6. HSQC of **1** in CD_3OD (600 and 150 MHz).

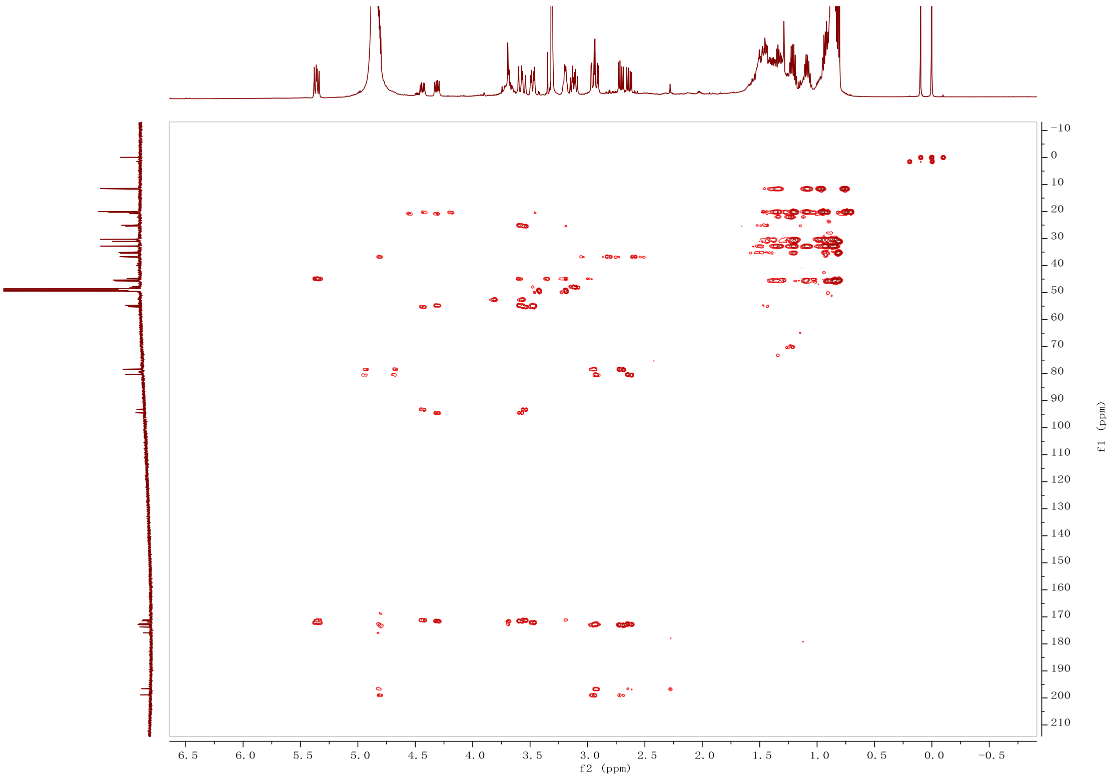

Figure S7. HMBC of **1** in CD_3OD (600 and 150 MHz).

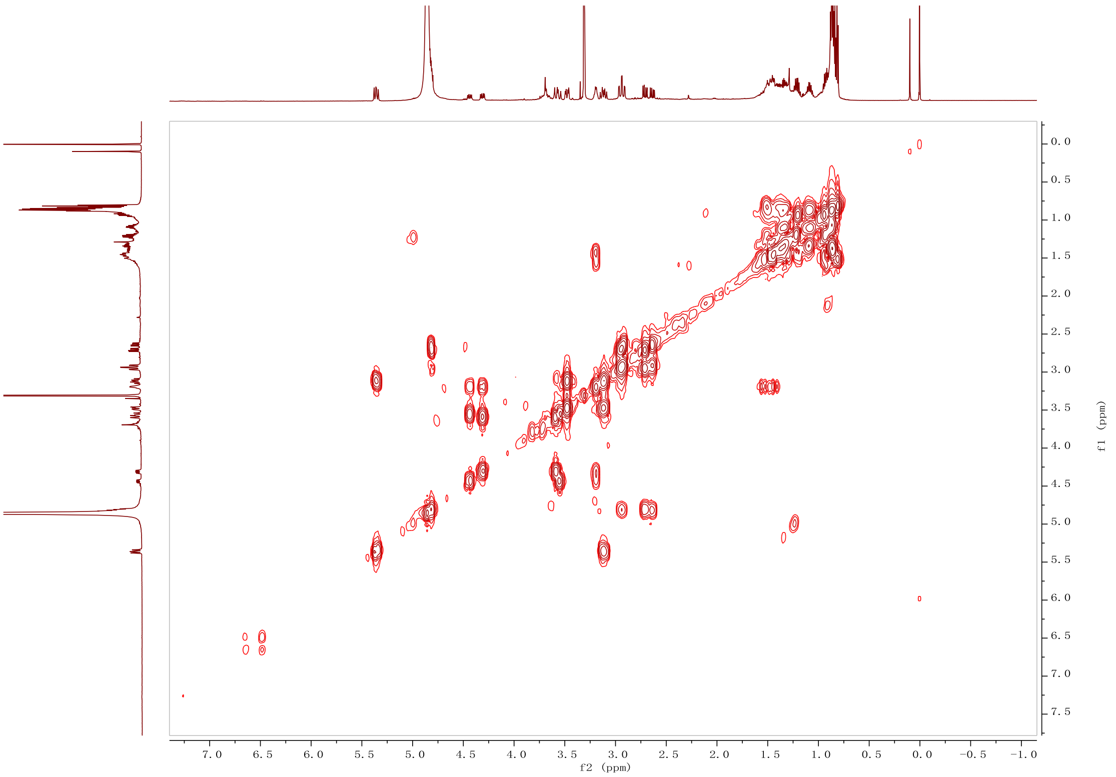

Figure S8. ^1H-^1H COSY of **1** in CD_3OD (600 MHz).

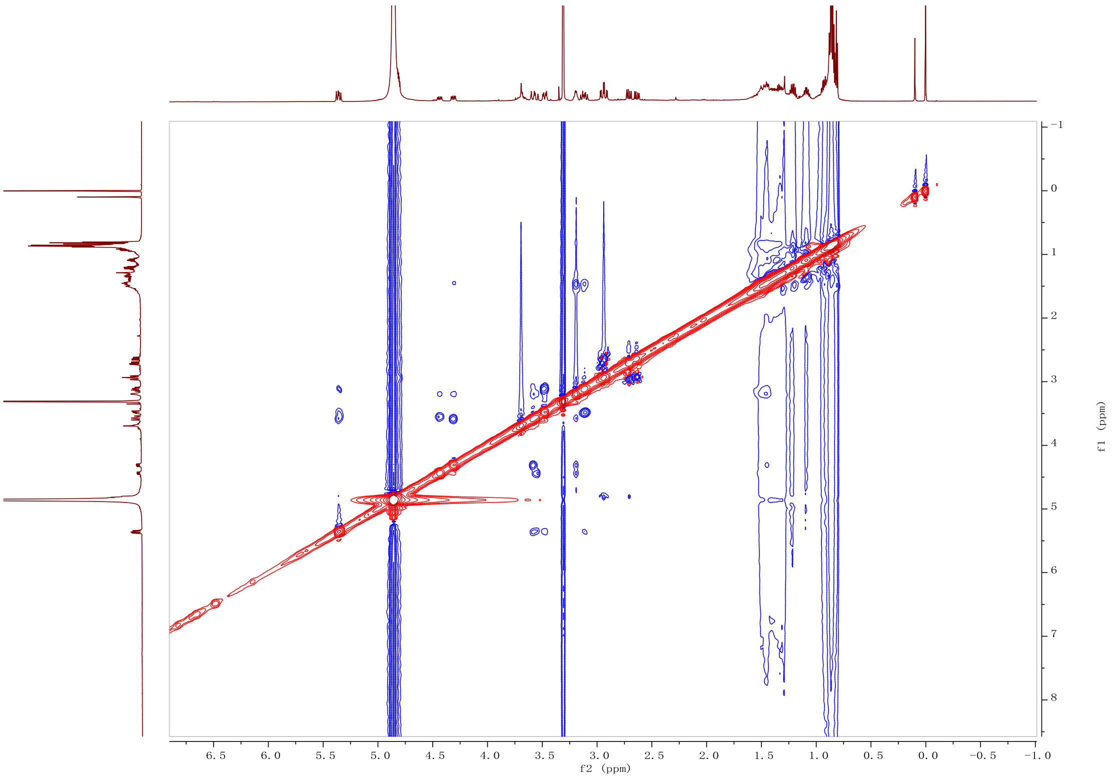

Figure S9. NOESY of **1** in CD_3OD (600 MHz).

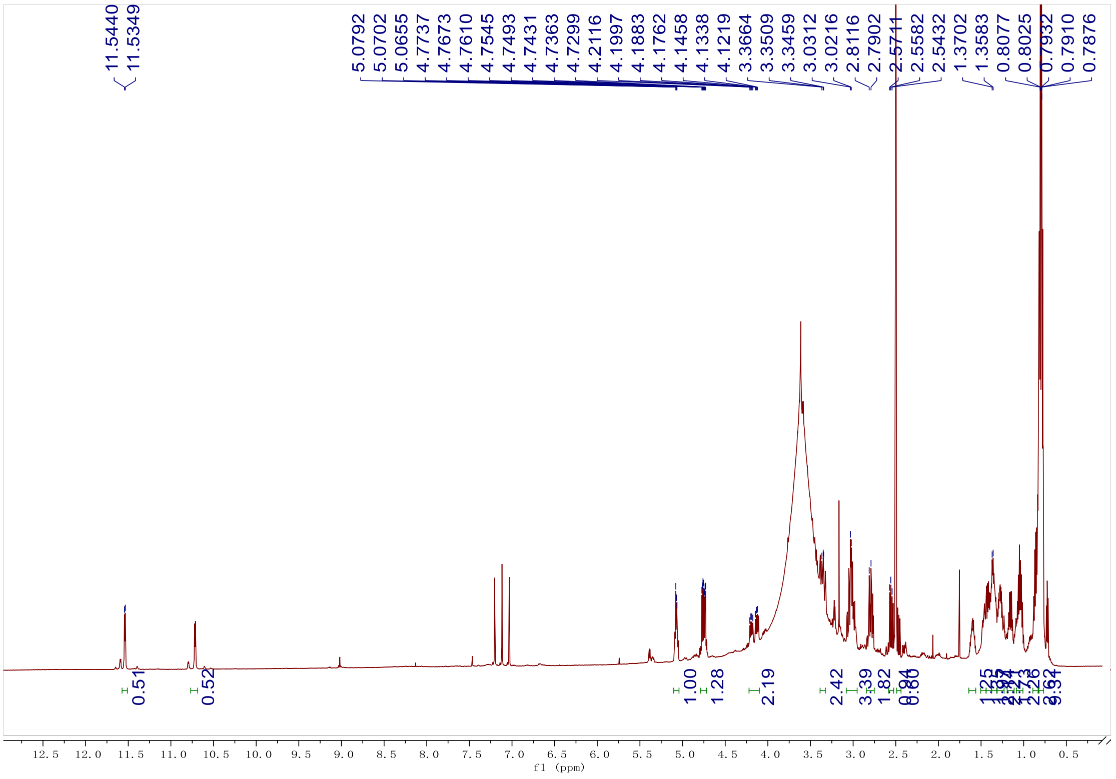

Figure S10. ^{1}H NMR of **1** in DMSO-d_6 (600 MHz).

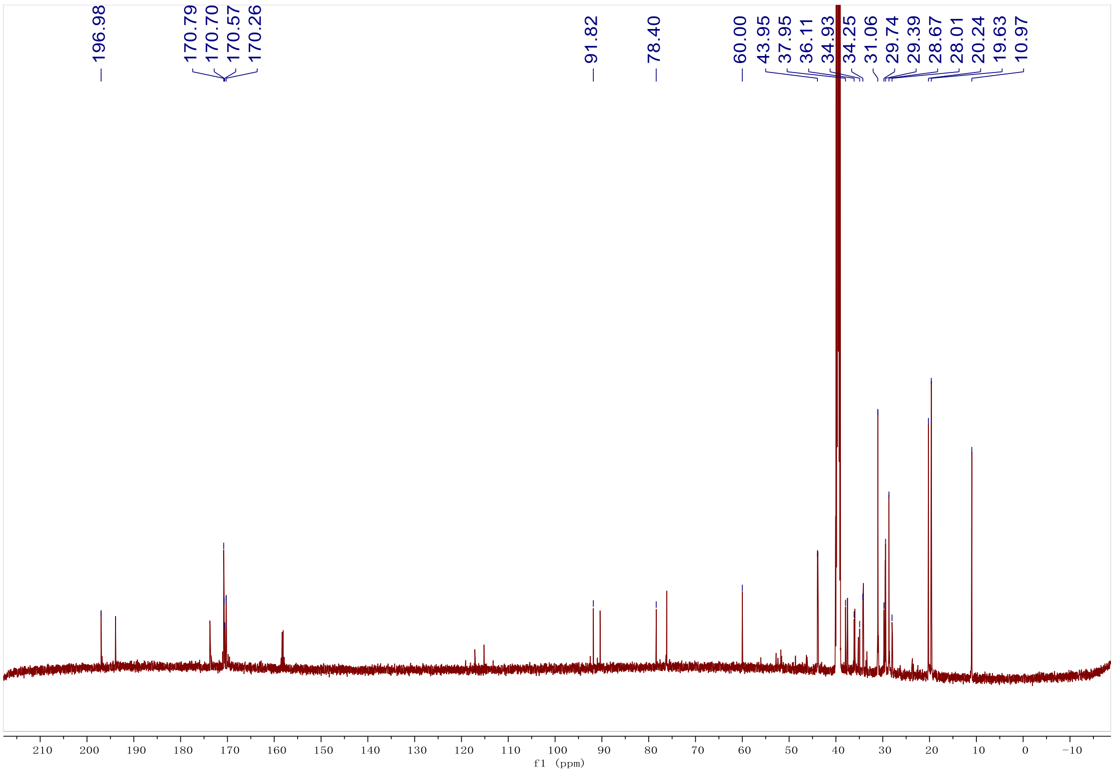

Figure S11. ^{13}C NMR of **1** in DMSO-d_6 (150 MHz).

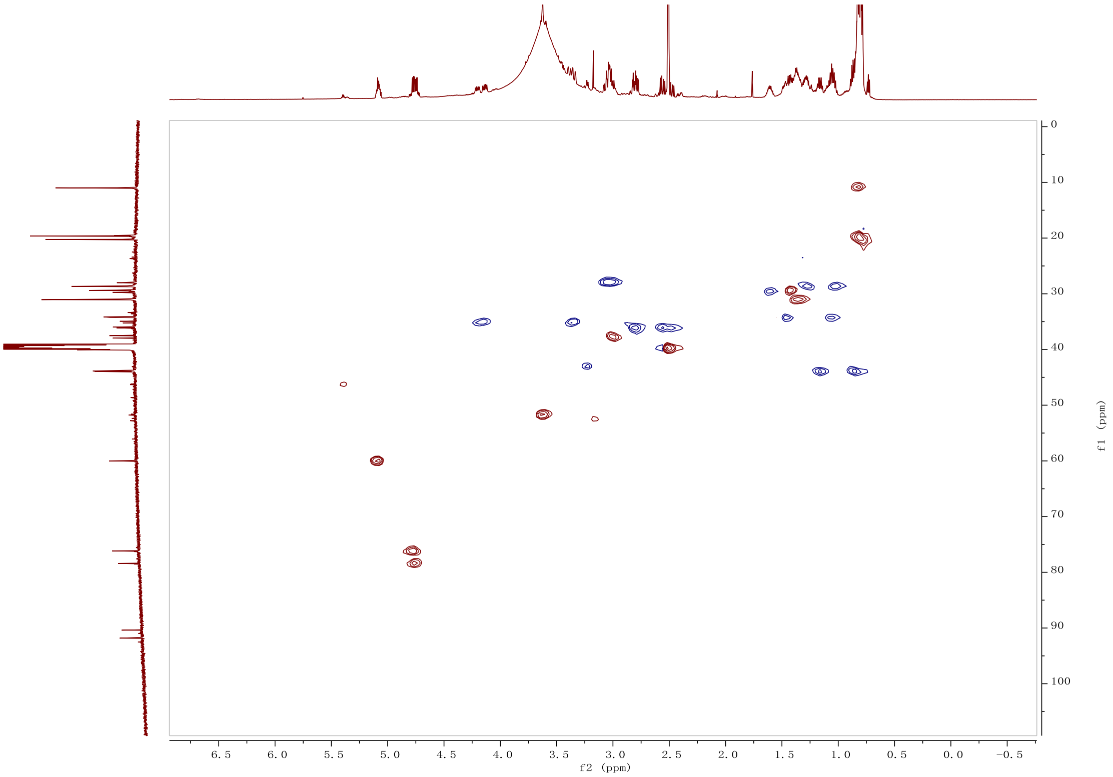

Figure S12. HSQC of **1** in DMSO-d_6 (600 and 150 MHz).

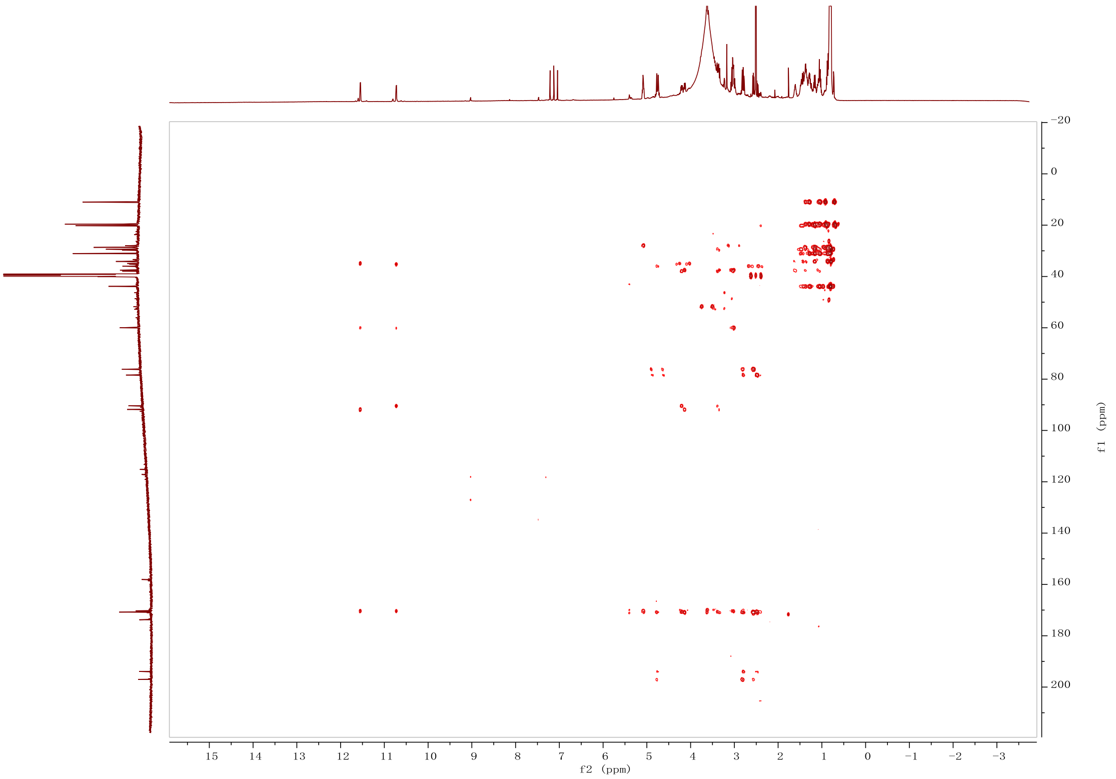

Figure S13. HMBC of **1** in DMSO-d_6 (600 and 150 MHz).

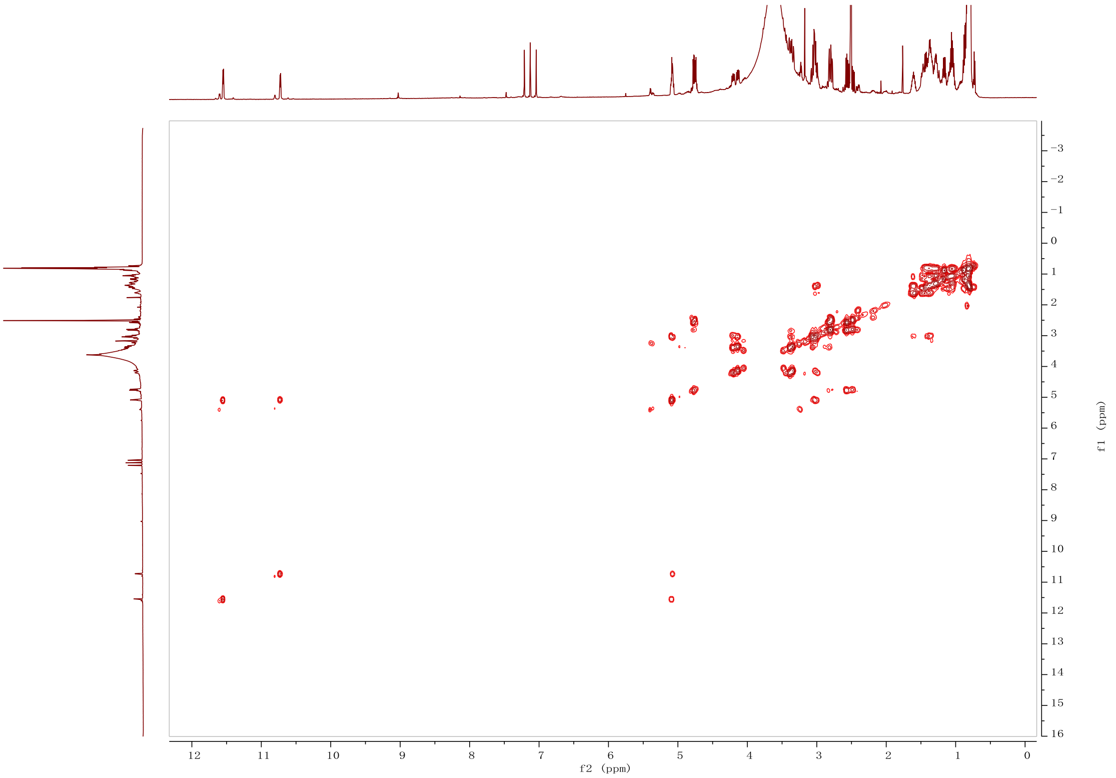

Figure S14. ^{1}H-^{1}H COSY of **1** in DMSO-d_6 (600 MHz).

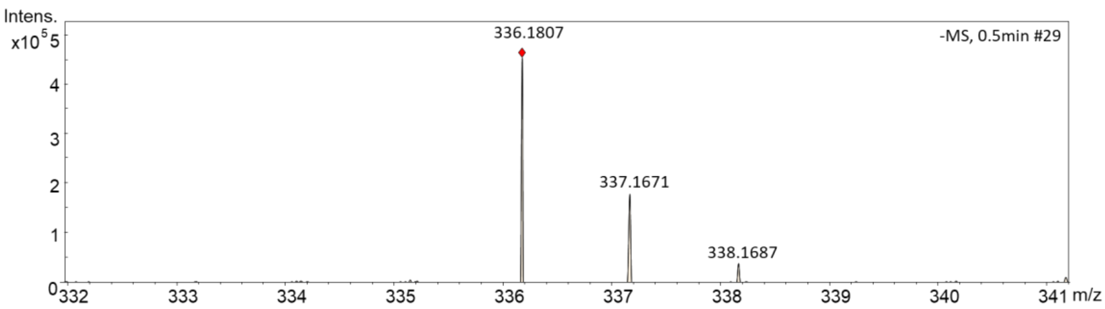

Figure S15. The HRESIMS spectrum of **2**

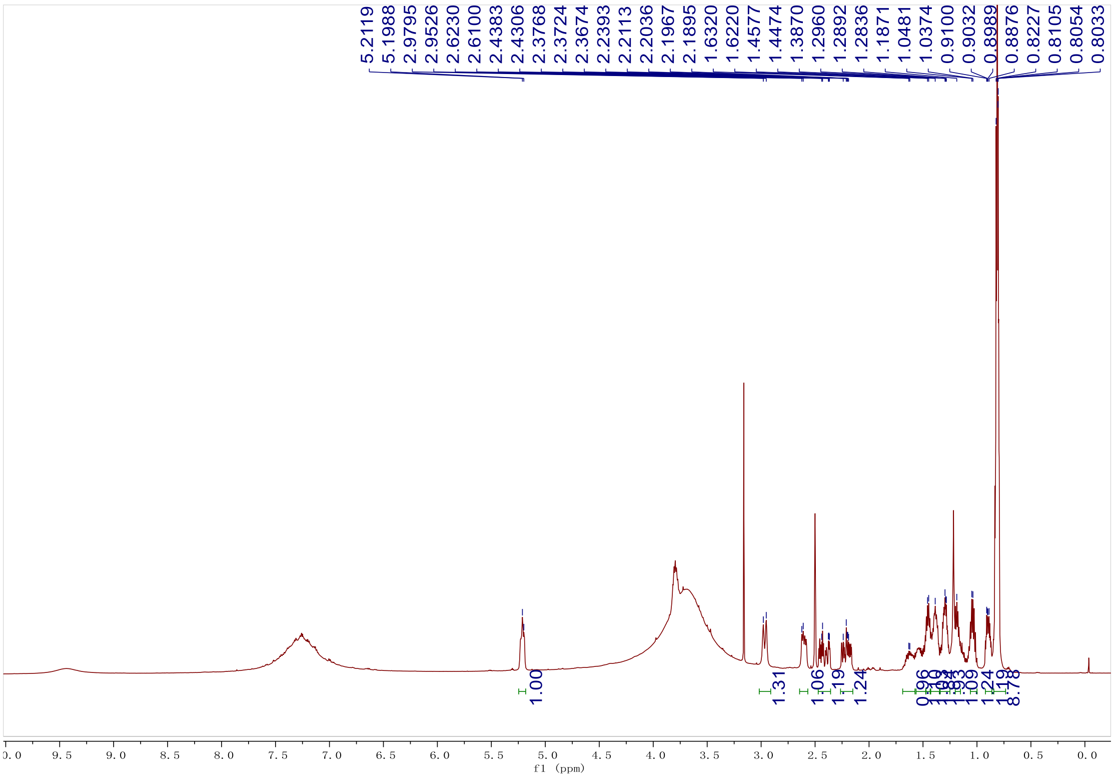

Figure S16. ^{1}H NMR of **2** in DMSO-d_6 (600 MHz).

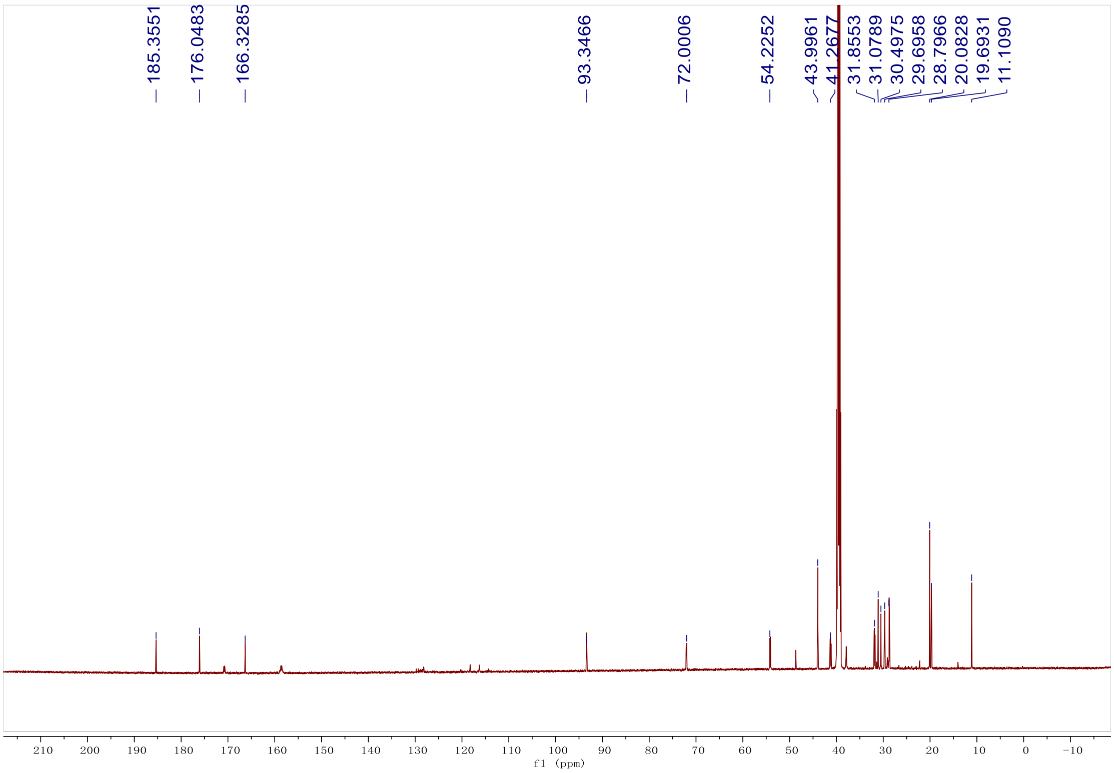

Figure S17. ^{13}C NMR of **2** in DMSO-d_6 (150 MHz).

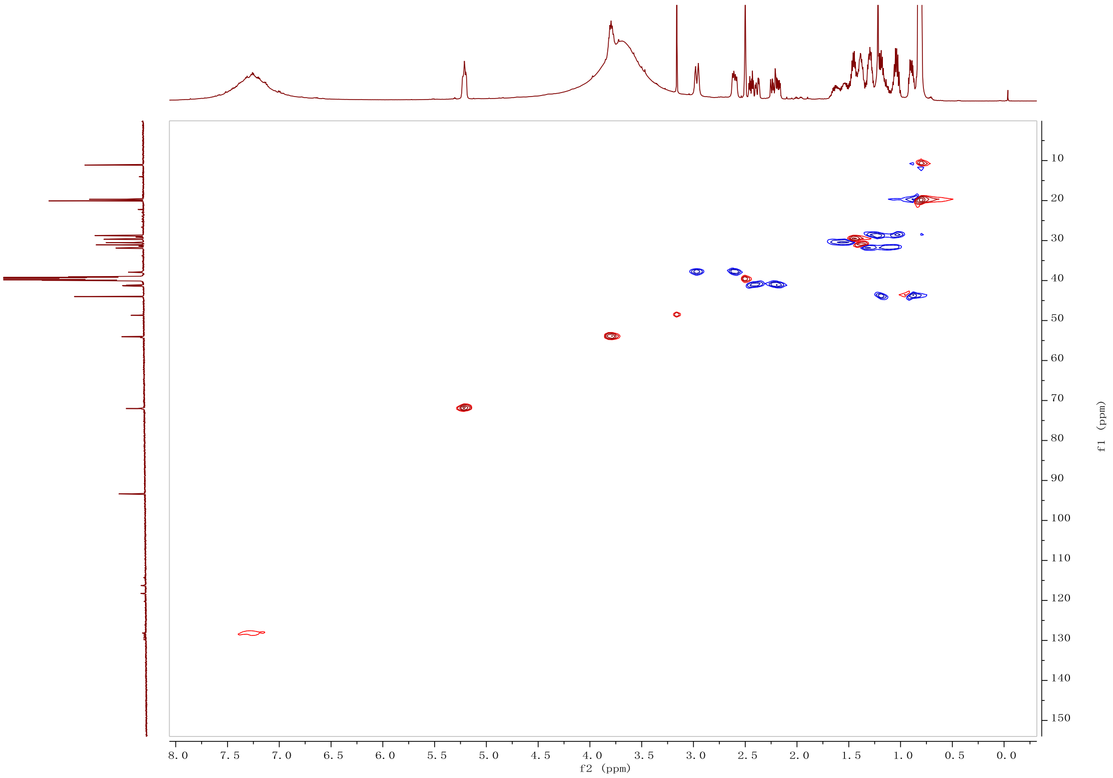

Figure S18. HSQC of **2** in DMSO-d_6 (600 and 150 MHz).

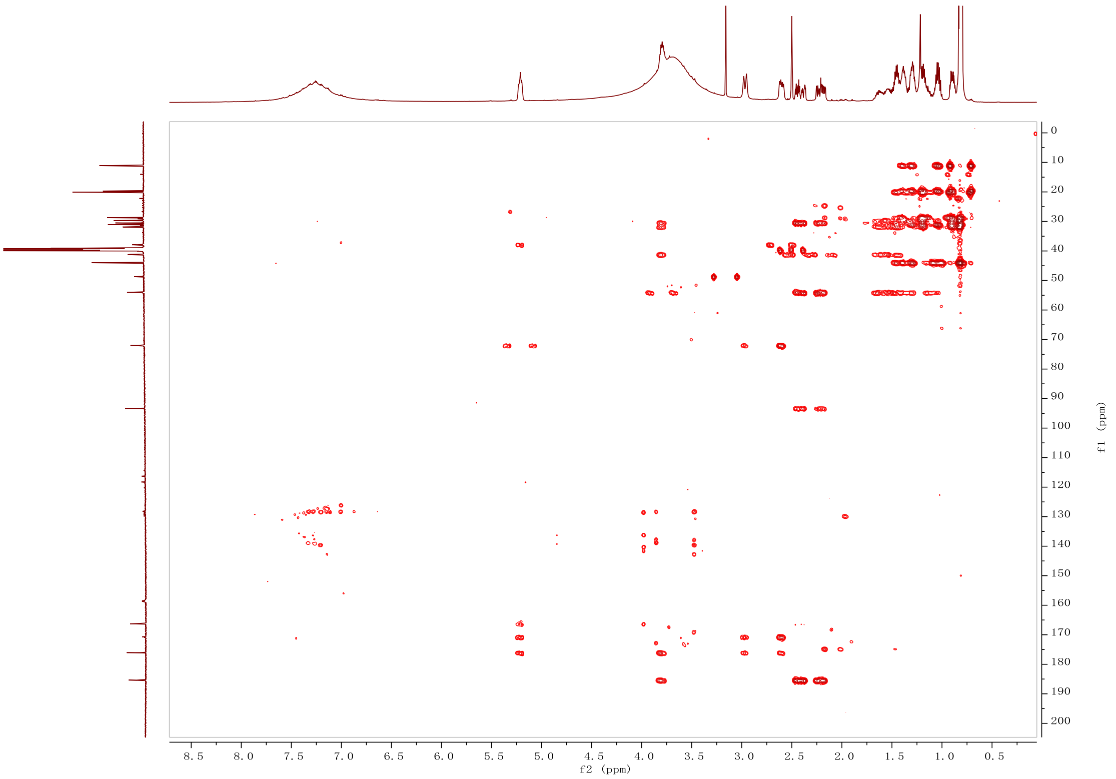

Figure S19. HMBC of **2** in DMSO-d_6 (600 and 150 MHz).

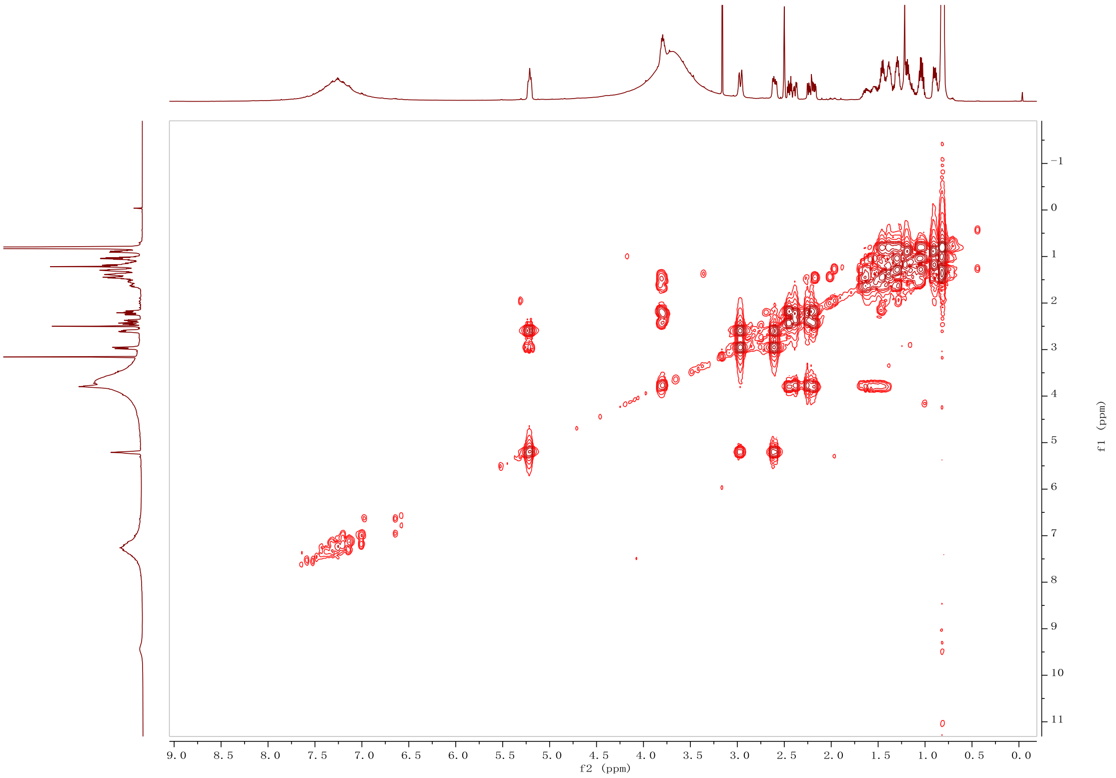

Figure S20. ^{1}H-^{1}H COSY of **2** in DMSO-d_6 (600 MHz).

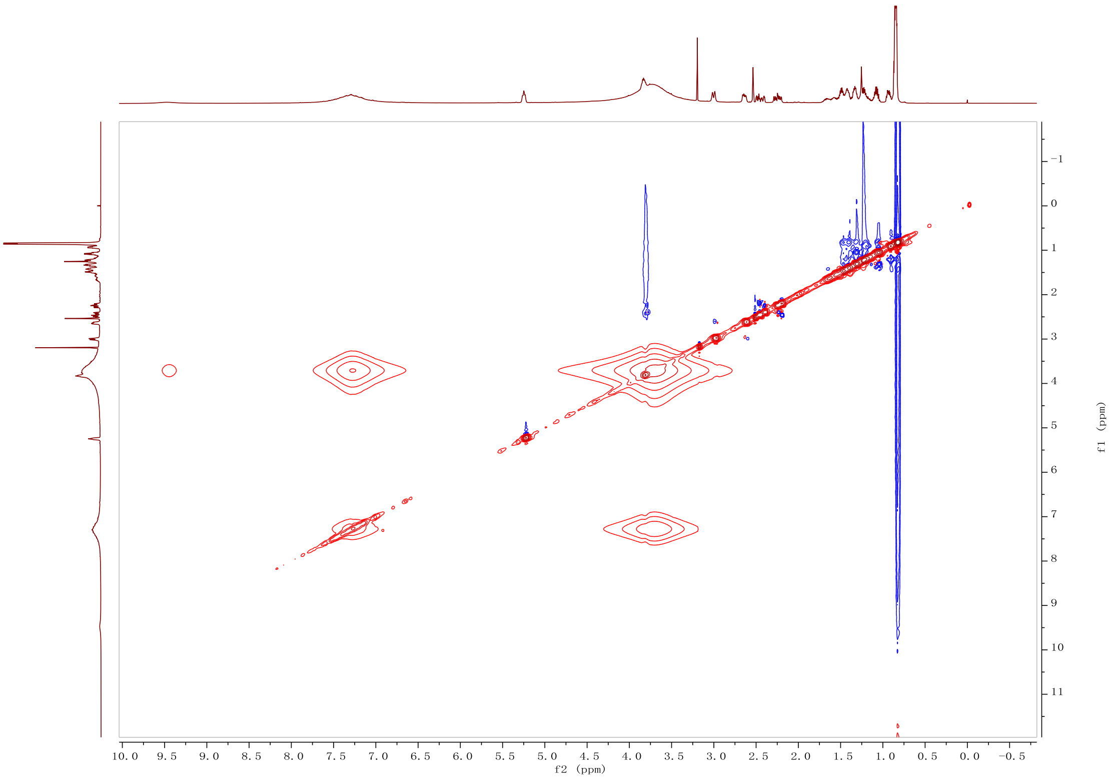

Figure S21. NOESY of **2** in DMSO-d_6 (600 MHz).

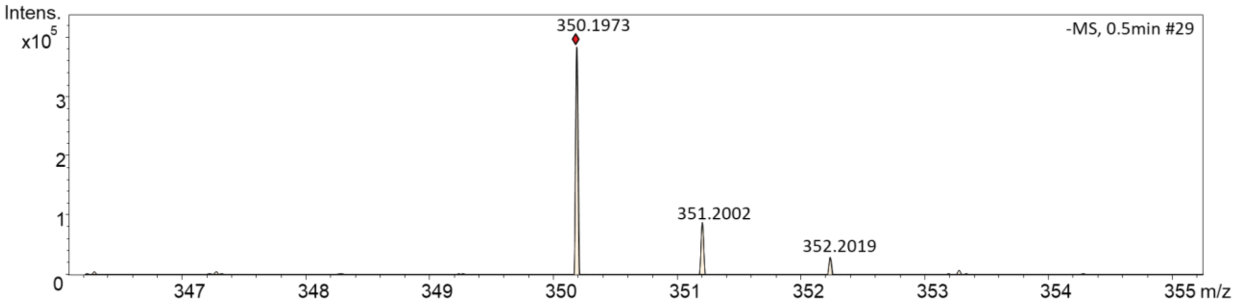

Figure S22. The HRESIMS spectrum of **3**

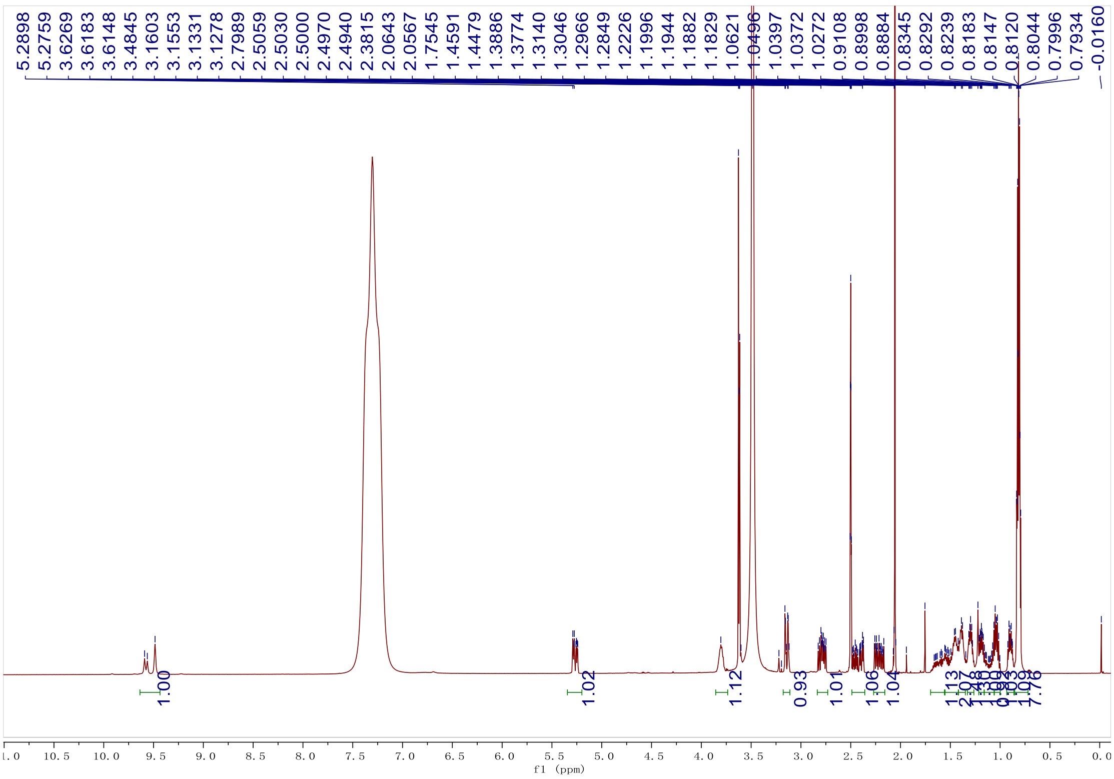

Figure S23. ^{1}H NMR of **3** in DMSO-d_6 (600 MHz).

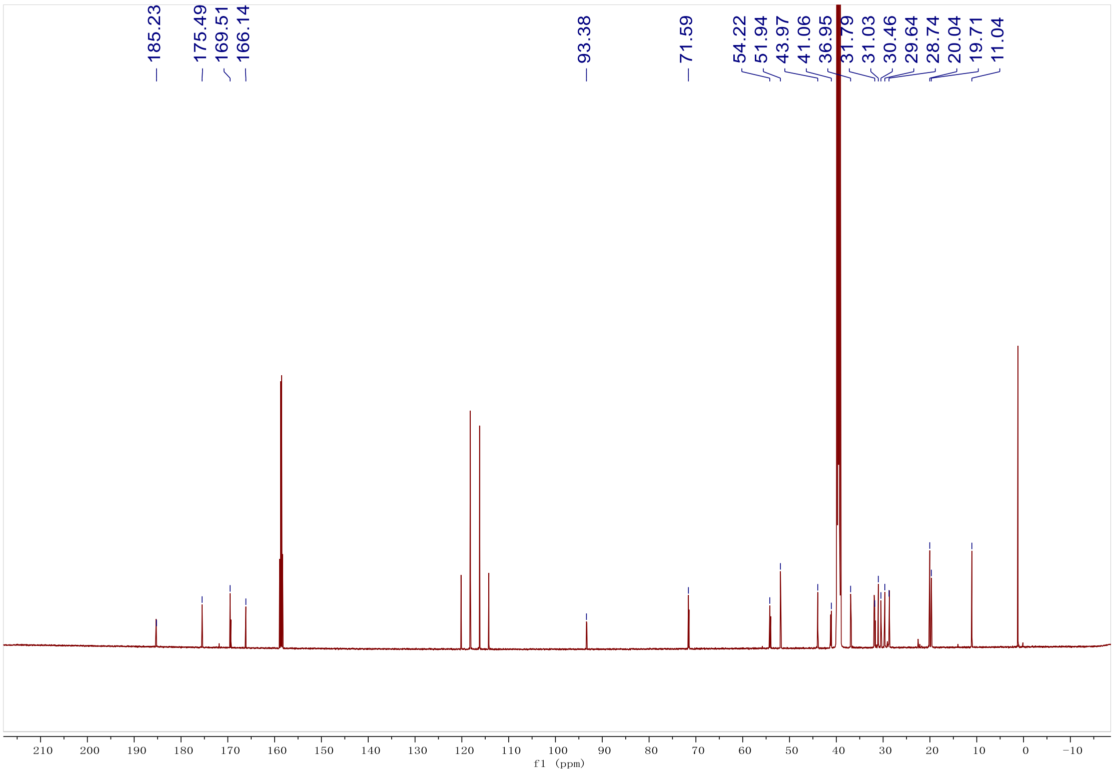

Figure S24. ^{13}C NMR of **3** in DMSO-d_6 (150 MHz).

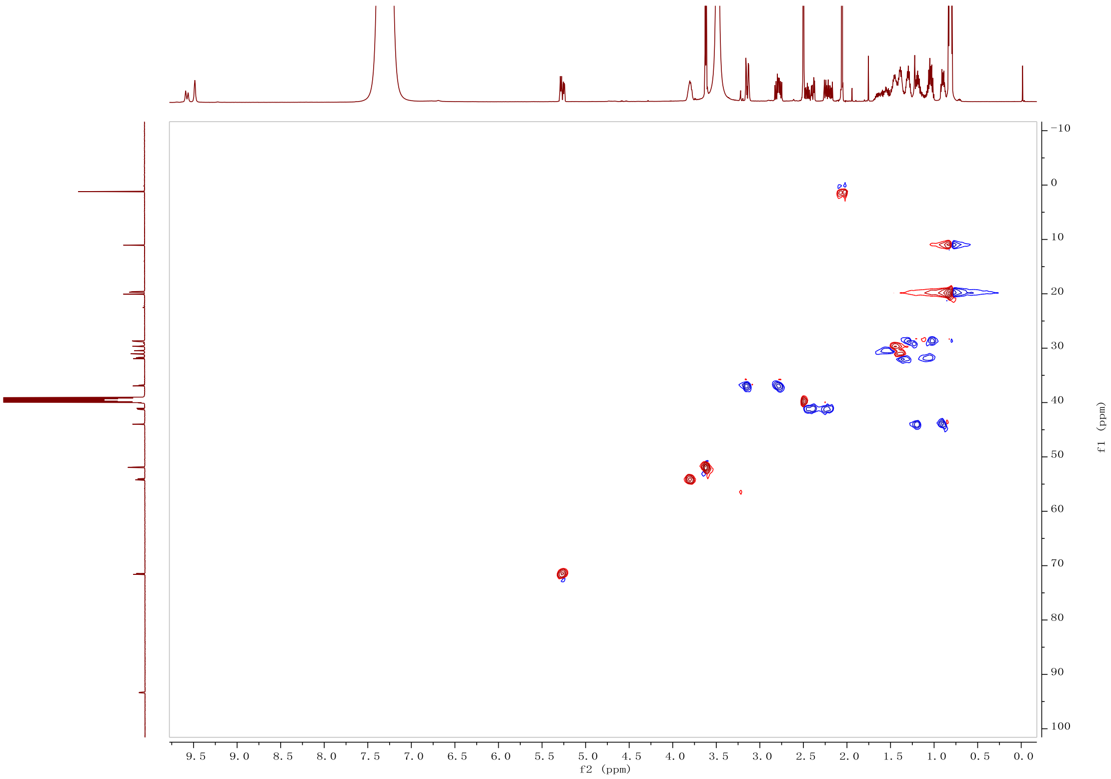

Figure S25. HSQC of **3** in DMSO-d_6 (600 and 150 MHz).

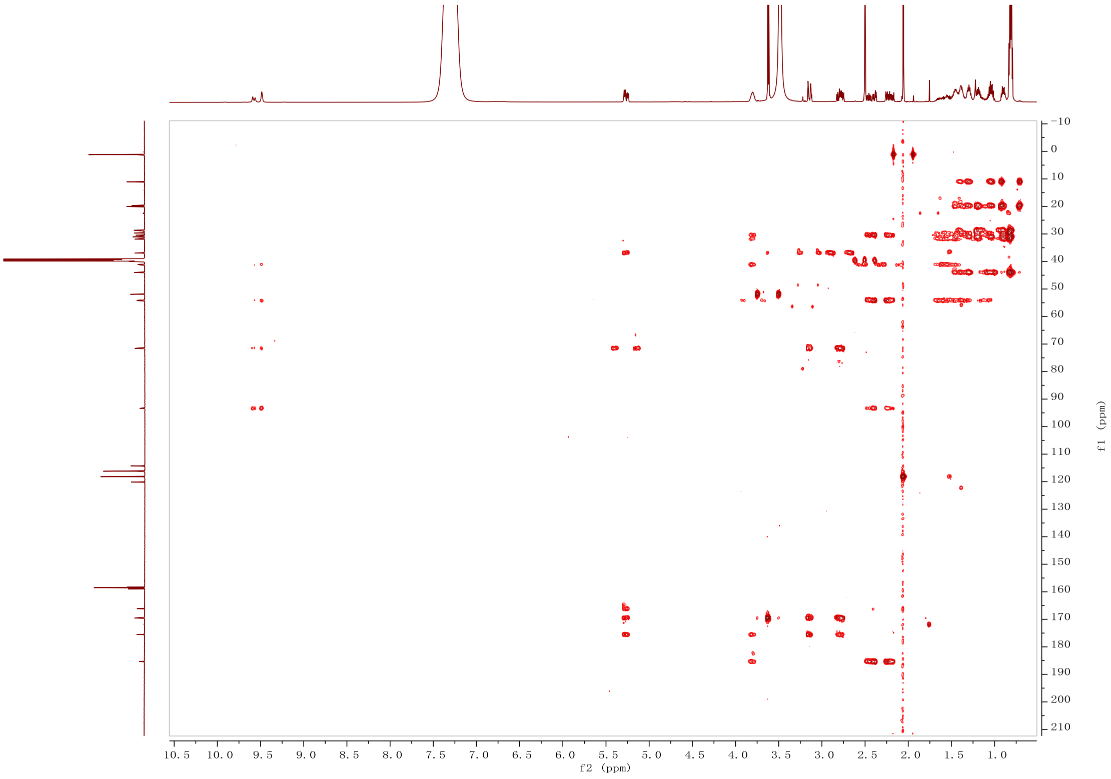

Figure S26. HMBC of **3** in DMSO-d_6 (600 and 150 MHz).

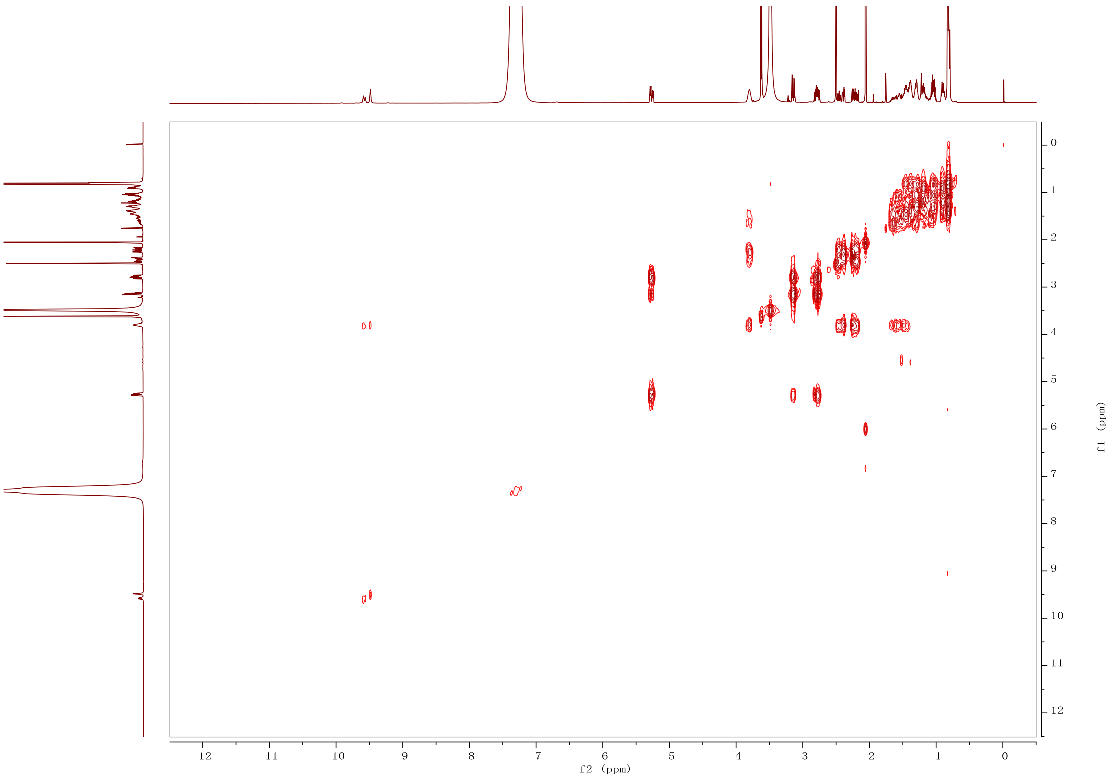

Figure S27. ^{1}H-^{1}H COSY of **3** in DMSO-d_6 (600 MHz).

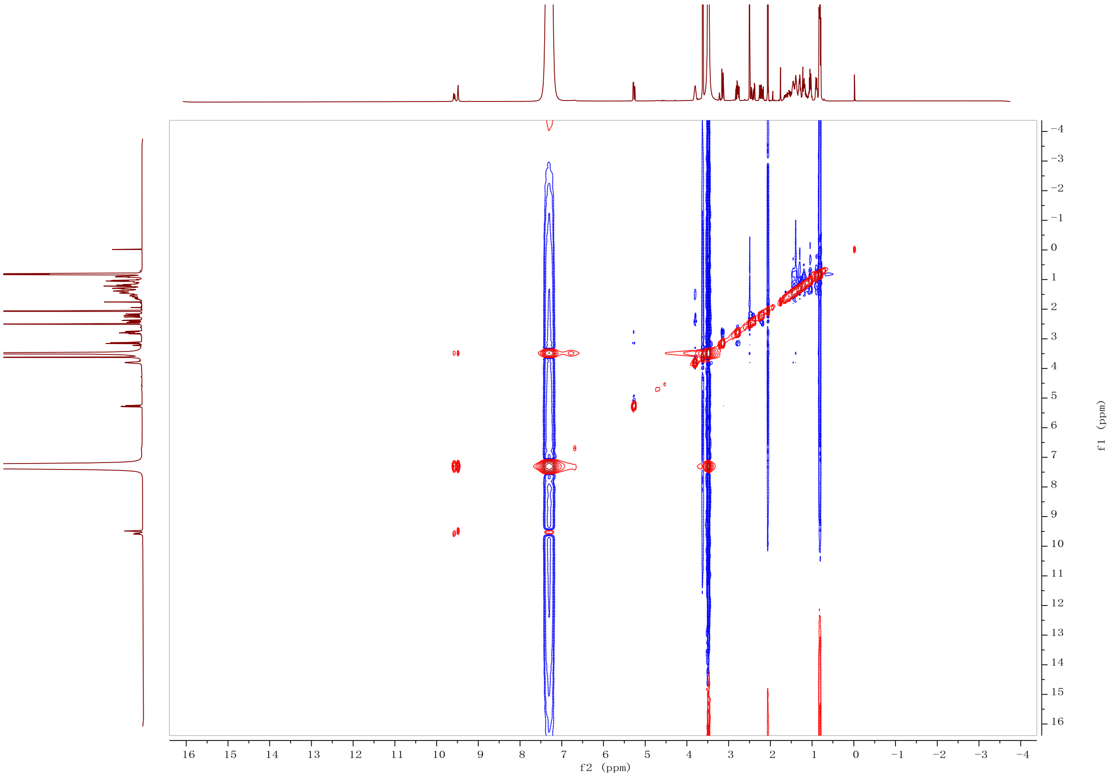

Figure S28. NOESY of **3** in DMSO-d_6 (600 MHz).

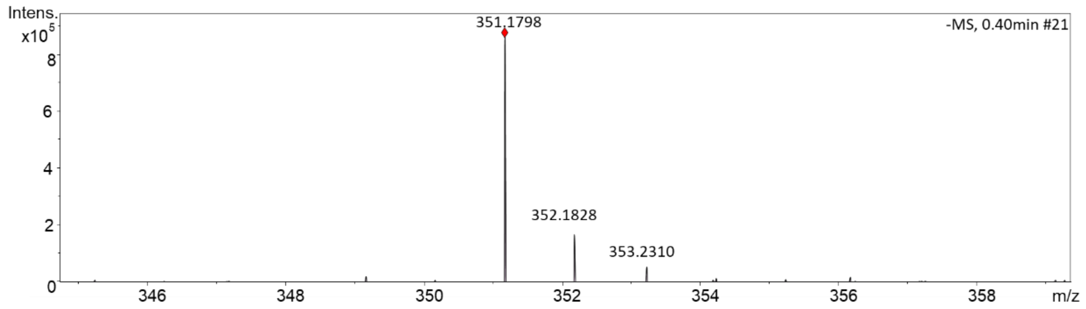

Figure S29. The HRESIMS spectrum of **4**

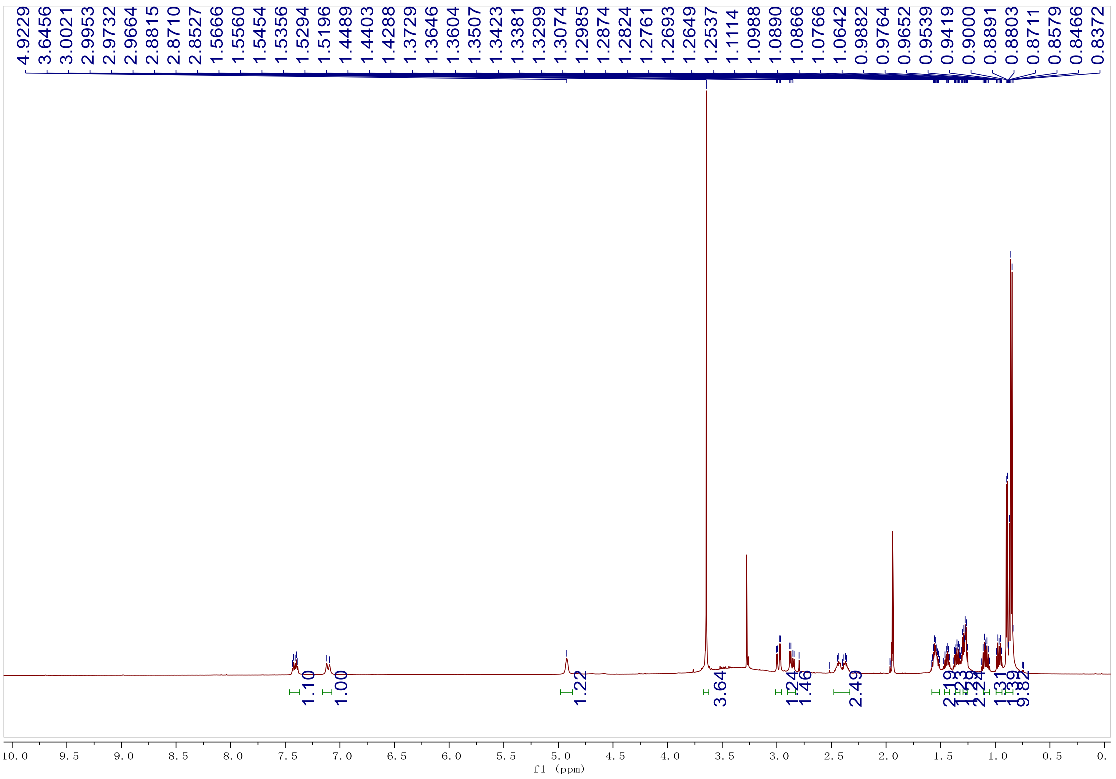

Figure S30. ^{1}H NMR of **4** in CD_3CN (600 MHz).

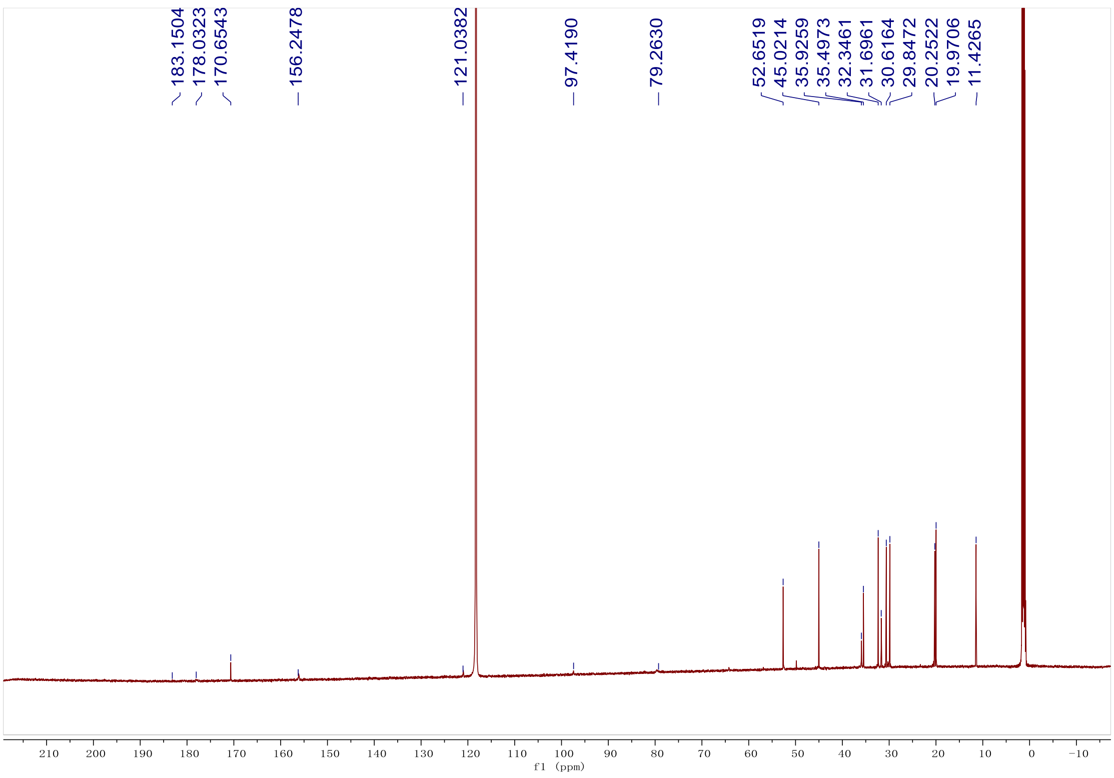

Figure S31. ^{13}C NMR of **4** in CD_3CN (150 MHz).

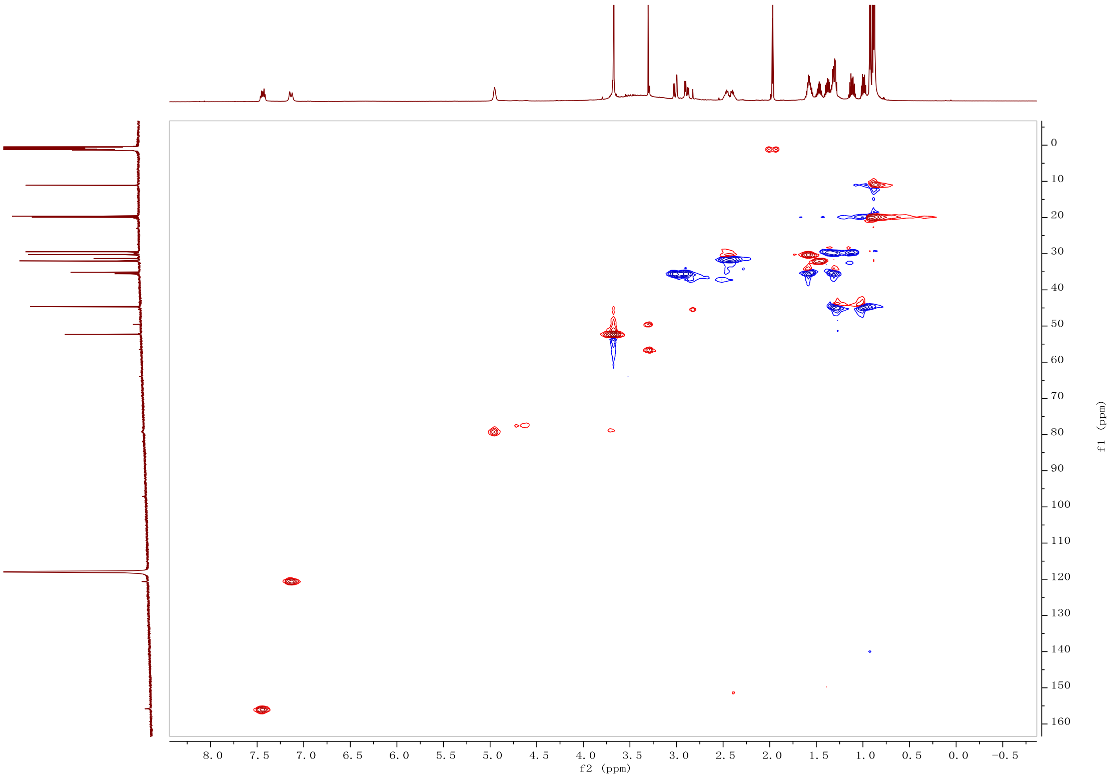

Figure S32. HSQC of **4** in CD_3CN (600 and 150 MHz).

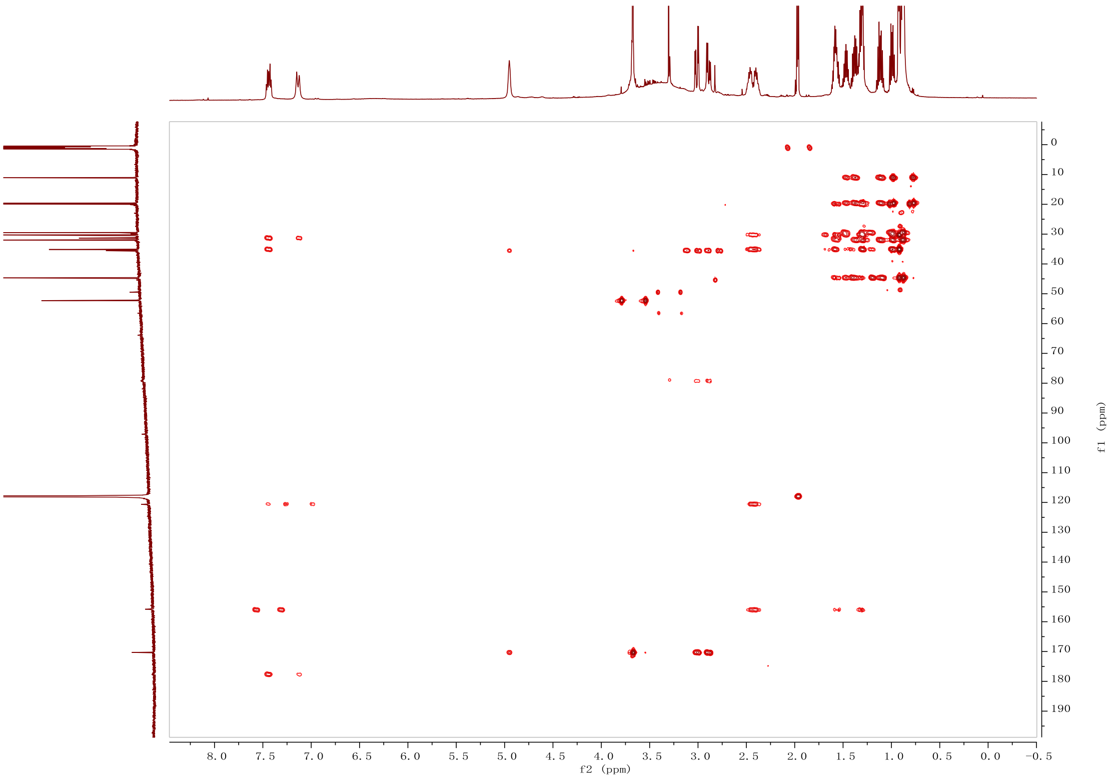

Figure S33. HMBC of **4** in CD_3CN (600 and 150 MHz).

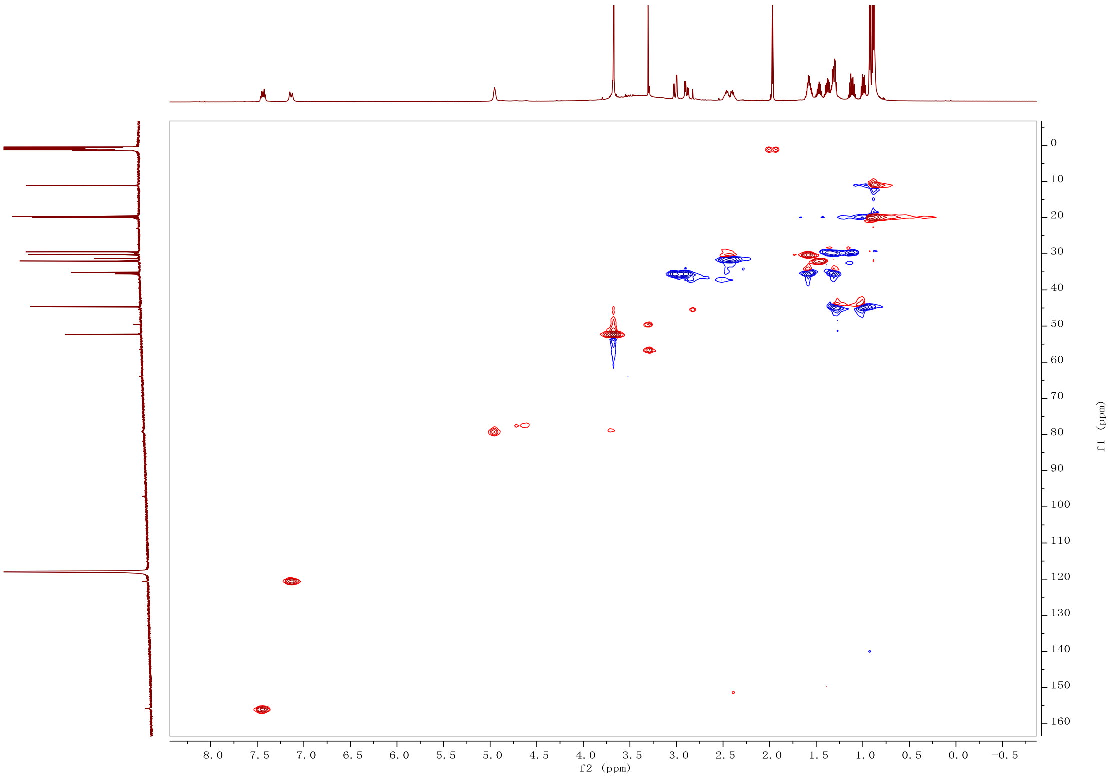

Figure S34. ^{1}H-^{1}H COSY of **4** in CD_3CN (600 MHz).

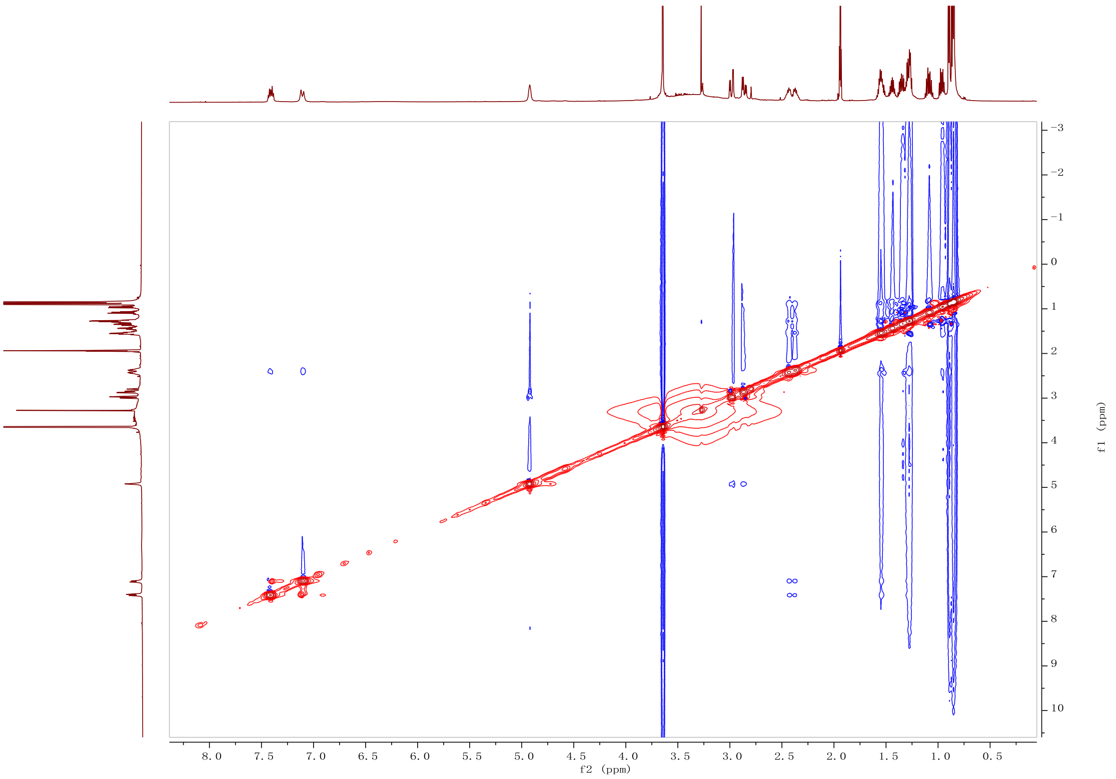

Figure S35. NOESY of **4** in CD_3CN (600 MHz).

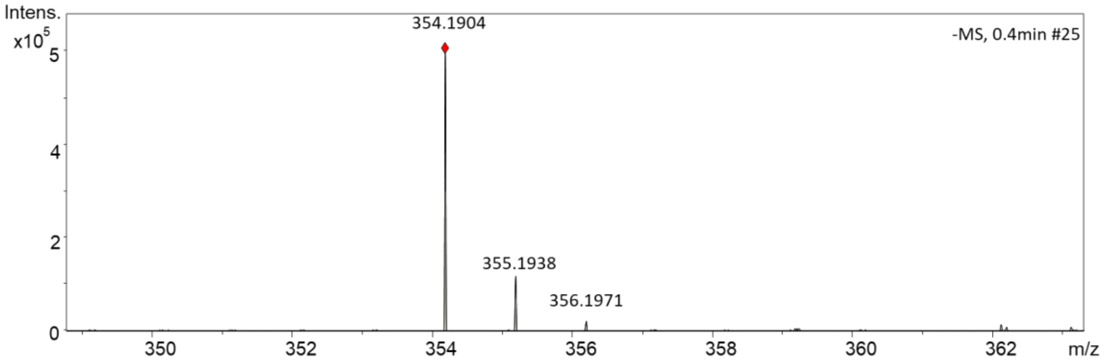

Figure S36. The HRESIMS spectrum of **5**.

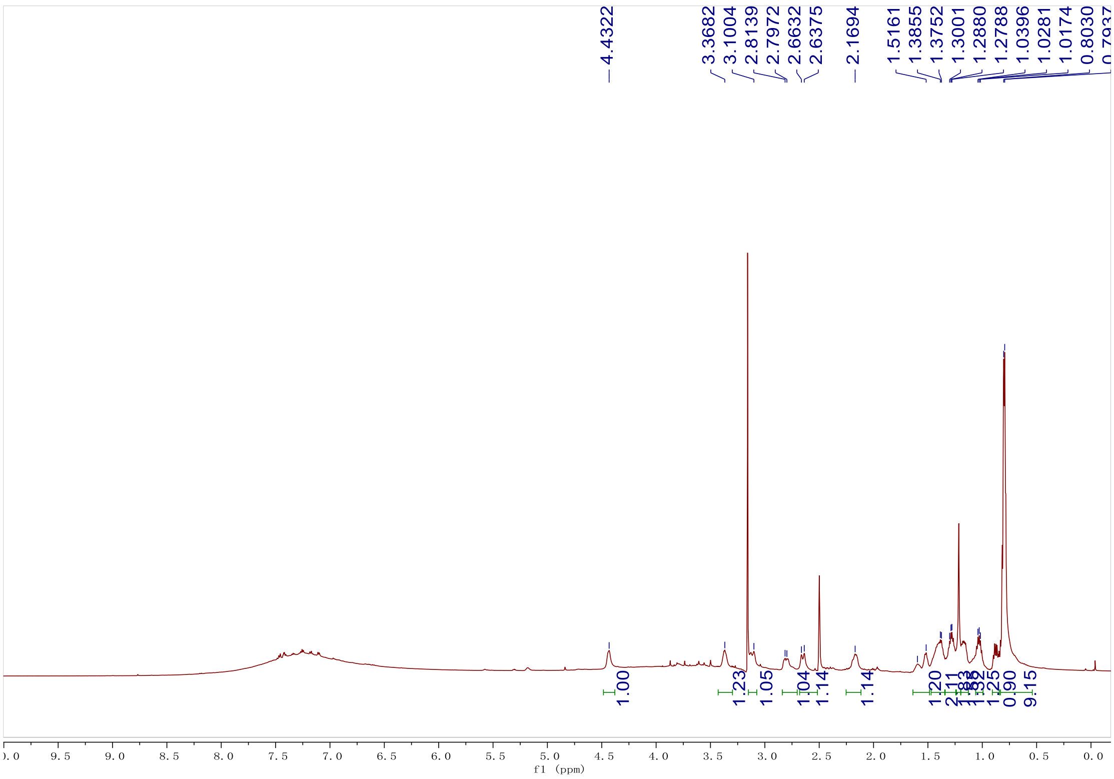

Figure S37. ^{1}H NMR of **5** in DMSO-d_6 (600 MHz).

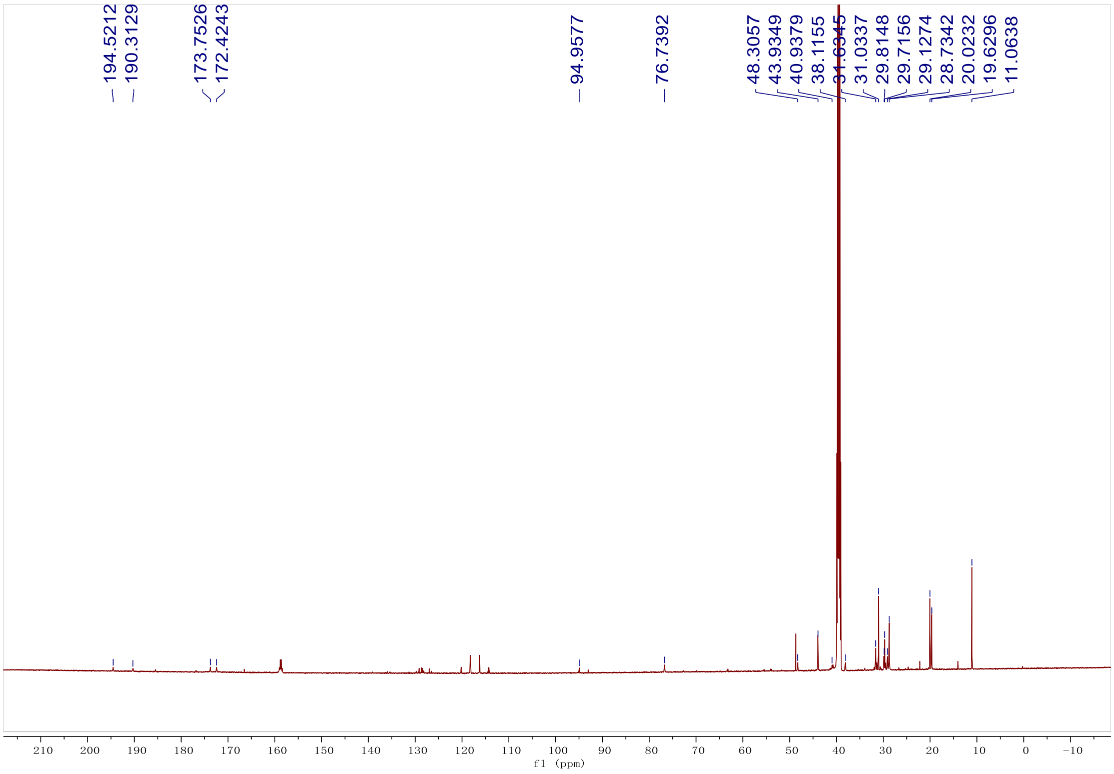

Figure S38. ^{13}C NMR of **5** in DMSO-d_6 (150 MHz).

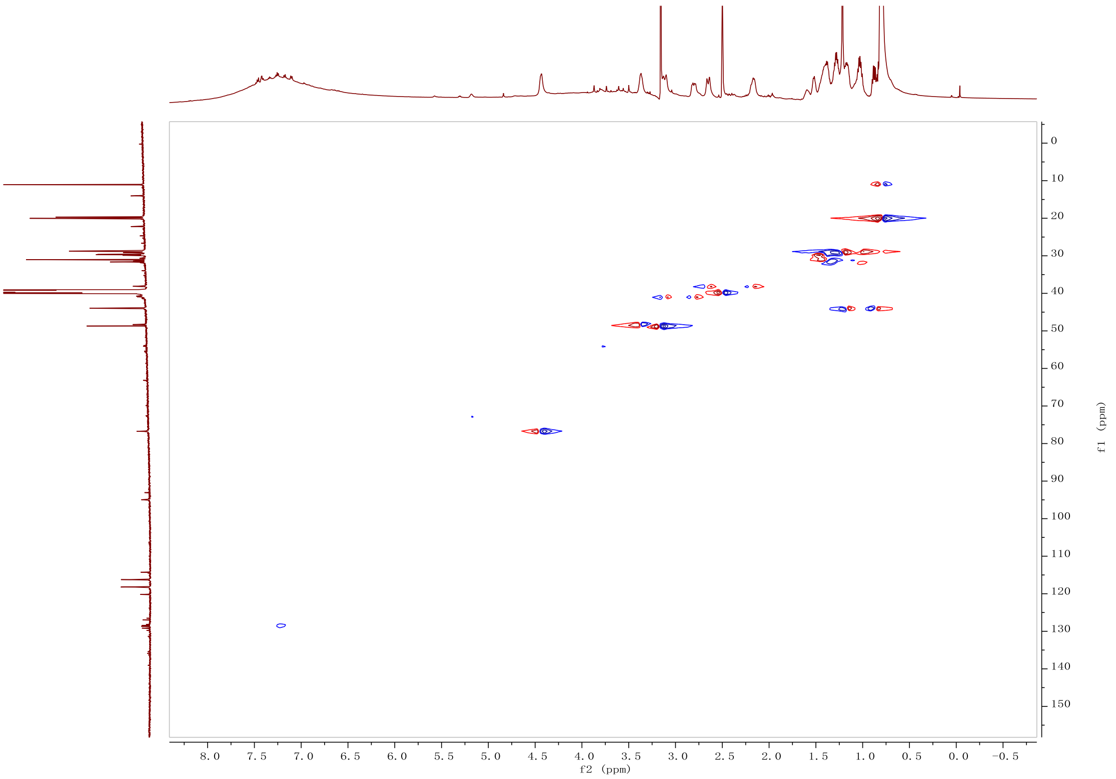

Figure S39. HSQC of **5** in DMSO-d_6 (600 and 150 MHz).

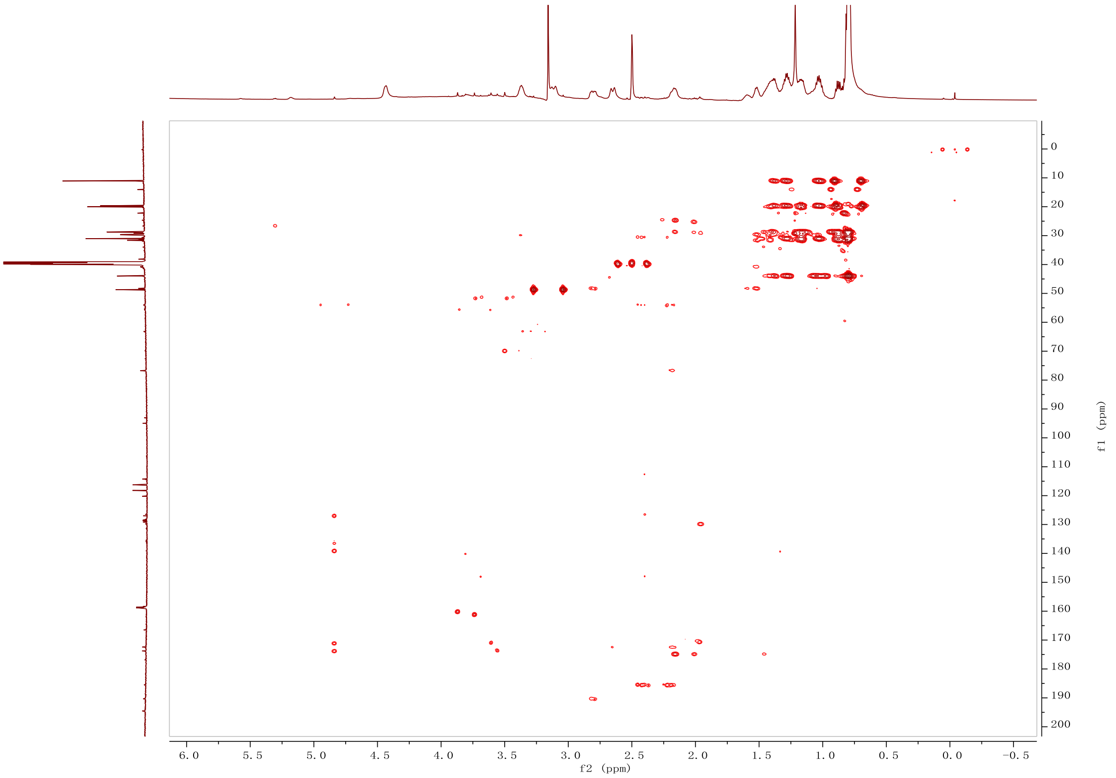

Figure S40. HMBC of **5** in DMSO-d_6 (600 and 150 MHz).

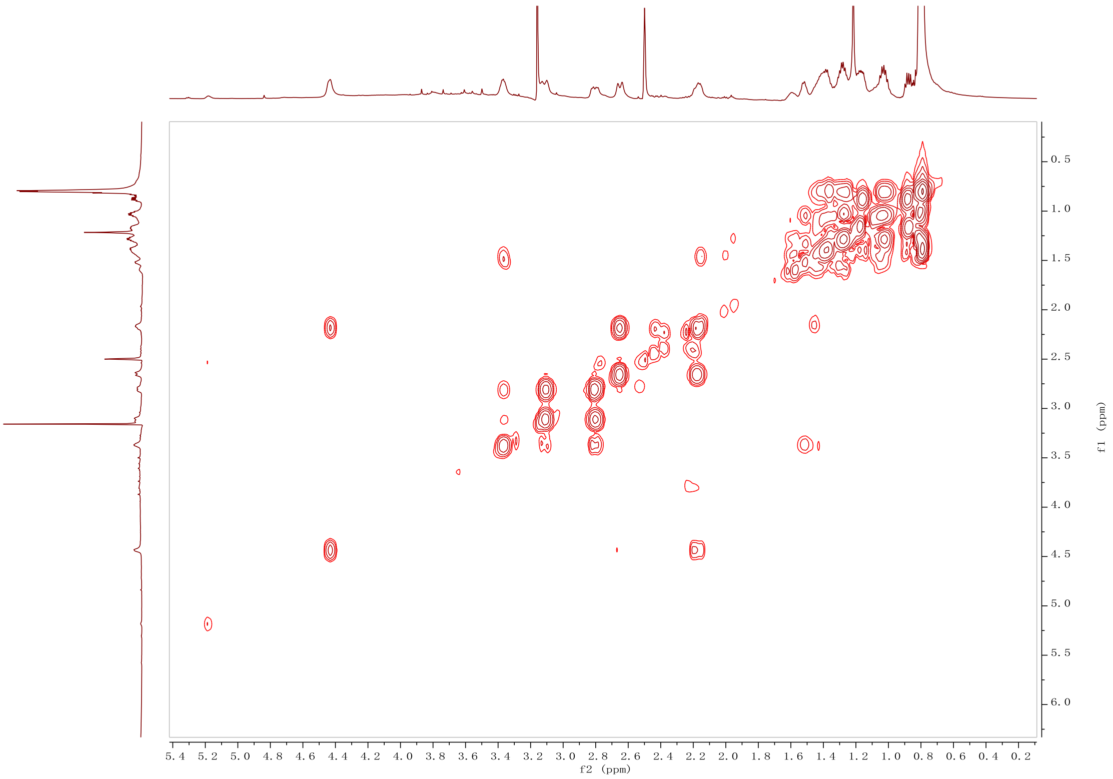

Figure S41. ^{1}H-^{1}H COSY of **5** in DMSO-d_6 (600 MHz).

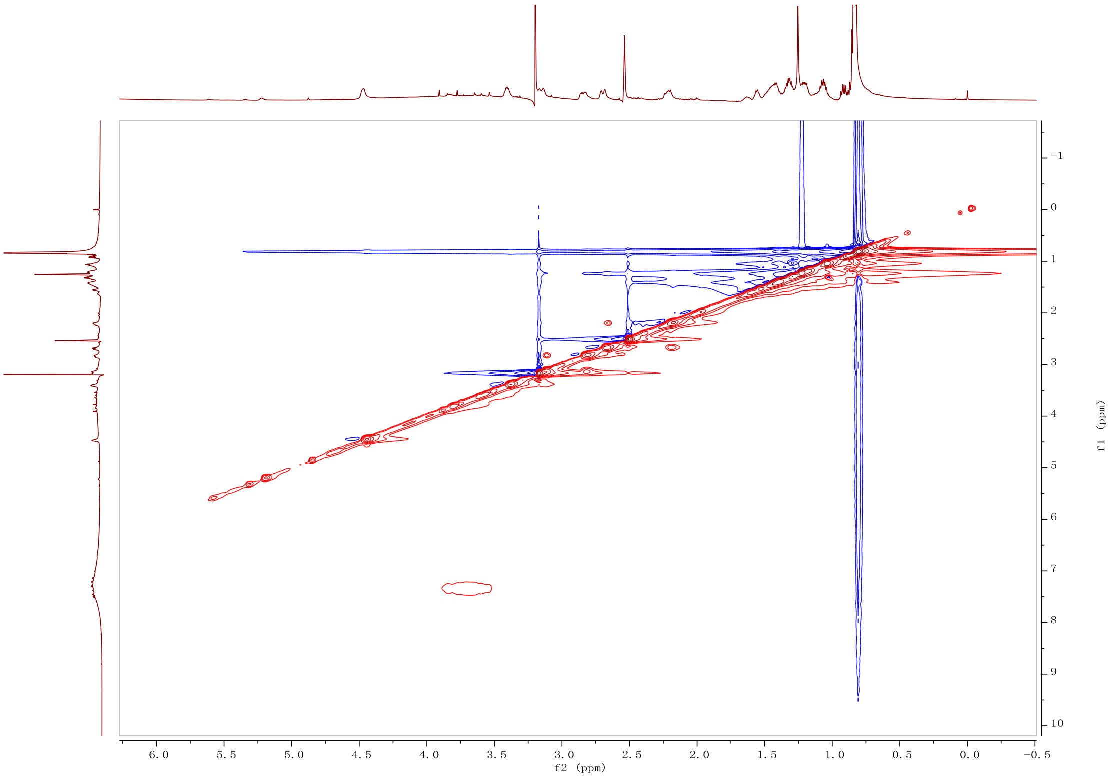

Figure S42. NOESY of **5** in DMSO-d_6 (600 MHz).

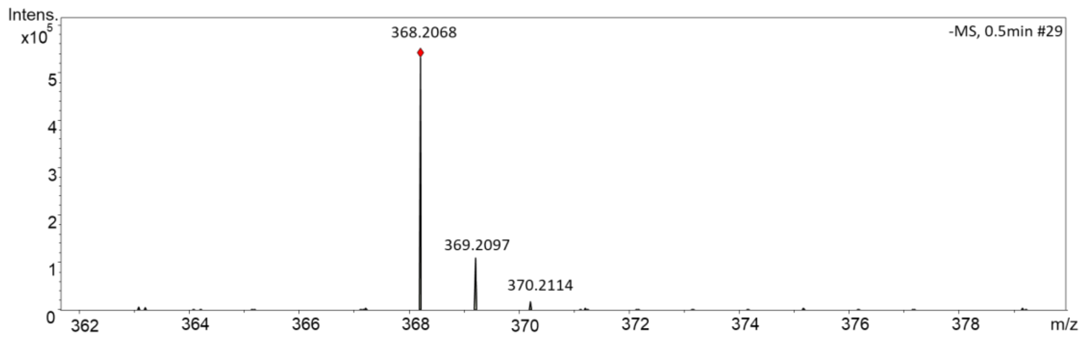

Figure S43. The HRESIMS spectrum of **6**

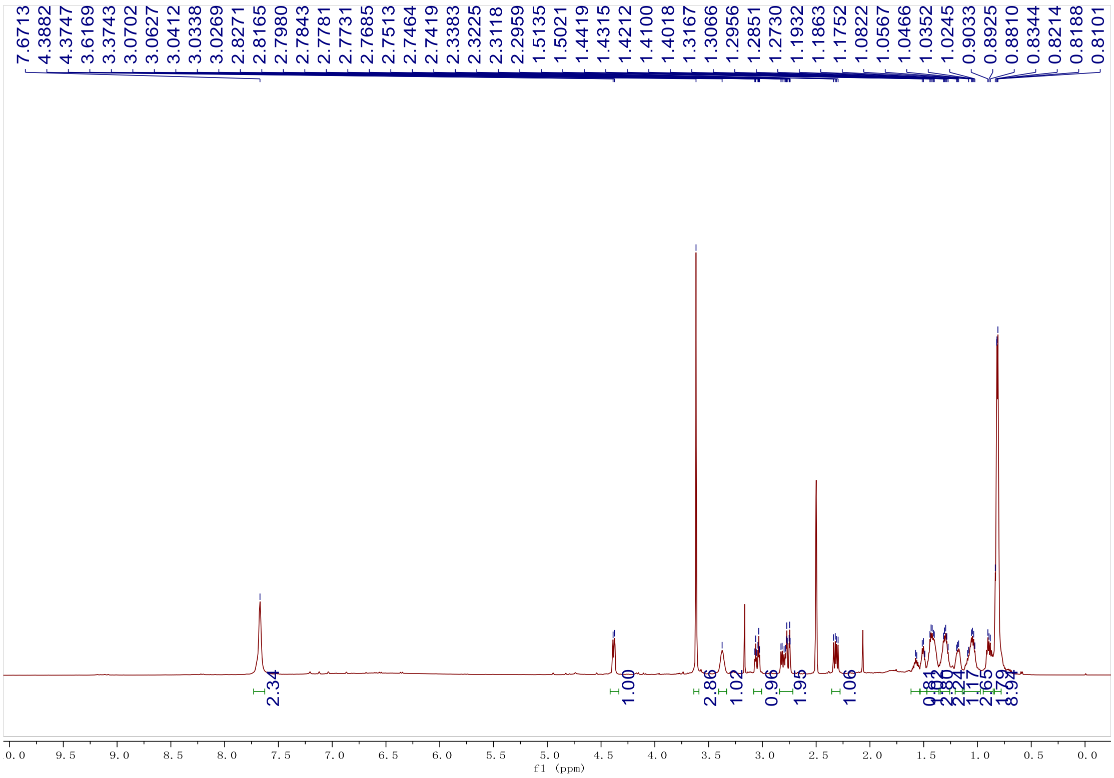

Figure S44. ^{1}H NMR of **6** in DMSO-d_6 (600 MHz).

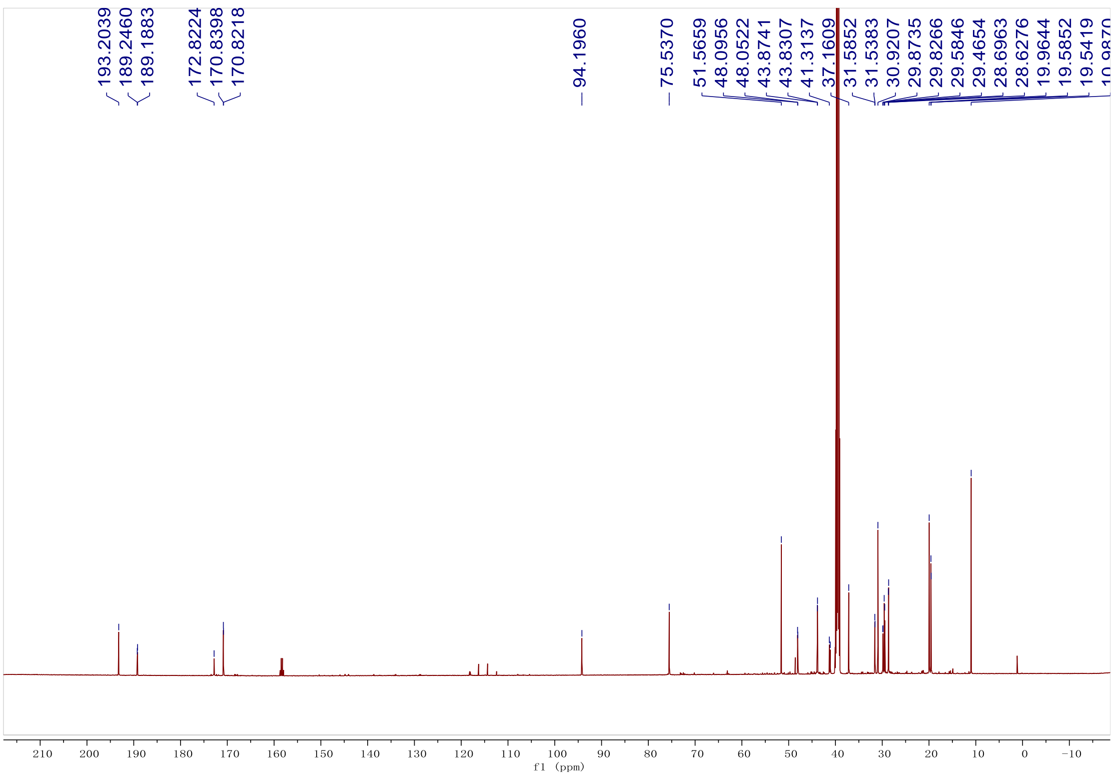

Figure S45. ^{13}C NMR of **6** in DMSO-d_6 (150 MHz).

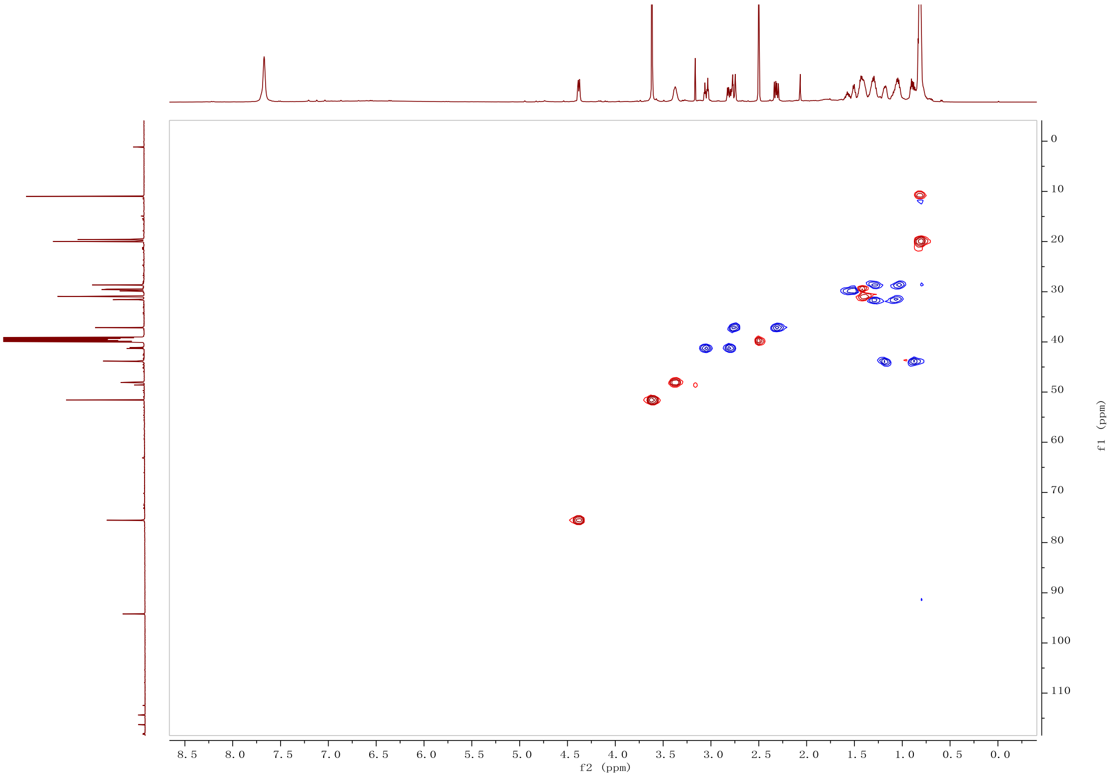

Figure S46. HSQC of **6** in DMSO-d_6 (600 and 150 MHz).

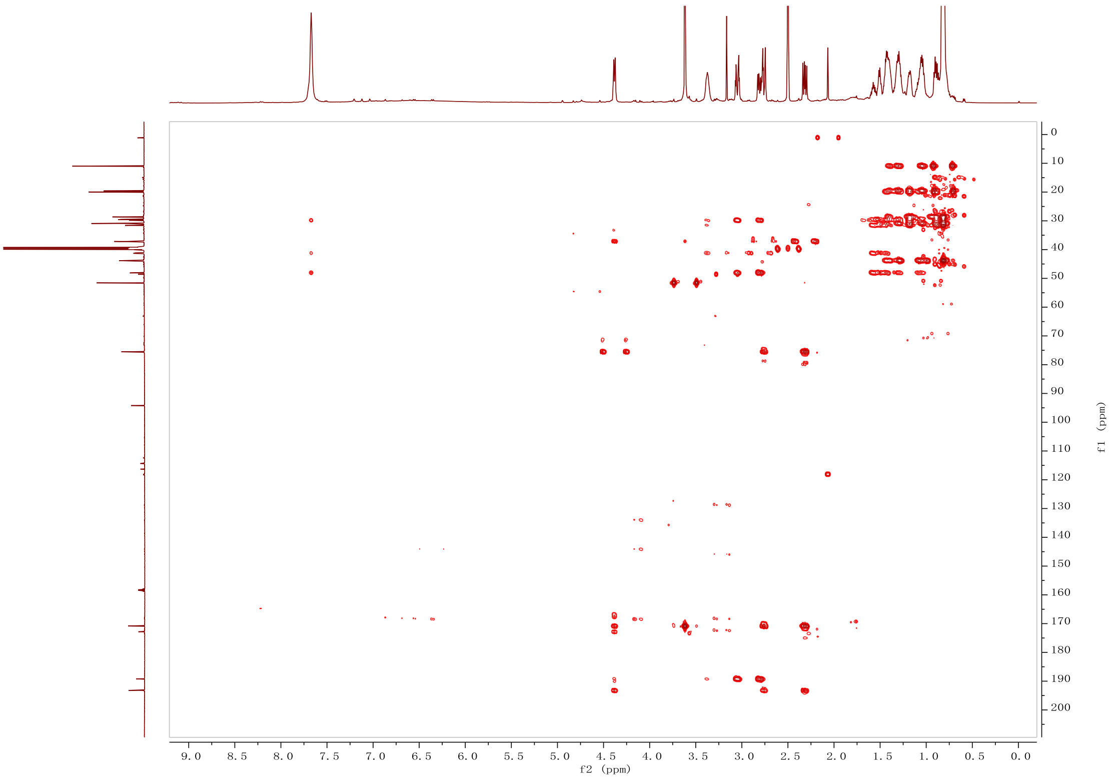

Figure S47. HMBC of **6** in DMSO-d_6 (600 and 150 MHz).

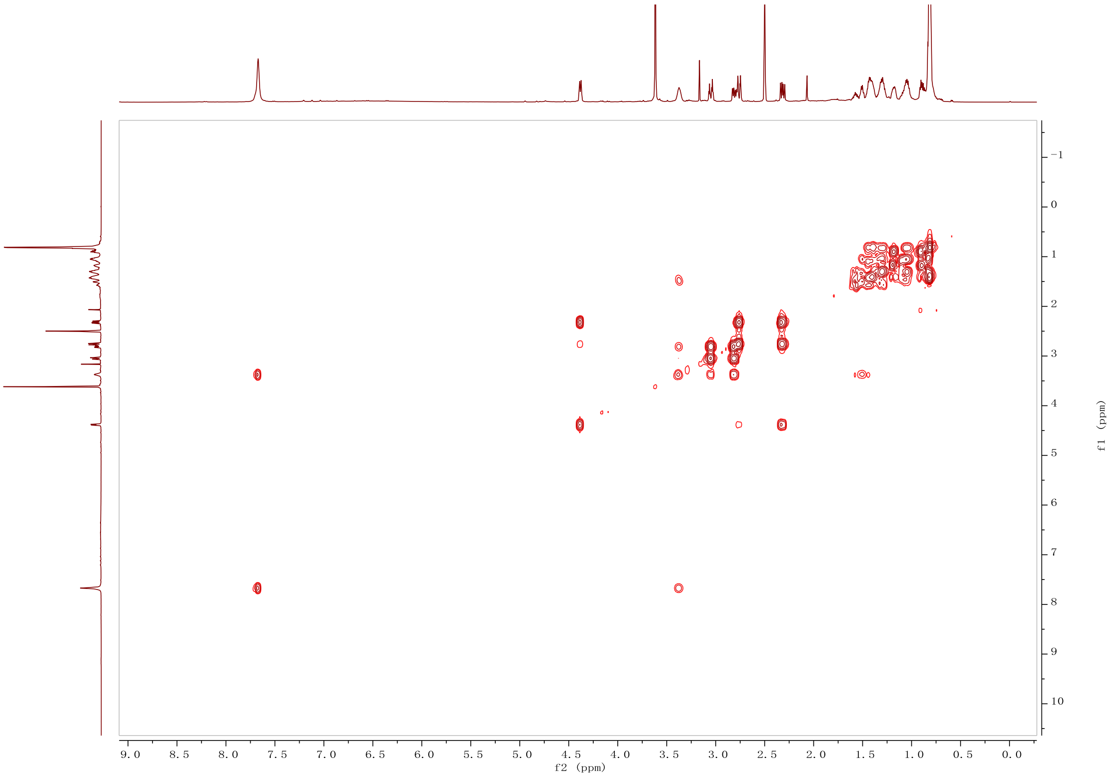

Figure S48. ^{1}H-^{1}H COSY of **6** in DMSO-d_6 (600 MHz).

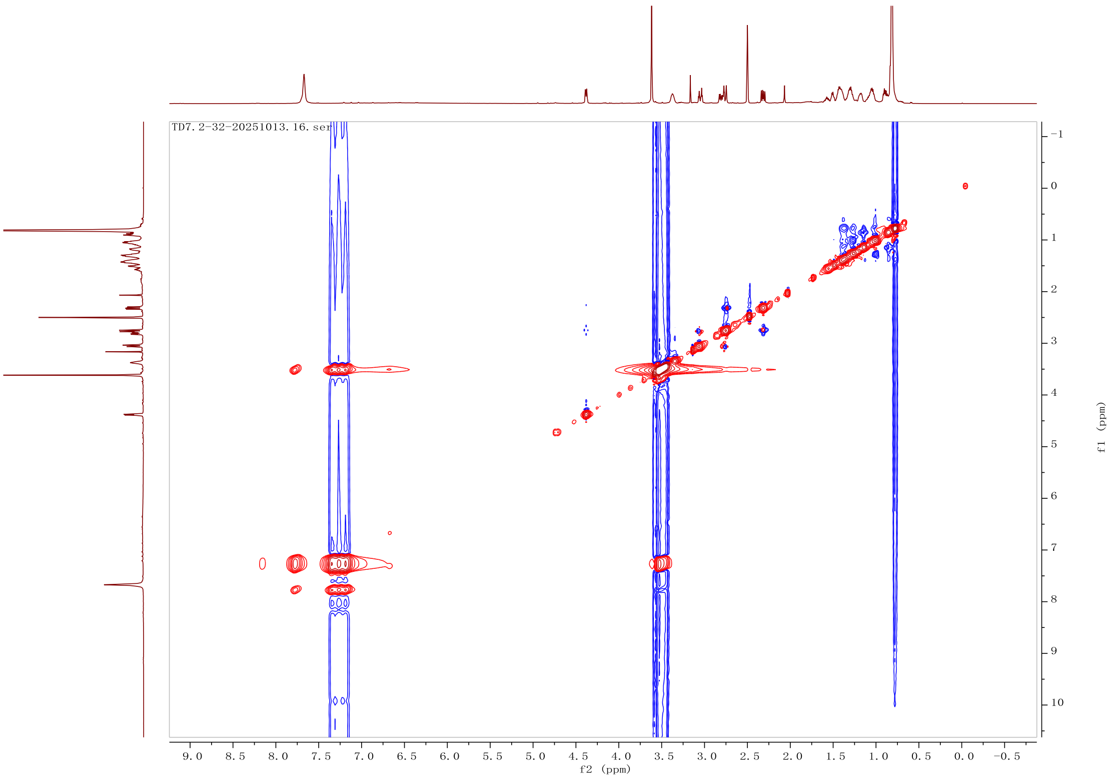

Figure S49. NOESY of **6** in DMSO-d_6 (600 MHz).

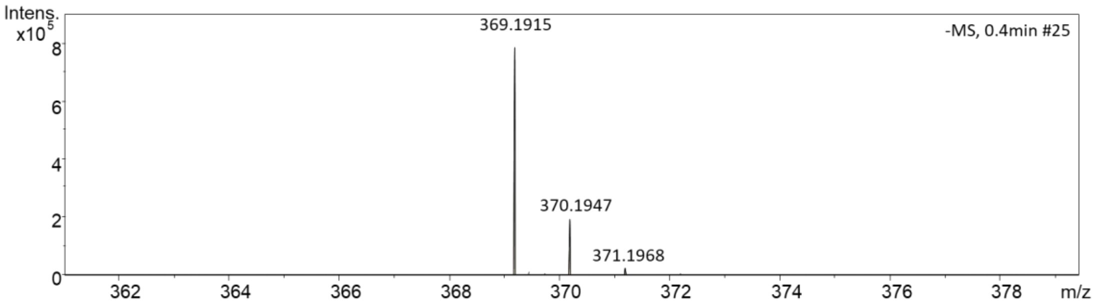

Figure S50. The HRESIMS spectrum of **7**

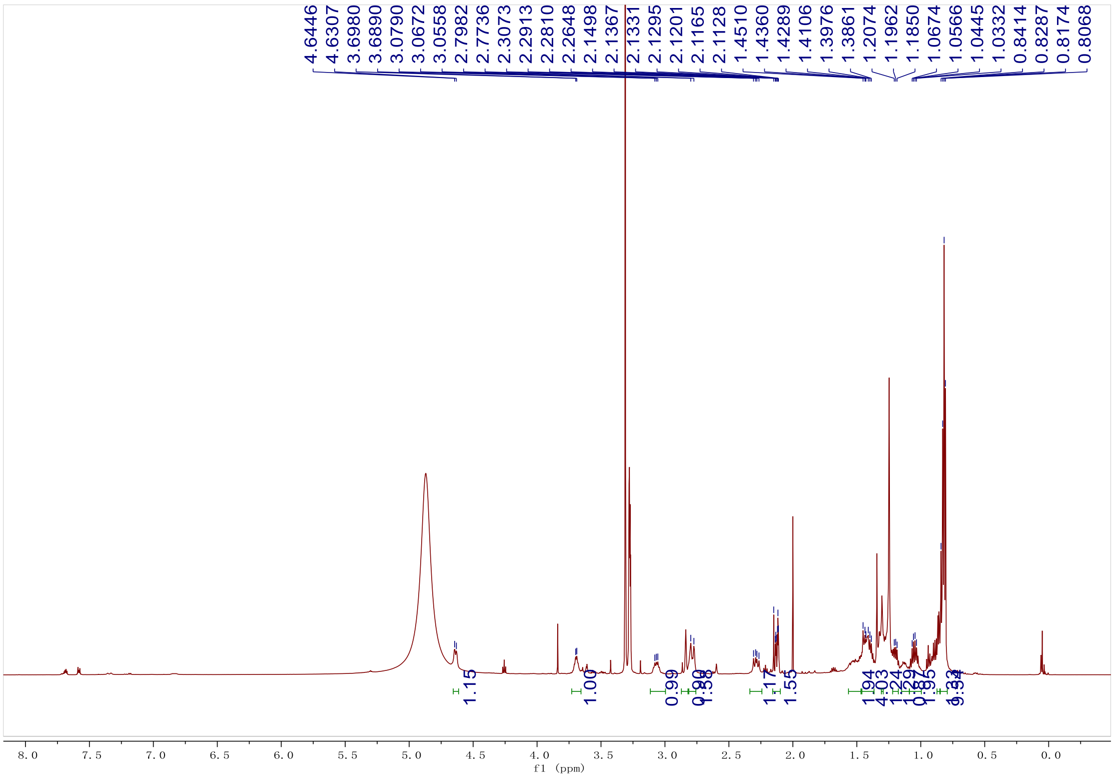

Figure S51. ^{1}H NMR of **7** in DMSO-d_6 (600 MHz).

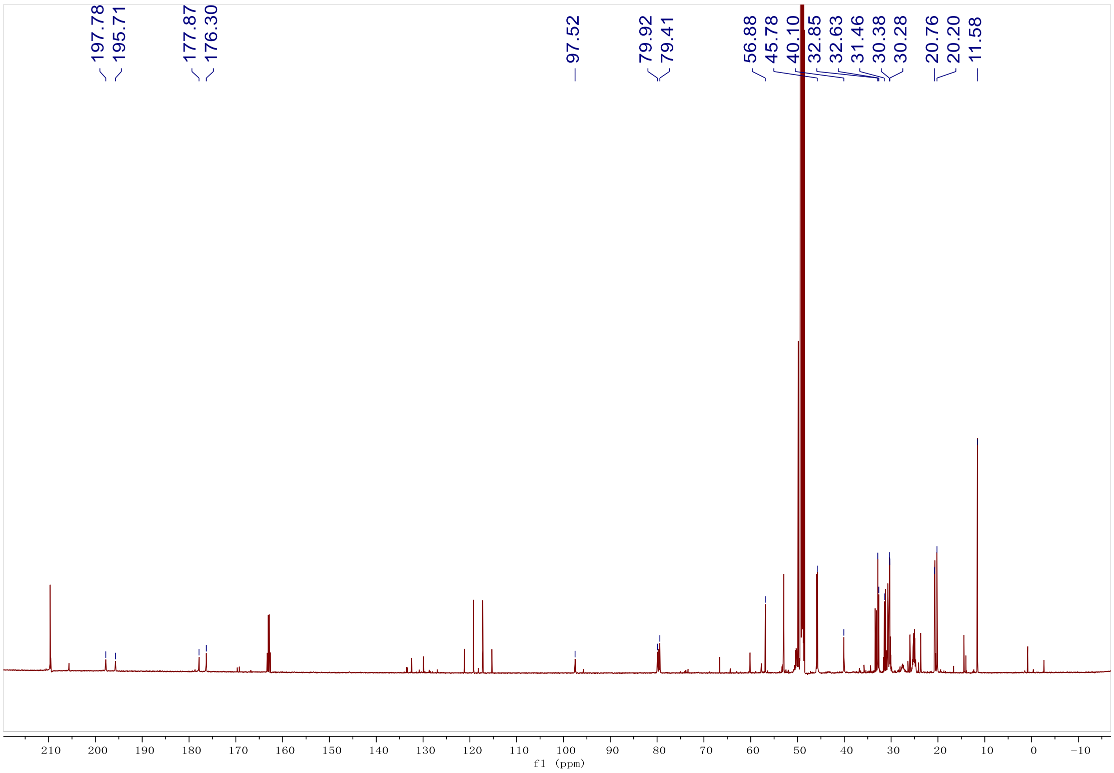

Figure S52. ^{13}C NMR of **7** in DMSO-d_6 (150 MHz).

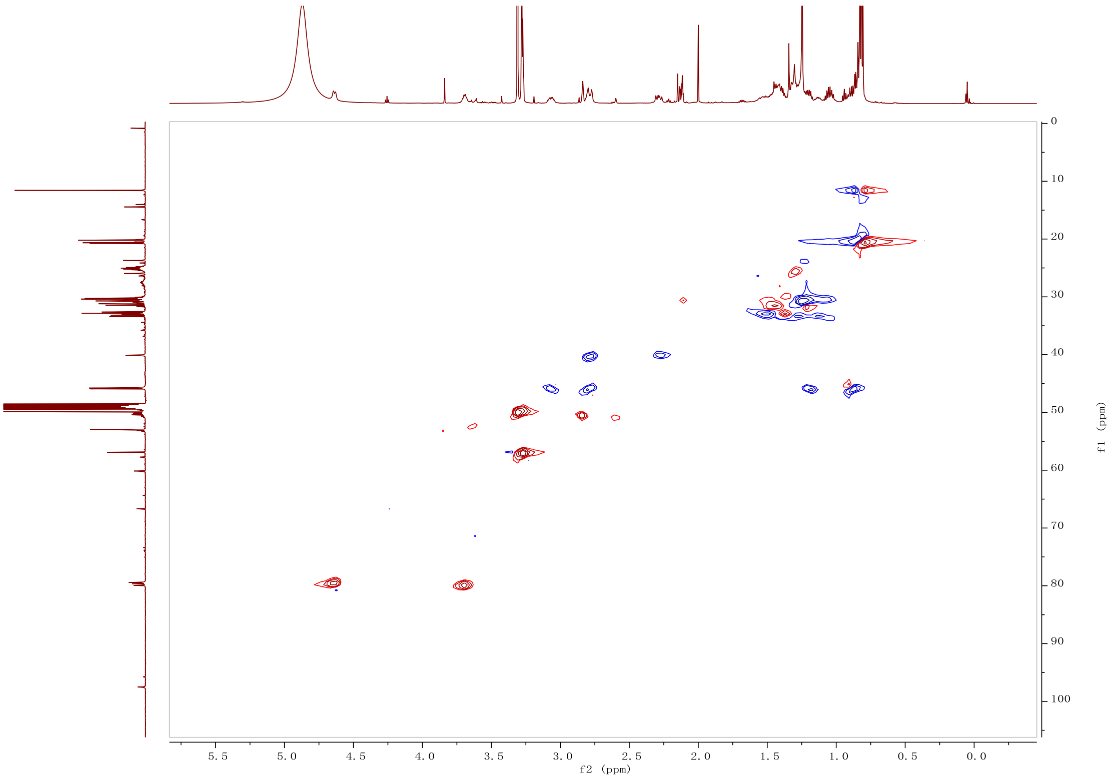

Figure S53. HSQC of **7** in DMSO-d_6 (600 and 150 MHz).

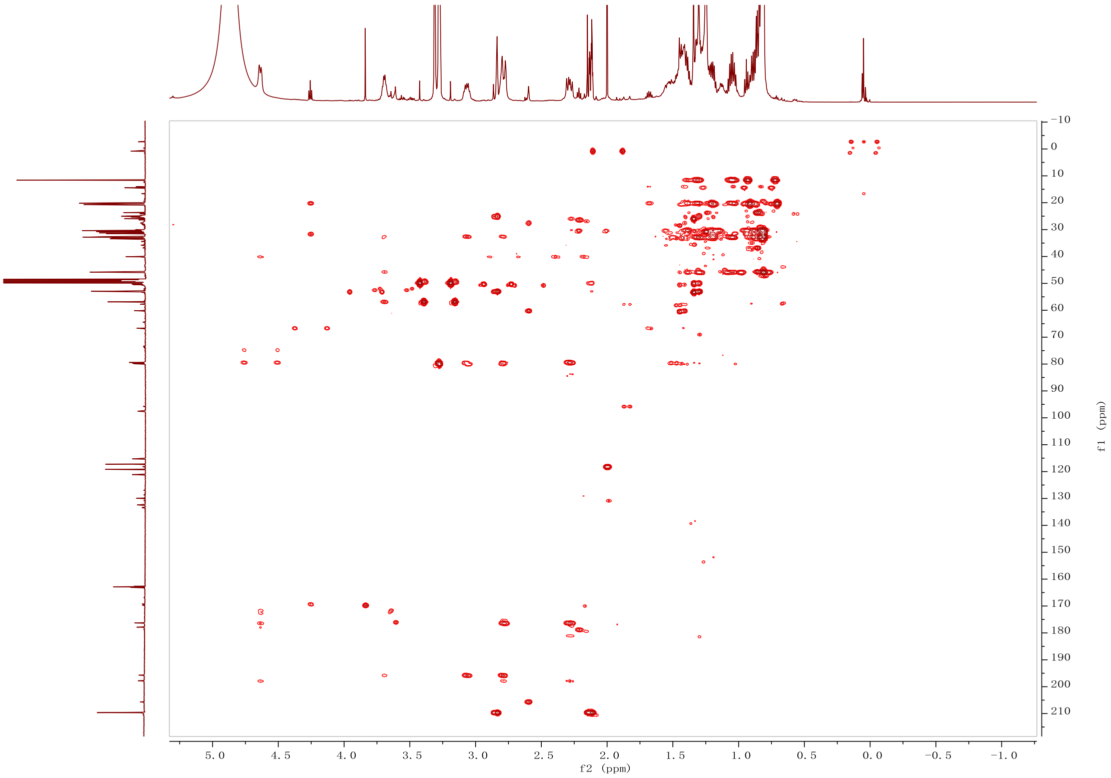

Figure S54. HMBC of **7** in DMSO-d_6 (600 and 150 MHz).

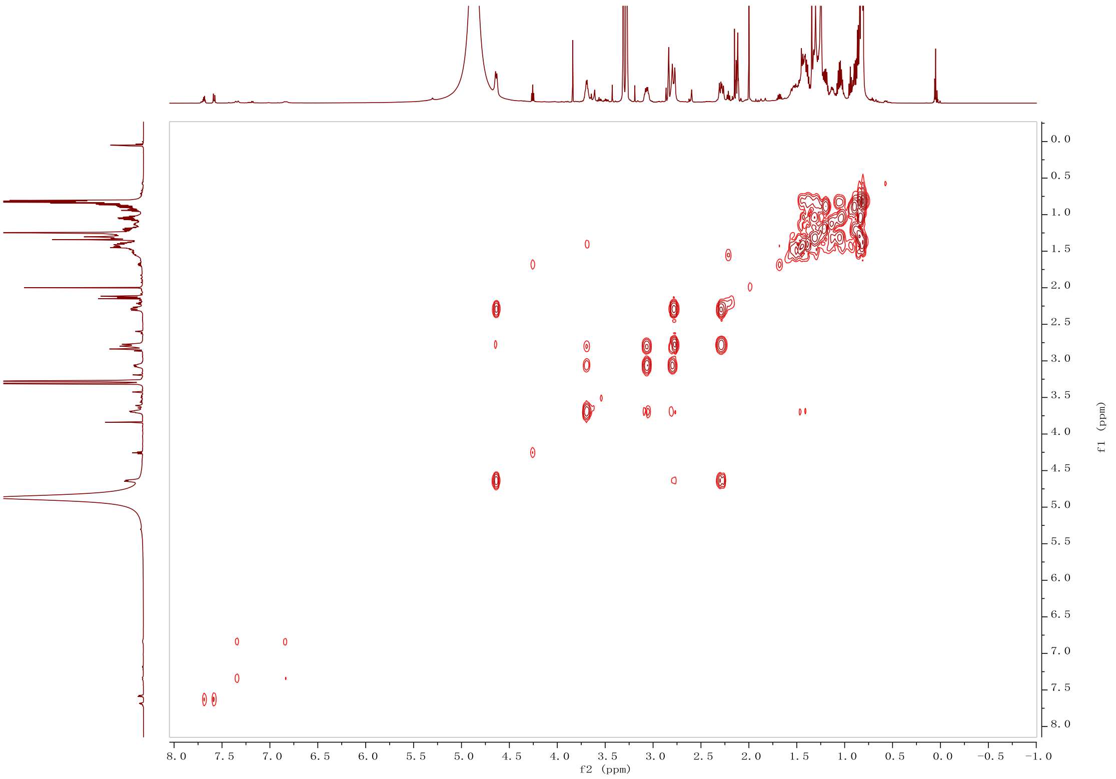

Figure S55. ^{1}H-^{1}H COSY of **7** in DMSO-d_6 (600 MHz).

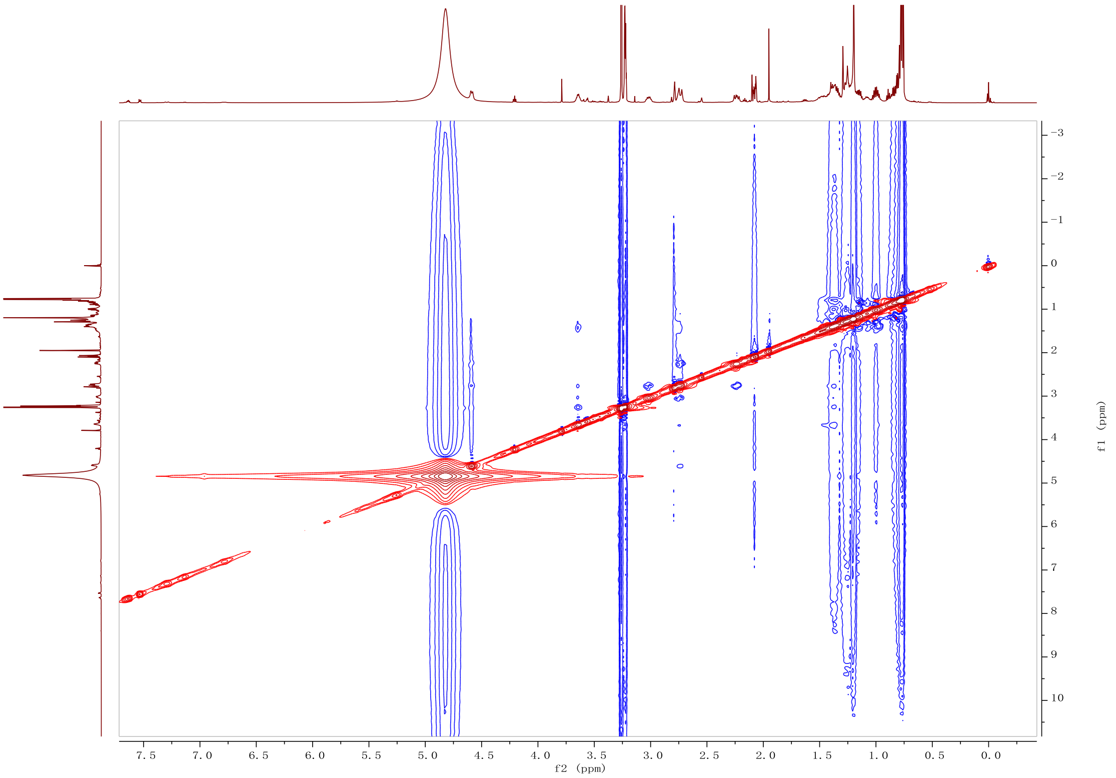

Figure S56. NOESY of **7** in DMSO-d_6 (600 MHz).

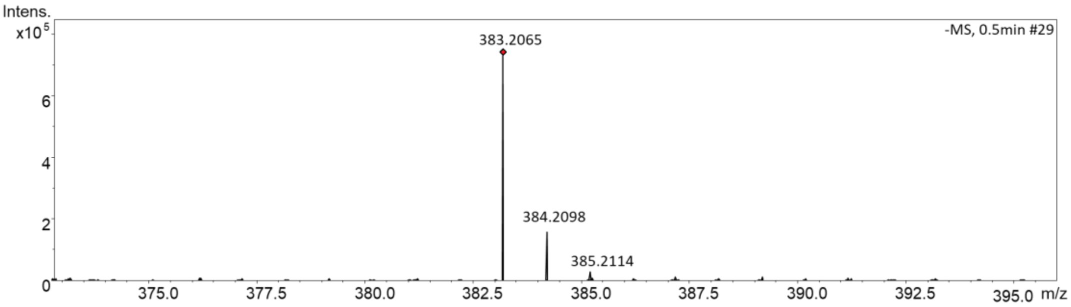

Figure S57. The HRESIMS spectrum of **8**

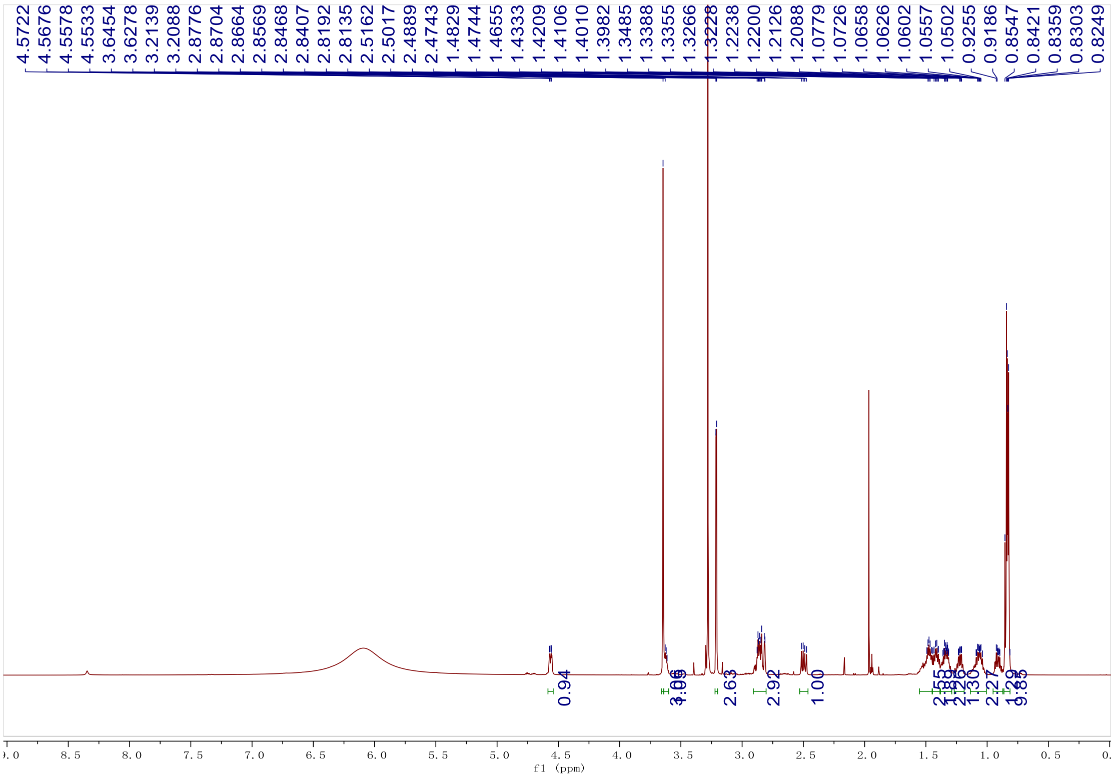

Figure S58. ^{1}H NMR of **8** in CD_3CN (600 MHz).

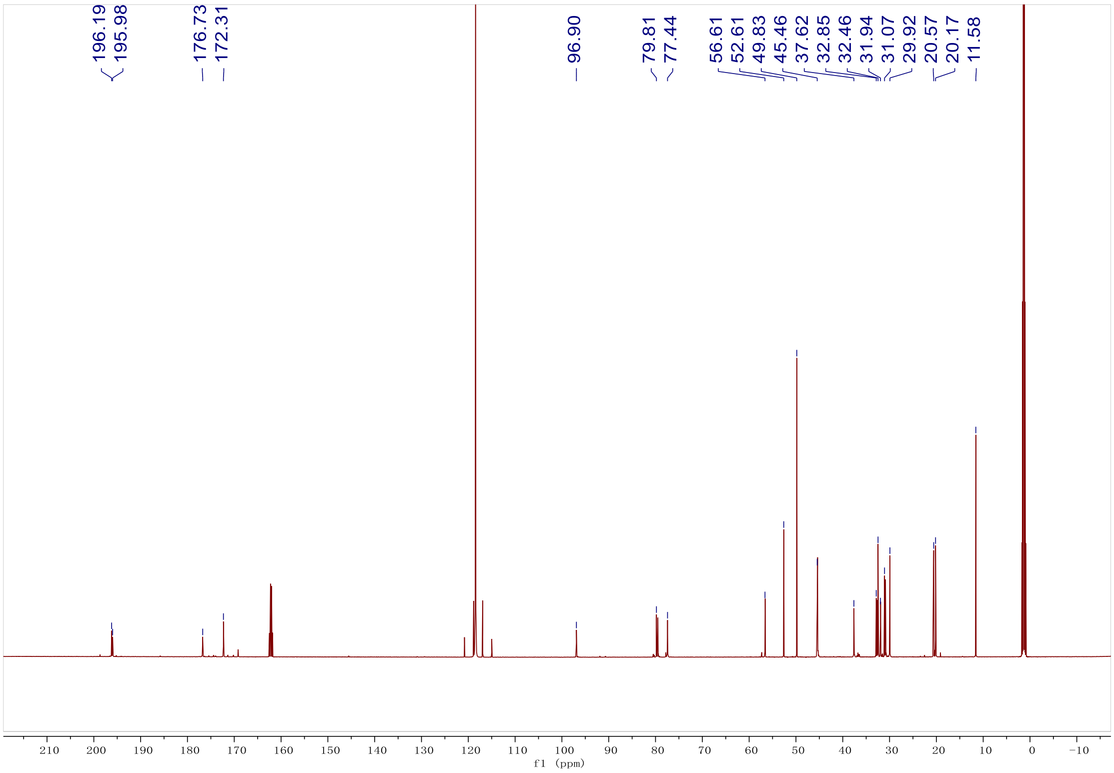

Figure S59. ^{13}C NMR of **8** in CD_3CN (150 MHz).

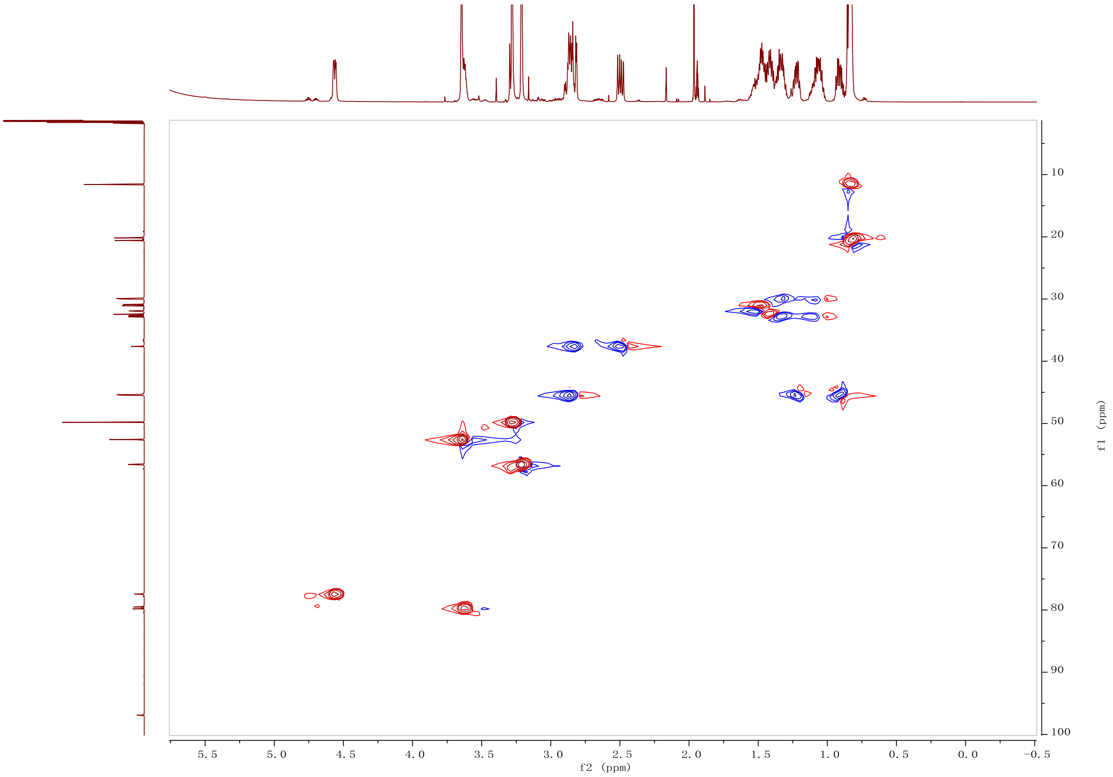

Figure S60. HSQC of **8** in CD_3CN (600 MHz).

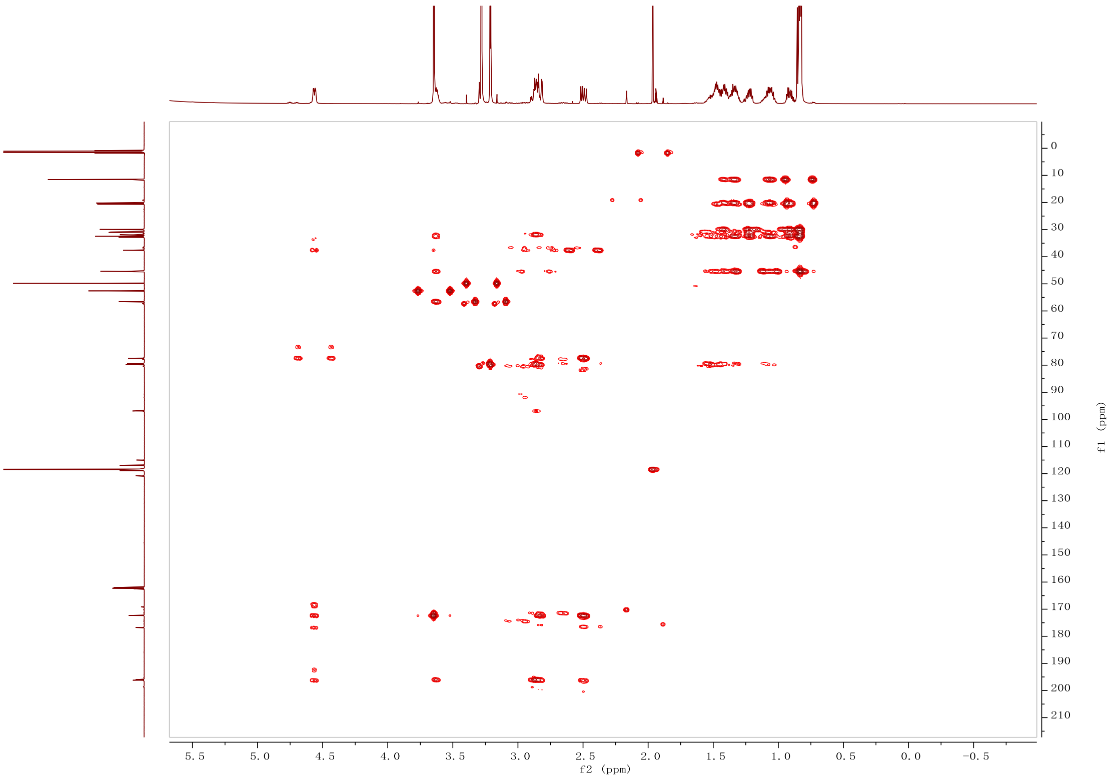

Figure S61. HMBC of **8** in CD_3CN (600 and 150 MHz).

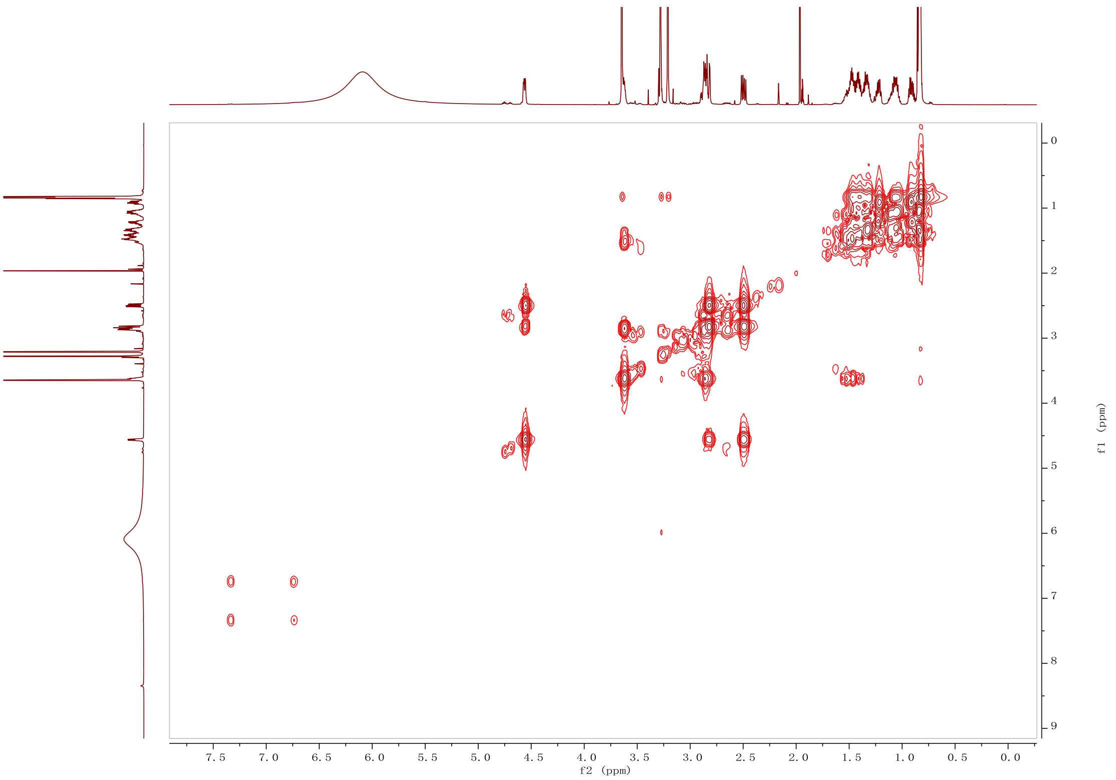

Figure S62. ^1H-^1H COSY of **8** in CD_3CN (600 MHz).

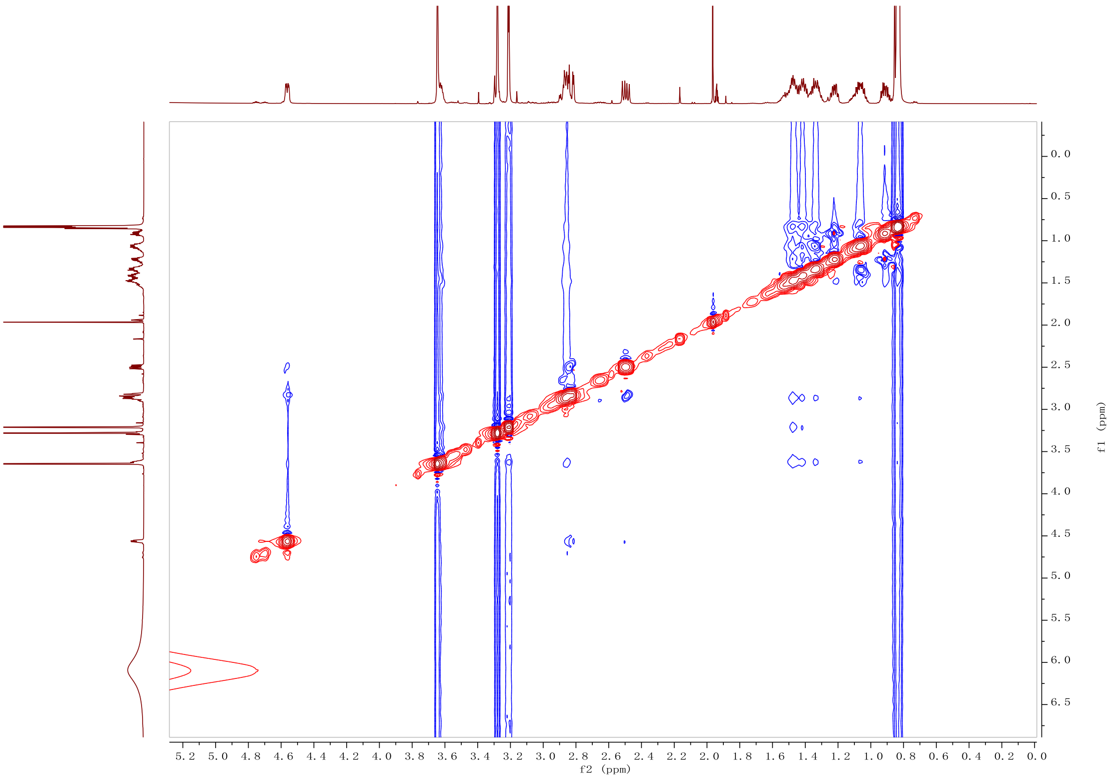

Figure S63. NOESY of **8** in CD_3CN (600 MHz).

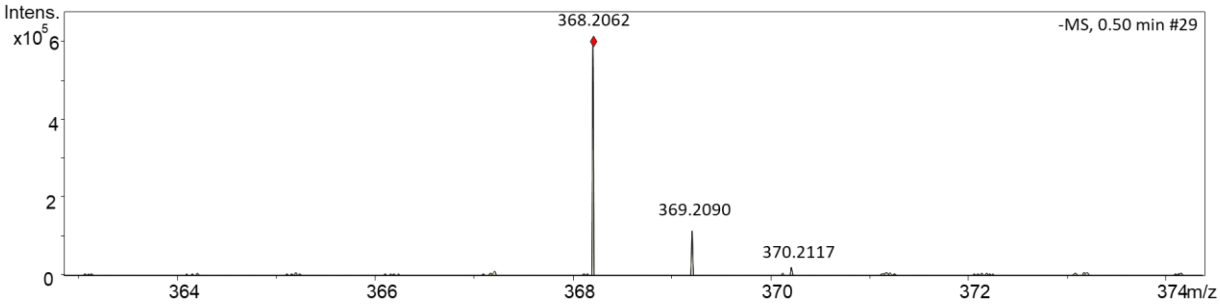

Figure S64. The HRESIMS spectrum of **9**

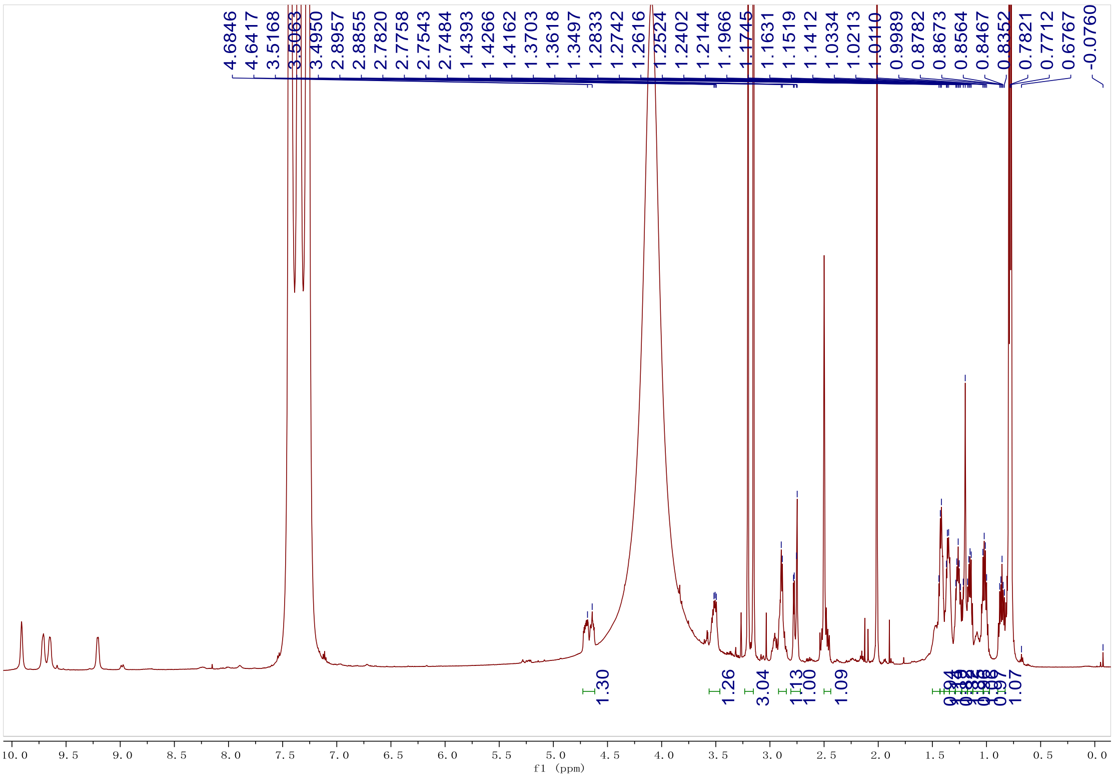

Figure S65. ^{1}H NMR of **9** in DMSO-d_6 (600 MHz).

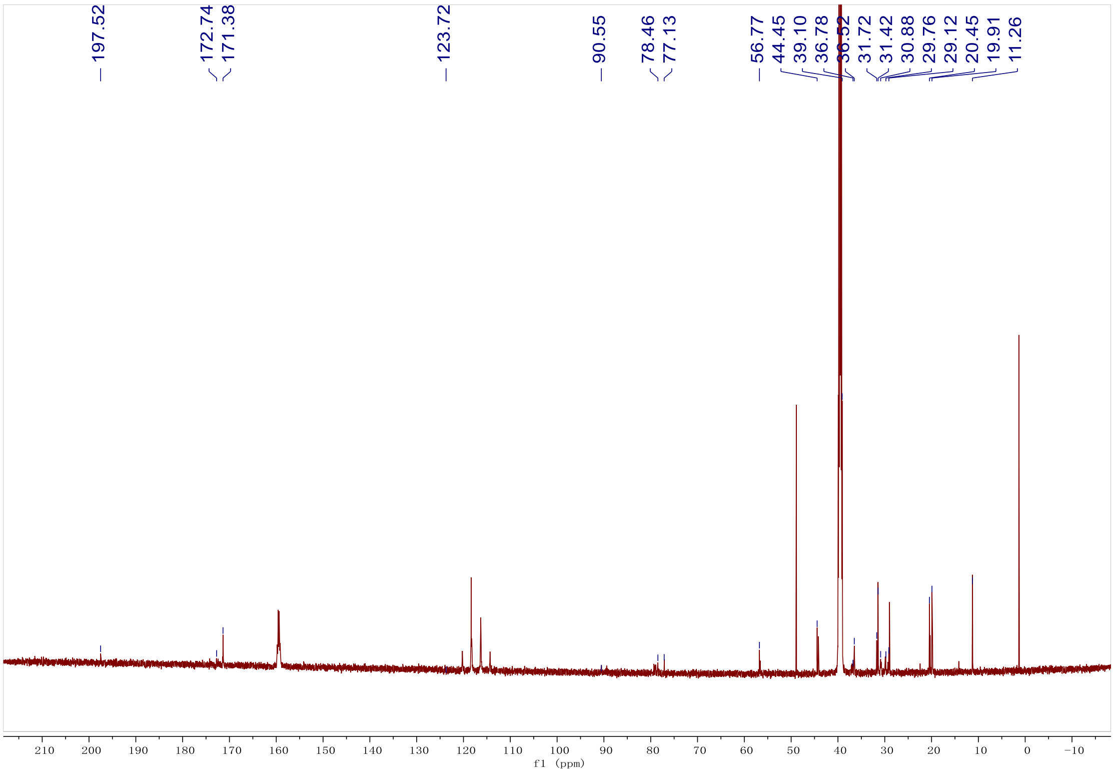

Figure S66. ^{13}C NMR of **9** in DMSO-d_6 (150 MHz).

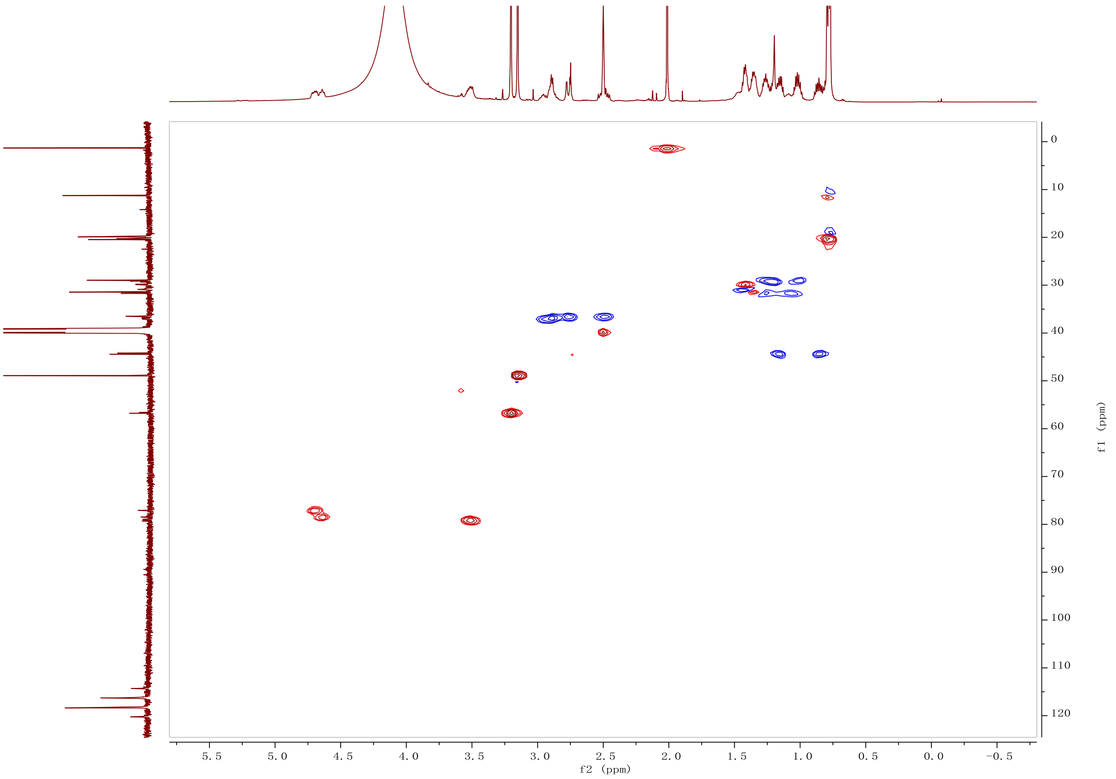

Figure S67. HSQC of **9** in DMSO-*d*$_6$ (600 and 150 MHz).

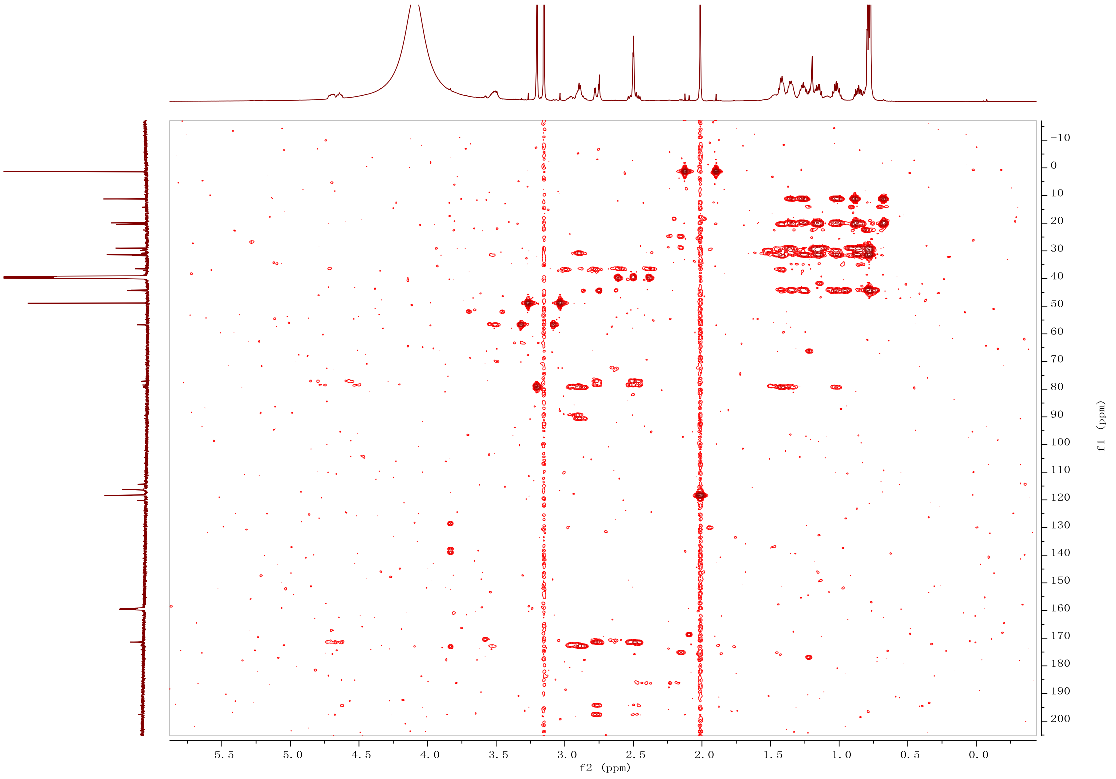

Figure S68. HMBC of **9** in DMSO-*d*$_6$ (600 and 150 MHz).

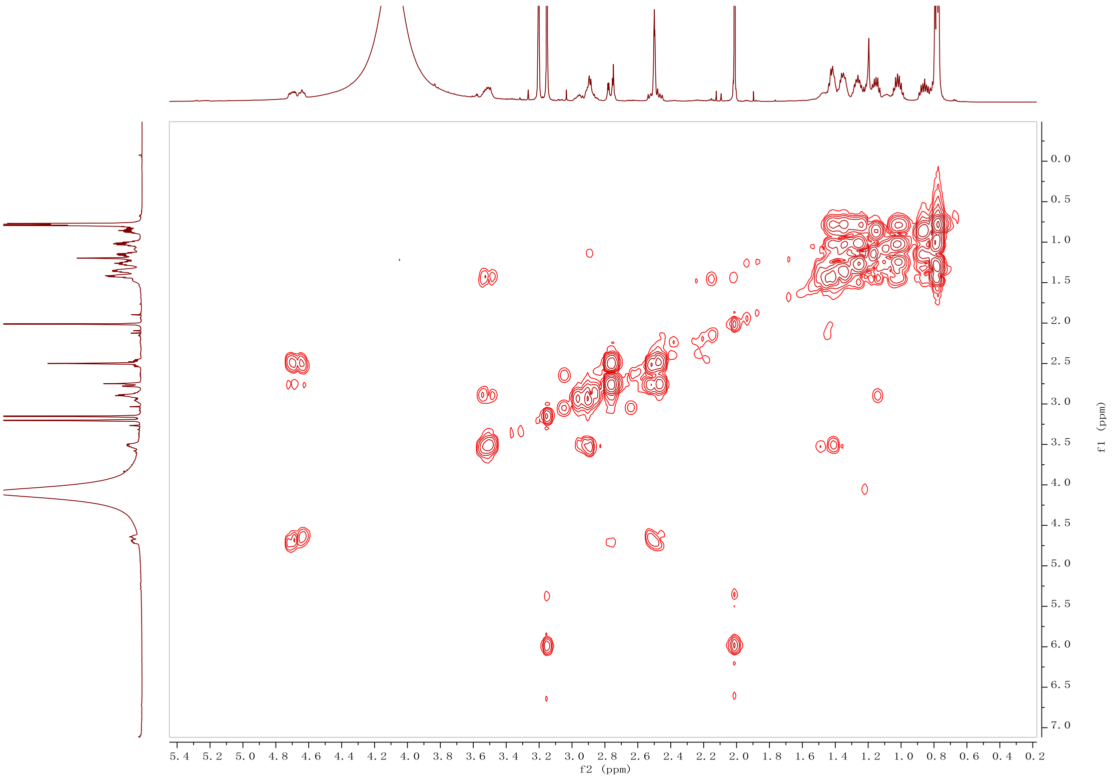

Figure S69. ^{1}H-^{1}H COSY of **9** in DMSO-d_6 (600 MHz).

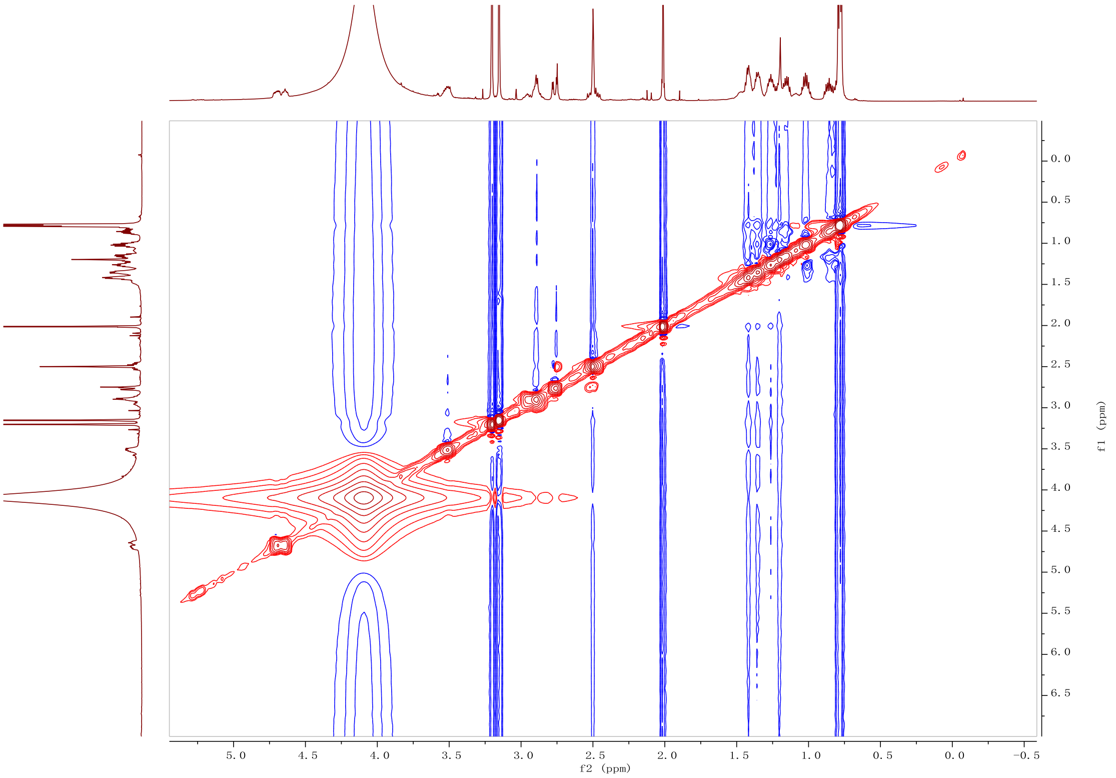

Figure S70. NOESY of **9** in DMSO-d_6 (600 MHz).

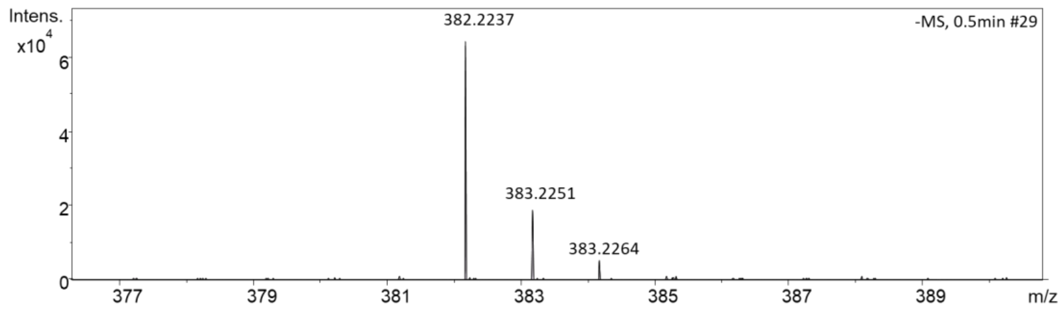

Figure S71. The HRESIMS spectrum of **10**

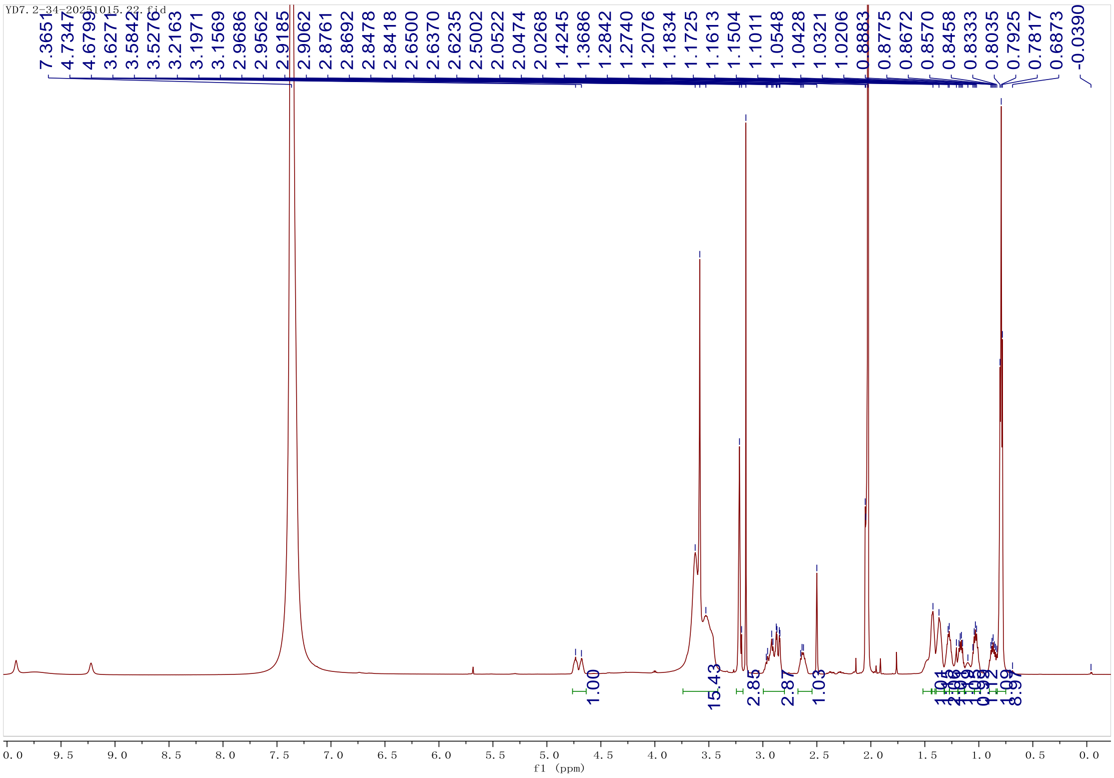

Figure S72. ^{1}H NMR of **10** in DMSO-d_6 (600 MHz).

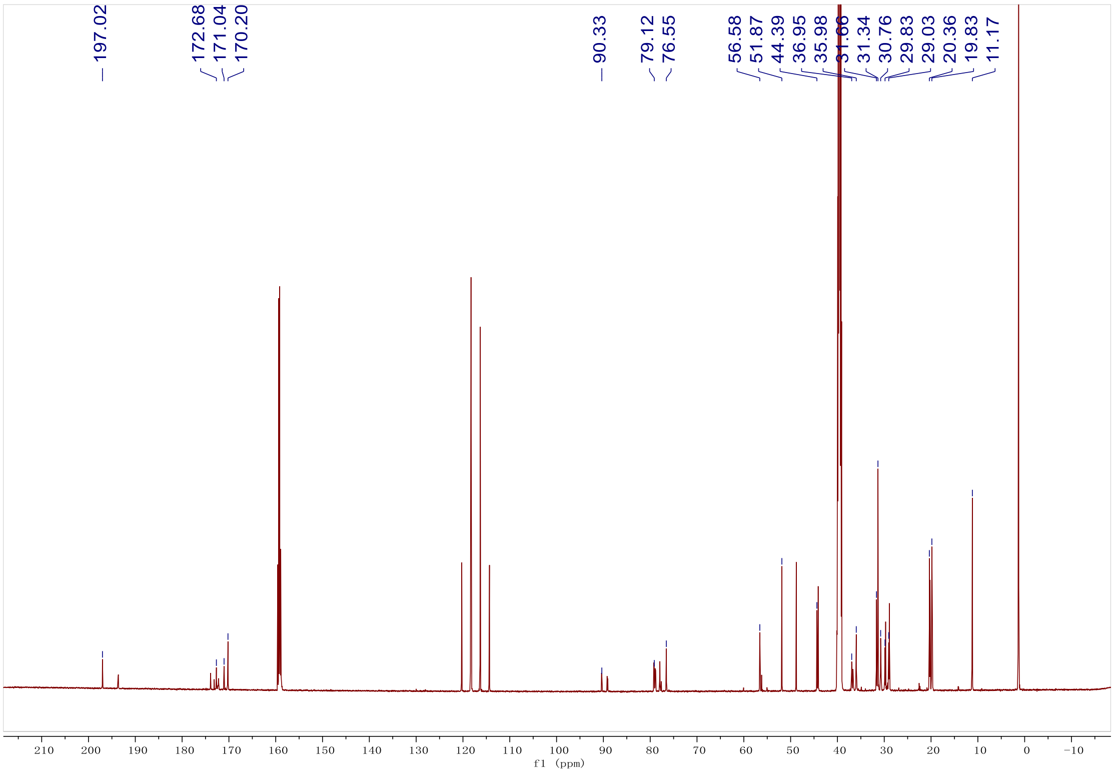

Figure S73. ^{13}C NMR of **10** in DMSO-d_6 (150 MHz).

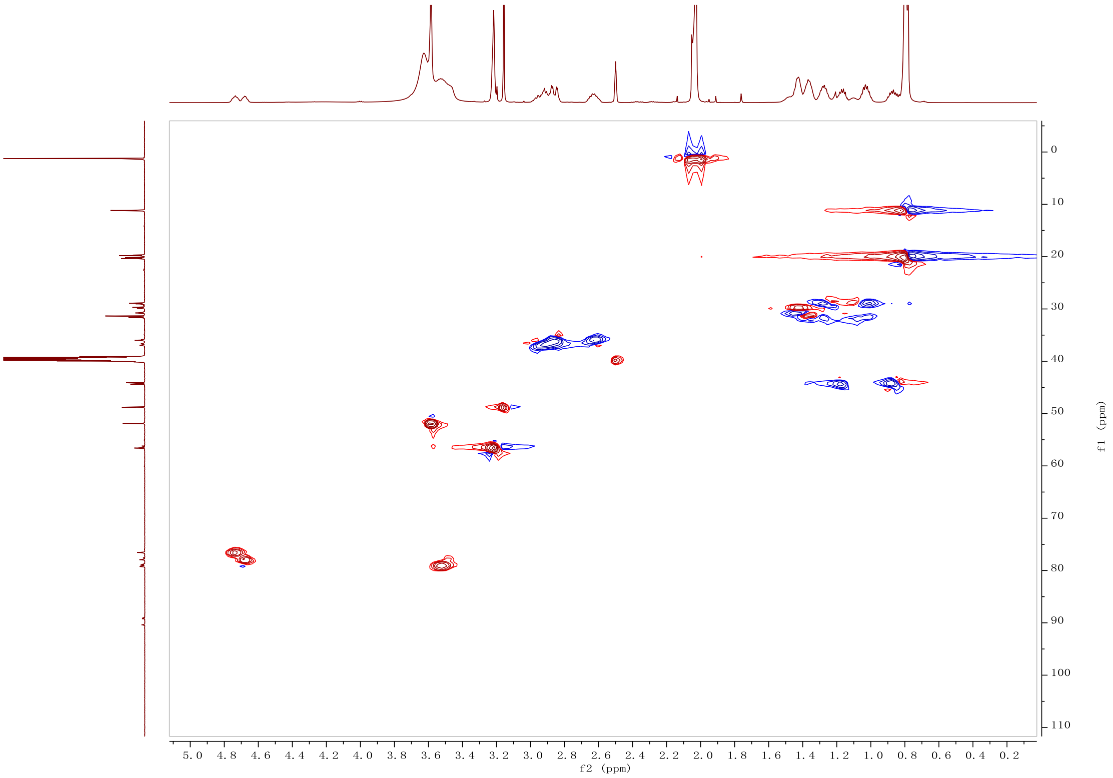

Figure S74. HSQC of **10** in DMSO-d_6 (600 and 150 MHz).

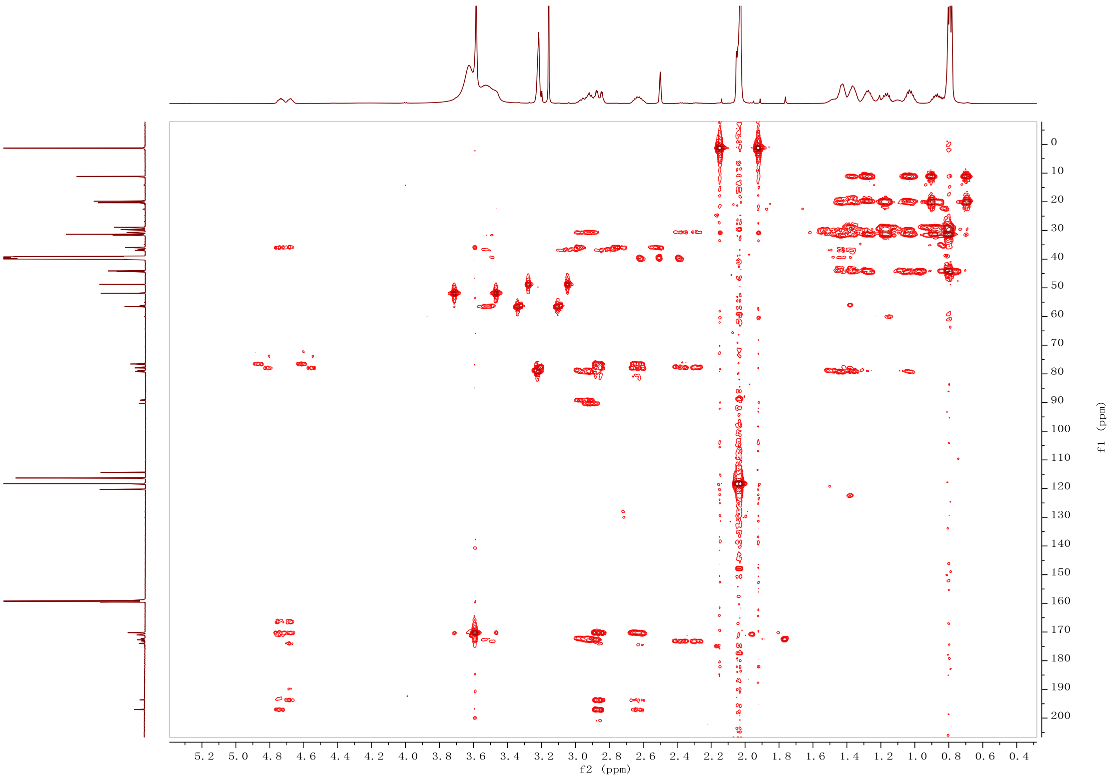

Figure S75. HMBC of **10** in DMSO-d_6 (600 and 150 MHz).

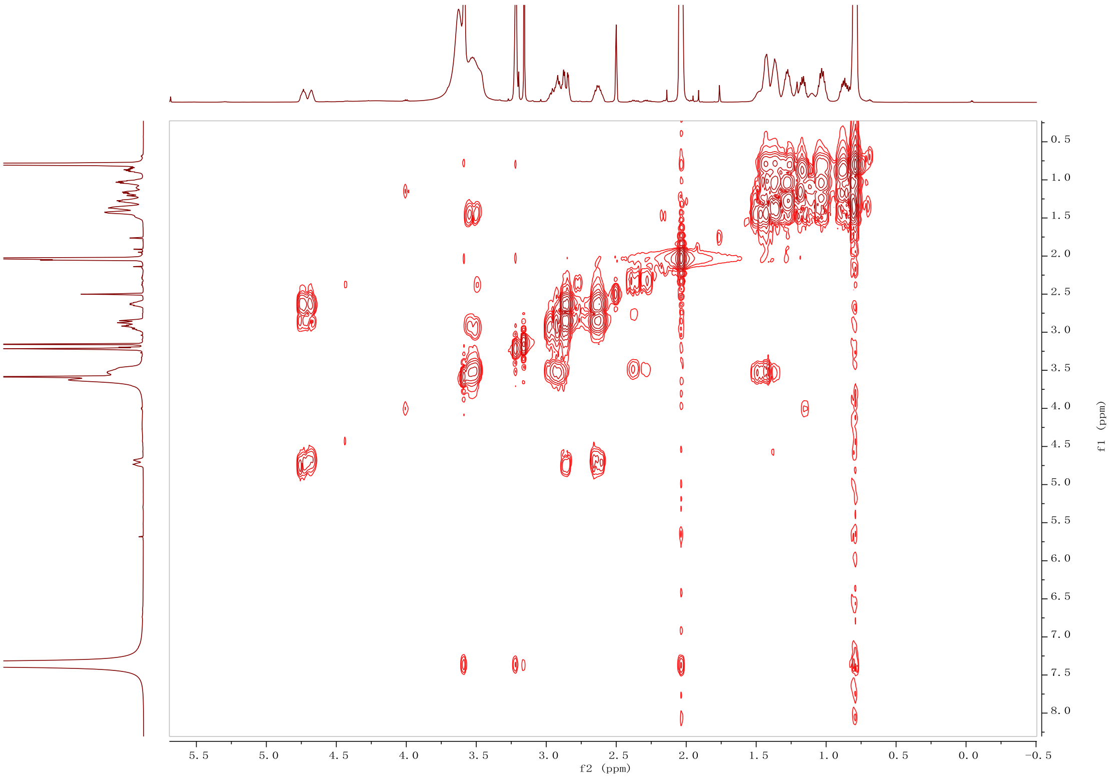

Figure S76. ^{1}H-^{1}H COSY of **10** in DMSO-d_6 (600 MHz).

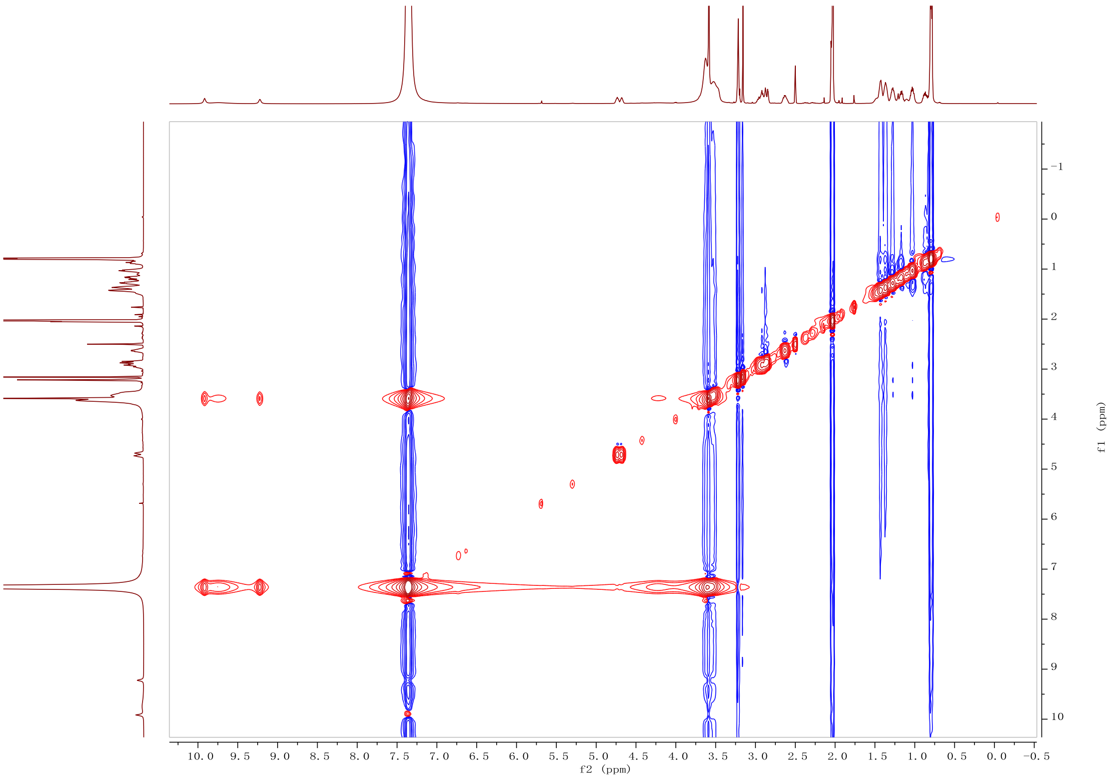

Figure S77. NOESY of **10** in DMSO-d_6 (600 MHz).

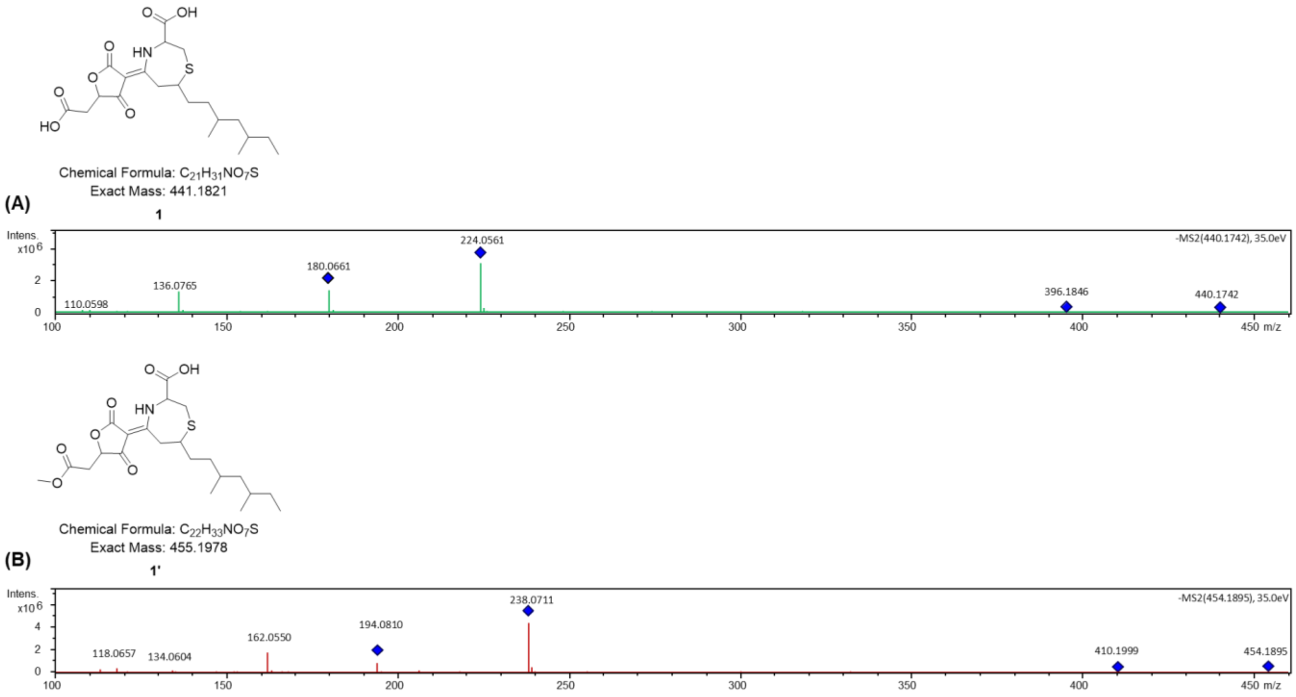

Figure S78. LC-MS/MS spectrum of compounds **1** (A) and **1′** (B).
